# Equation of state for He bubbles in W and model of He bubble growth and bursting near W{100} surfaces derived from molecular dynamics simulations

**DOI:** 10.1038/s41598-023-35803-3

**Published:** 2023-06-13

**Authors:** Wahyu Setyawan, Dwaipayan Dasgupta, Sophie Blondel, Giridhar Nandipati, Karl D. Hammond, Dimitrios Maroudas, Brian D. Wirth

**Affiliations:** 1 Pacific Northwest National Laboratory, Richland, WA 99352 USA; 2 Department of Nuclear Engineering, University of Tennessee, Knoxville, TN 37996 USA; 3 Department of Chemical and Biomedical Engineering, University of Missouri, Columbia, MO 65211 USA; 4 Department of Chemical Engineering, University of Massachusetts, Amherst, MA 01003 USA; 5 Fusion Energy Division, Oak Ridge National Laboratory, Oak Ridge, TN 37830 USA

**Keywords:** Materials science, Physics

## Abstract

Molecular dynamics (MD) simulations are performed to derive an equation of state (EOS) for helium (He) bubbles in tungsten (W) and to study the growth of He bubbles under a W(100) surface until they burst. We study the growth as a function of the initial nucleation depth of the bubbles. During growth, successive loop-punching events are observed, accompanied by shifts in the depth of the bubble towards the surface. Subsequently, the MD data are used to derive models that describe the conditions that cause the loop punching and bursting events. Simulations have been performed at 500, 933, 1500, 2000, and 2500 K to fit the parameters in the models. To compute the pressure in the bubble at the loop punching and bursting events from the models, we derive an EOS for He bubbles in tungsten with an accompanying volume model to compute the bubble volume for a given number of vacancies ($N_\text {V}$), He atoms ($N_\text {He}$), and temperature (*T*). To derive the bubble EOS, we firstly derive the EOS for a free He gas. The derived free-gas EOS can accurately predict all MD data included in the analysis (which span up to 54 GPa at 2500 K). Subsequently, the bubble EOS is derived based on the free-gas EOS by correcting the gas density to account for the interaction between He and W atoms. The EOS for the bubbles is fitted to data from MD simulations of He bubbles in bulk W that span a wide range of gas density and sizes up to about 3 nm in diameter. The pressure of subsurface bubbles at the loop punching events as calculated using the bubble-EOS and the volume model agrees well with the pressure obtained directly from the MD simulations. In the loop punching model, for bubbles consisting of $N_\text {V}$ vacancies and $N_\text {He}$ helium atoms, the $N_\text {He}/N_\text {V}$ ratio that causes the event, the resulting increase in $N_\text {V}$, and the associated shift of the bubble depth are formulated as a function of $N_\text {V}$ and *T*. In the bursting model, a bubble must simultaneously reach a certain depth and $N_\text {He}/N_\text {V}$ ratio in order to burst. The burst depth and $N_\text {He}/N_\text {V}$ are also modeled as a function of $N_\text {V}$ and *T*. The majority of the loop punching events occur at bubble pressures between 20 and 60 GPa, depending on the bubble size and temperature. The larger the bubble and the higher the temperature, the lower the bubble pressure. Furthermore, our results indicate that at a higher temperature, a bubble can burst from a deeper region.

## Introduction

Tungsten (W) is used as the plasma-facing material (PFM) in many designs of fusion energy devices^1,2^ primarily because it exhibits a high melting temperature, a low sputtering yield, a good thermal conductivity, and good mechanical properties at high temperatures. Two main challenges with tungsten PFM are brittleness with a low fracture toughness below the ductile-to-brittle transition temperature (DBTT) and nanotendril-like surface structure formation due to helium (He), a plasma ash in D–T fusion reaction. The DBTT can vary widely as it depends strongly on microstructure, testing direction, and strain rate^3–8^. Values of $\approx$ 300–1000 °C have been reported, and radiation hardening in a fusion reactor environment will further elevate this DBTT range^3,9^. The fracture toughness of as-sintered W below the DBTT is $\approx$ 5, 6, and 11 MPa$\sqrt{\textrm{m}}$ at room temperature, 200 °C, and 400 °C, respectively^3,10^. Excessive exposures of W surfaces to He plasma at temperatures of $\approx$ 900–2000 K lead to the formation of W fuzz^11–15^. For instance, an exposure to He plasma ($5\times 10^{22}$ He/m$^2$/s for 1 h with He energy of $\approx$ 60 eV) produces fuzz with a thickness of $\approx$ 3 µm at 1120 K and 5 µm at 1320 K^13,14^. If this fuzz is released to the plasma, it may seriously degrade the plasma performance^16^.

A fully predictive model describing fuzz formation remains to be established^15,17^. However, studies indicate that surface modification induced by He bubbles plays an important role^11,17^. Scanning electron microscopy (SEM) analysis shows pinholes were observed on the surface at the beginning of plasma exposure, before fuzz eventually formed^11,17^. Large-scale molecular dynamics simulations show that surface roughness and pinholes are created because of the formation, growth, and bursting of He bubbles^18,19^. Transmission electron microscopy (TEM) analysis also shows that the fuzz itself contains He bubbles^20^. Therefore, understanding the growth of subsurface He bubbles is important to model surface evolution of tungsten which leads to fuzz formation. Helium bubbles can grow by a combination of absorbing He and vacancies or, in the case of highly-pressurized bubbles, punching out an interstitial loop (loop punching event) to create additional vacancies. Depending on the proximity to the surface, the He bubbles may burst.

The purpose of this paper is to develop a set of models of loop punching and bubble bursting events based on molecular dynamics (MD) simulations, and our analysis has also led to an improved equation of state (EOS) of He in nanometer-size, high-pressure gas bubbles in W. The models are intended to inform mesoscale simulations of He and damage accumulation in subsurface tungsten, to understand and predict the fuzz layer growth in tungsten PFM. The loop punching event is triggered by the number ratio of He atoms to vacancies ($N_\text {He}/N_\text {V}$), and as a result of the event the number of vacancies in the bubble ($\Delta N_\text {V}$) increases along with displacement of the position of the bubble towards the surface ($\Delta z$). A bubble bursting event is triggered by a combination of factors, including depth the bubble from the free surface and $N_\text {He}/N_\text {V}$. We formulate the models to empirically establish aforementioned causalities and effects in addition to the dependency of both the events on the size of the bubble, $N_\text {V}$. Furthermore, the dependence on temperature, which controls the pressure and in-turn controls the events, is also included in the models.

The models can be reformulated to explicitly study the dependency on the pressure (*i.e.*, the pressure needed to cause loop punching and/or bubble bursting events) simply by employing an EOS. By comparing the pressure obtained from MD with the pressure calculated with the existing EOS for a free He gas^21^ (which was fitted to experimental data over a temperature range of 75–300 K and a pressure range of 0.2–2 GPa), it was apparent that the existing EOS is not applicable to bubbles for the pressure and temperature ranges studied in this article. In fact, the existing EOS over-predicts the MD data for free He gas at high temperatures and pressures (note that the existing EOS agrees very well with the MD pressure at 500 K, even up to 40 GPa). Therefore, a new EOS for free He gas is derived in this study. More importantly, an EOS for He bubbles in W is also developed, namely by modifying the new EOS for free He gas to account for the repulsion between the He and W atoms.

Additional analyses are given to compute and model the probability of partial bursting in which case the bubble cavity is resealed upon bursting by at least one W layer, along with the fraction of the number of He atoms left in the bubble upon partial bursting, the thickness of W segment (ligament) above the bubble just before bursting, and the size (area) of the burst hole. Numerous snapshots of atomic configurations nearing bubble bursting events are included in the Supplementary Information for the benefit of the readers.

The paper is organized as follows. Firstly, models for the loop punching and bubble bursting events are derived using simulations at 933 K. Subsequently, additional MD simulations at 500, 1500, 2000, and 2500 K are performed to incorporate temperature dependence into the models. The derivation of the EOS models for a free He gas and He bubbles in W is then presented. For completeness, a volume model is then derived to compute the bubble volume as a function of $N_\text {V}$, $N_\text {He}$, and *T*. Lastly, the bubble EOS and the volume model are employed to compute the pressure of subsurface He bubbles at the loop punching events and compared to the pressure data obtained directly from MD.

## Method

The molecular dynamics method (MD) was used to simulate the growth of He bubbles near the surface of tungsten until bursting. The MD simulations were performed using the LAMMPS software^22^ with the following interatomic potentials: W–W embedded-atom method (EAM) potential developed by Ackland and Thetford^23^ with a short-range interaction modification by Juslin and Wirth^24^, W–He pair potential by Juslin and Wirth^24^, and He–He pair potential by Beck^25,26^ with a short-range interaction modification by Morishita^27^. This set of interatomic potentials has been fitted to density functional theory (DFT) and experimental data to give good predictions of structural parameters and fundamental defect energetics and kinetics, as well as surface energies; it has been extensively employed in numerous studies of He effects in W^18,19,28–41^.

In each simulation, a single bubble was nucleated in a tungsten slab at a given depth and subsequently grown by inserting He atoms directly in the bubble. Slab structures with 25 Å of vacuum were used with the *x*-, *y*-, and *z*-axes oriented along the bcc [100], [010], and [001] directions, respectively. The $+z$ axis was taken as the surface normal direction. The slabs were constructed based on the lattice parameter of W at the intended temperature as calculated with the above interatomic potentials. The size of the slabs varied based on the initial depth of the bubble. Table 1 summarizes the size of the slab supercells (with respect to a bcc unit cell) and the number of simulations for each initial depth. A bubble was nucleated by creating a vacancy containing 4 He atoms (He$_4$V). The system was then thermalized at the simulation temperature for 200 ps using a Nosé–Hoover thermostat with 0.1 ps damping parameter. Note that since the slab was already constructed with the lattice parameter at the target temperature, relaxing the slab under a constant volume is better than relaxing under constant pressure in preventing an artificial Poisson effect due to relaxation of layers near the surface. The thermalization and subsequent bubble growth simulations were performed using an MD time step of 0.5 fs. This time step was informed through a scoping simulation using an adaptive time step, such that the maximum displacement per step of the fastest atom is $d_\text {max} = 0.02$ Å (which was sufficient to conserve the total energy in the absence of a thermostat).

After the thermalization of the slab containing the bubble nucleus, the growth of the bubble was simulated by inserting a He atom into the bubble every 2 ps. The system was evolved in the absence of a thermostat, except for the atoms within 10 Å of the bottom edge, which remained temperature-controlled at the simulation temperature to model heat dissipation into the bulk. In addition, W atoms on the very bottom layer were fixed at their crystallographic coordinates during the simulations. The He insertion was stopped when the maximum *z* position of the He atoms has reached the initial position of the top layer, or when the bubble pressure has dropped below 5 GPa. We found that the latter criterion was able to detect bubble bursting (and, thus, stop the insertion) earlier than the first one, and thus is more accurate. The pressure (*P*) of the bubble was calculated based on the virial theorem as follows:

1 $$\begin{aligned} P = \frac{1}{3V} \sum _i \left( m_i v_i^2 + \frac{1}{2}\sum _j \textbf{r}_{ij} \cdot \textbf{F}_{ij} \right) \end{aligned}$$

where *i* is the index of He atoms in the bubble, *j* is the index of the neighboring atoms of atom-*i* (including He and W atoms), *m* and *v* are the atomic mass and speed, respectively, $\textbf{F}_{ij}$ is the force vector on atom *i* due to neighbor *j*, $\textbf{r}_{ij}$ is the position vector of atom *i* measured from neighbor *j*, and *V* is the bubble volume. To calculate the pressure during simulations, the bubble volume was calculated based on the Voronoi tessellation as implemented in LAMMPS. To reduce fluctuation, each computed value of *P* and *V* was time-averaged over 1 ps.

**Table 1:** Initial depth of bubbles ($h_0$), supercell size of the slab along *x* (*nx*), *y* (*ny*), and *z* (*nz*), and number of simulations ($n_\mathrm{sim}$).

$h_0$ (*a*/2)	$nx=ny$	*nz*	$n_\mathrm{sim}$
5	15	28	40
9	25	30	40
13	28	32	40
17	36	34	30
21	44	46	25
25	52	53	25
29*	60	55	6

The calculated lattice parameter of W at 933 K is $a=3.1811$ Å. An asterisk denotes that the depth of the bubble nucleated at this location remained approximately the same after 5000 He atoms had been inserted, thus, the simulations were not continued (see text).

## Results

### Loop punching events

**Figure 1:**
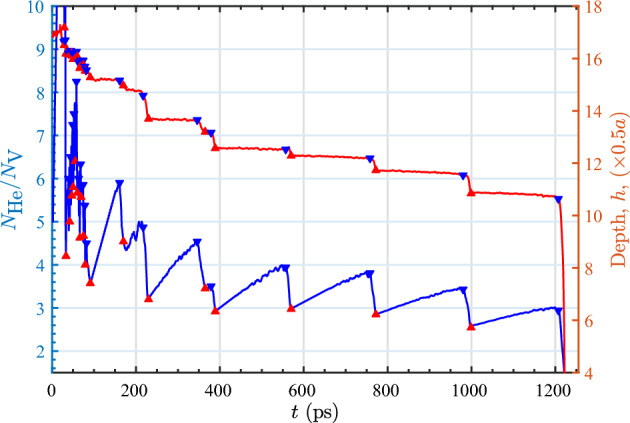
Representative example of evolution of $N_\text {He}/N_\text {V}$ and depth of a bubble nucleated at 17*a*/2 below the top layer. Up and down triangles denote the beginning and end of loop punching events. The last loop punching event corresponds to bubble bursting.

Figure 1 shows representative MD simulation results for the evolution of $N_\text {He}/N_\text {V}$ from one of the bubbles that was nucleated at an initial depth of 17*a*/2. The $N_\text {He}/N_\text {V}$ ratio gradually increases and then suddenly decreases, which is associated with a loop punching event. This behavior is repeated until the last bubble bursting event. It can be seen that $N_\text {He}/N_\text {V}$ at loop punching events successively decreases as the bubble grows. For data analysis, a loop punching event is detected when the decrease in $N_\text {He}/N_\text {V}$ is larger than a specified value ($\eta _\mathrm{d}$, where the subscript ‘d’ stands for detection) which depends on the bubble size. The following setting is found to yield a reliable detection: $\eta _\mathrm{d} = 0.25$ (for $N_\text {V}\le 10$), 0.15 ($N_\text {V}= 11$ to 50), 0.1 ($N_\text {V}\ = 51$ to 150), 0.075 ($N_\text {V}= 151$ to 300), 0.05 ($N_\text {V}= 301$ to 800), and 0.04 ($N_\text {V}> 800$).

In bcc W, a loop is punched out along one of the eight <111> crystallographic directions. In the simulation cell, four directions are toward the surface, and the remaining four are toward the bulk. In the presence of a free surface, the loop is preferentially punched out toward the surface^42^ because of the elastic interaction between the loop and the surface^30–32^. Because of this, as the loop is punched out toward the surface, the bubble shifts upward. For bubbles nucleated at deeper regions, occasionally, a loop was punched out toward the bulk and the bubble shifted deeper into the bulk. Figure 1 shows the successive shifts of bubble depth associated with loop punching events. In our analysis, the depth (*h*) is defined as the distance from the center of mass of the bubble to the initial *z* coordinate of the top layer. For bubbles nucleated at an initial depth of $h_0 = 29a/2$, the bubbles were found to remain at approximately the same depth even after 5000 He atoms had been inserted. Six simulations were performed at this depth and all exhibited the same behavior. This indicates that bubbles nucleated at this depth effectively behave as bubbles in bulk tungsten as opposed to near-surface bubbles. Therefore, further simulations were not performed, and these bubbles were excluded from the analysis. In Table 1, such a depth is denoted with an asterisk.

**Figure 2:**
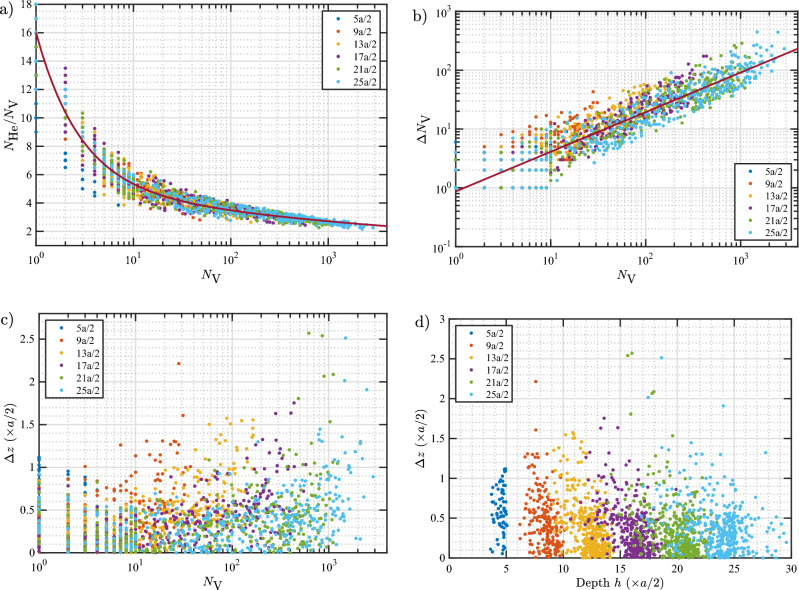
(**a**) $N_\text {He}/N_\text {V}$, (**b**) increase in $N_\text {V}$, and (**c**) displacement of the center of mass of the bubble toward the surface ($\Delta z$) at loop punching events as a function of the number of vacancies. (**d**) $\Delta z$ as a function of bubble depth (*h*) measured from the initial surface position. Data points are colored based on the initial depth of the bubble. Solid lines in (**a**) and (**b**) represent optimal data fits to the data according to the equations $N_\text {He}/N_\text {V}=10.78/N_\text {V}^{0.969}+5.22/N_\text {V}^{0.095}$ and $\Delta N_\text {V}=0.87N_\text {V}^{0.674}$. The average $\Delta z$ over all data points is 0.4*a*/2.

To model the loop punching events, the $N_\text {He}/N_\text {V}$, $\Delta N_\text {V}$, and $\Delta z$ values are plotted as a function of $N_\text {V}$ in Fig. 2 and fitted with analytical functions. General trends are observed for $N_\text {He}/N_\text {V}$ and $\Delta N_\text {V}$ and good fits are obtained as can be seen in the figure (the fit equations are given in the caption). On the other hand, there is no obvious trend seen for $\Delta z$. In most cases, the bubble shifts less than 0.5*a* upward regardless of the size of the bubble (0.5*a* is the inter-planar spacing between adjacent (002) planes). To give a different perspective on $\Delta z$, in Fig. 2d, $\Delta z$ is plotted as a function of the depth at which the loop punching events occur. Again, no definitive trend was observed. Thus, as an approximation, a constant $\Delta z$ can be assumed for the bubble shift. The average $\Delta z$ is 0.4*a*/2. However, for simplicity, a value of 0.5*a*/2 (a half layer) or *a*/2 (a layer) can be taken, depending on the spatial grid used in the mesoscale simulations.

### Bubble bursting events

**Figure 3:**
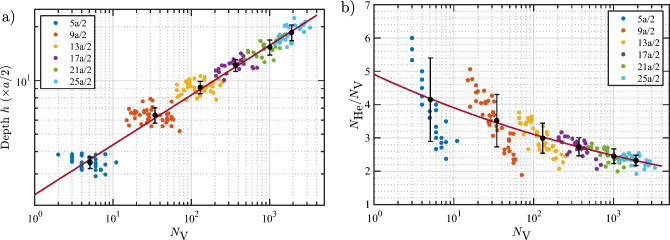
Data at bubble bursting events. (**a**) Bubble final depth *h* and (**b**) $N_\text {He}/N_\text {V}$ as a function of $N_\text {V}$. Data points are colored based on the initial depth of the bubble. Error bars denote standard deviations within each group of bubbles nucleated at the same initial depth. The full date set is used to fit the data. Solid lines represent optimal fits to the data according to the equations $h = 2.30 N_\text {V}^{0.279}$ and $N_\text {He}/N_\text {V}= 4.91/N_\text {V}^{0.099}$.

Regardless of its depth, a bubble of any size can punch a loop when the $N_\text {He}/N_\text {V}$ ratio is high enough. However, as the bubble approaches the surface due to displacement during every loop-punching event and growth of the bubble, it will eventually burst. Thus, in case of bubble bursting, the depth of the bubble at bursting (*h*) is an important factor which needs to be included in the model. Explicitly, for a given $N_\text {V}$, a bubble can burst if it has reached simultaneously critical values of *h* and $N_\text {He}/N_\text {V}$. In Fig. 3, we plot *h* and $N_\text {He}/N_\text {V}$ at bubble bursting events as a function of $N_\text {V}$. A clear correlation is observed, where the burst depth increases with $N_\text {V}$ while the $N_\text {He}/N_\text {V}$ ratio decreases. A power law with one term fits the data fairly well for both *h* and $N_\text {He}/N_\text {V}$ as a function of $N_\text {V}$. The fit functions representing the optimal fits are given in the caption of Fig. 3. Note that since *h*, $N_\text {He}/N_\text {V}$, and $N_\text {V}$ are inter-related, one may equivalently formulate the model in terms of *h* (*i.e.*, for a given depth, a bubble can burst if it reaches a certain value of $N_\text {V}$ and $N_\text {He}/N_\text {V}$). Nevertheless, formulating *h* and $N_\text {He}/N_\text {V}$ in terms of $N_\text {V}$ is more convenient; consequently, the bubble bursting and loop punching models are both formulated in terms of $N_\text {V}$.

In most cases, when a bubble bursts, the burst hole remains open, allowing all He atoms to escape from the bubble cavity to the vacuum above the free surface. However, occasionally, a bubble only partially bursts, in which case one or more W layers reseal the opening before the bubble is completely empty. Partial bursting is observed more frequently for smaller bubbles, presumably because the smaller opening increases the driving force for resealing. To explain the physical origin of this phenomenon, we note that over-pressurized helium bubbles, formed in the near-surface region of tungsten, grow through the absorption of additional helium atoms, trap-mutation reactions, and loop punching until they approach close to the free surface. When a bubble reaches just a few atomic layers below the free surface, due to the high pressure in the bubble, the bubble punches out a small orifice with diameter of a few interatomic distances at the surface and the helium gas is released through that orifice. As the pressure inside the helium bubble begins to decrease, the orifice starts to heal, and the healing process is further accelerated by the curvature-driven diffusion of the W atoms at the edge of the atomic-scale orifice. The competition between the healing of the orifice through the diffusion of W surface atoms and the pressure-driven release of He gas through the orifice determines whether an orifice would heal (partial bursting) or not (total bursting) before releasing all the helium atoms from the bubble. Furthermore, the interstitial loops punched out to the surface during the bubble growth generate island-like surface features (consisting of W adatoms). The orifice often forms at the step edges of these islands, which provide the thermodynamically favorable sites on the surface to nucleate such an orifice; the location of the orifice relative to the island step strongly influences the size and healing mechanism of the orifice and, hence, plays an important role in determining the nature of the bubble bursting. However, exploring such multi-physics bubble bursting and healing mechanisms in full quantitative detail is beyond the scope of this article.

**Figure 4:**
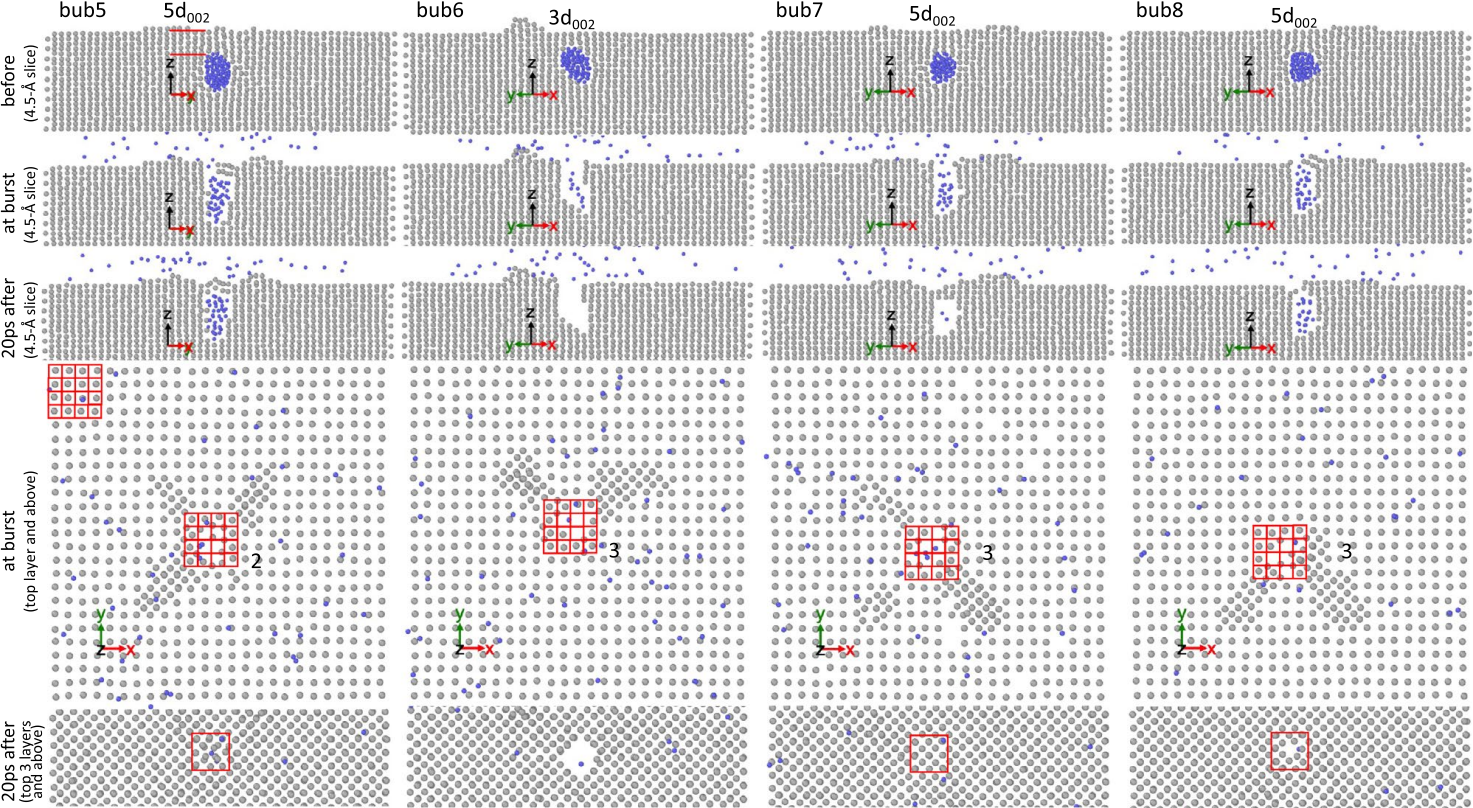
Snapshots of some bubbles, initially nucleated at depth of 9*a*/2, just before bursting (“before”), just after bursting (“at burst”), and at the end of simulations (in this case “20 ps after” bursting). In the fourth panels from the top, snapshots of the top layer and above are shown and the number of vacant sites in the top layer is counted (the grid on the top left shows that each square unit is equal to $a^2$, thus counting the number of empty squares gives the area of the burst hole in units of $a^2$). In the bottom panels, snapshots of the top 3 layers and above are shown. For the cases where the burst hole is resealed, the location of the bubble before bursting is indicated with a large square. Bubbles 5, 7, and 8 partially burst. In case of bubble 8, there is one (002) layer that reseals the bubble as indicated by the red square in the bottom panel. In the top panels, the number of (002) layers above the bubble is counted.

**Figure 5:**
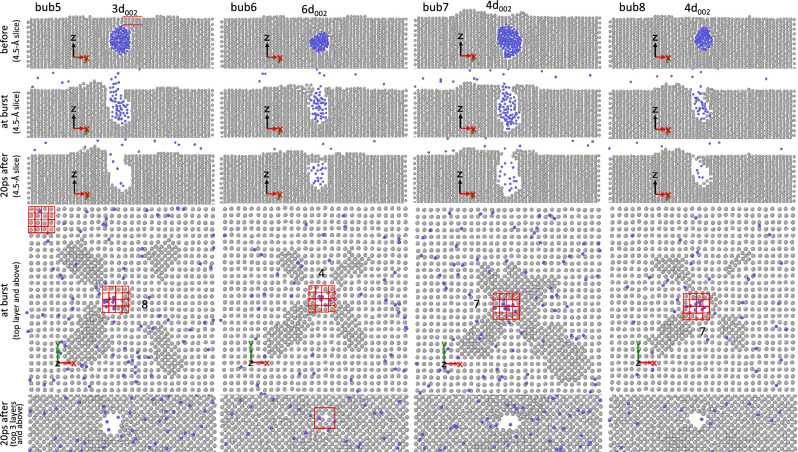
Snapshots of some bubbles, initially nucleated at a depth of 13*a*/2, just before bursting (“before”), just after bursting (“at burst”), and at the end of the simulations (in this case, “20 ps after” bursting).

**Figure 6:**
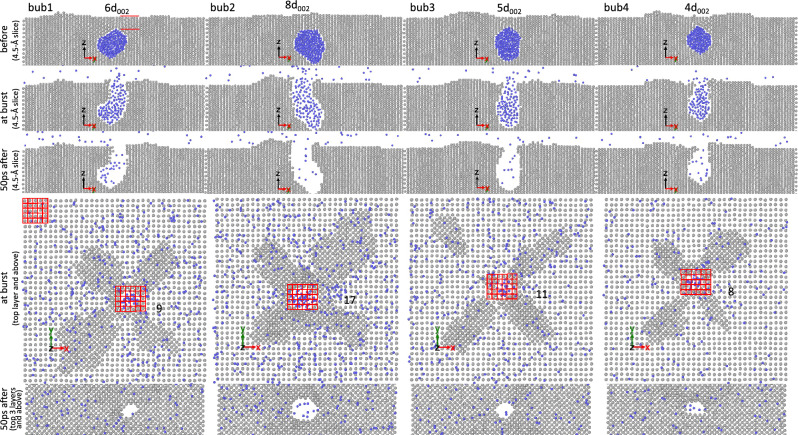
Snapshots of some bubbles, initially nucleated at depth of 17*a*/2, just before bursting (“before”), just after bursting (“at burst”), and at the end of the simulations (in this case, “50ps after” bursting).

Figures 4, 5, 6 show some examples of bubbles that partially burst and others that completely burst. In these figures, snapshots of bubbles just before bursting, just after bursting, and at the end of the simulations are depicted. For brevity, only a few representative examples are presented in the main article. The corresponding snapshots for all bubbles are compiled in Supplementary Figs. 1 to 6. From these snapshots, we analyze the number of W(002) layers above the bubbles just before bursting (denoted as ligament thickness $t_\text {lig}$), the probability of partial bursting ($f_\text {partial}$), the fraction of the number of He atoms remaining in the bubble in partial bursting cases ($f_\text {He}$), the number of the (002) layers that reseal the bubble in the partial bursting cases, and the area of the burst hole ($A_\text {h}$). In our analysis, $A_\text {h}$ is defined as the number of vacant sites in the top layer. Since W atoms in the top layer form a square lattice with a lattice parameter of *a*, $A_\text {h}$ is conveniently given in units of $a^2$.

As we have observed in our simulation results, bubble bursting events can only occur if the bubbles are close enough to the surface, else they will simply punch a loop. Hence, in the following, we present our findings for the three metrics $f_\text {partial}$, $t_\text {lig}$, and $A_\text {h}$, which characterize a bubble bursting event, as a function of the burst depth *h*. Because $N_\text {V}$ at the time of bursting is proportional to *h* (on a log–log scale), any trend in $f_\text {partial}$, $t_\text {lig}$, and $A_\text {h}$ as a function of *h* will be similarly observed as a function of $N_\text {V}$ (readers can readily use the equation given in Fig. 3 to interchange the functional dependence between *h* and $N_\text {V}$).

**Figure 7:**
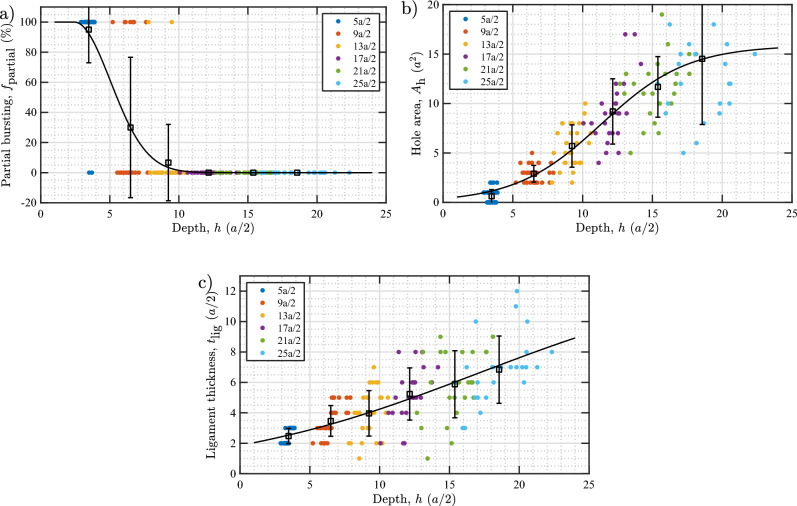
(**a**) Probability of partial bursting ($f_\text {partial}$), (**b**) area of burst hole ($A_\text {h}$), and (**c**) thickness of W ligament above the bubble just before bursting ($t_\text {lig}$) as a function of depth of the bubble at bursting (*h*). Error bars denote standard deviations within each group of bubbles nucleated at the same initial depth. The full data set is used to fit the data. Solid lines represent optimal fits to the data according to the equations $f_\text {partial} = 100 \exp [-0.073(h-2.44)^2]$ for $h>2.44$, else $f_\text {partial} = 100$; $A_\text {h} = 15.88/\bigl [1+\exp \bigl (-0.323(h-11.35)\bigr )\bigr ]$; and $t_\text {lig} = 13.19/\bigl [1+\exp \bigl (-0.106(h-17.04)\bigr )\bigr ]$.

In Fig. 7, the plots of $f_\text {partial}$, $A_\text {h}$, and $t_\text {lig}$ versus *h* are shown. At close proximity to the surface ($h<4a/2$), nearly all the bubbles undergo partial bursting. The probability rapidly decreases to about 40% at $h=6a/2$, and to 10% at $h=8a/2$. At a depth beyond 10*a*/2, no partial bursting is observed. The area of the burst hole increases from about $a^2$ at $h<4a/2$ to $15a^2$ at $h=20a/2$. The ligament thickness increases from about two layers at $h<4a/2$ to eight layers at $h=20a/2$. The equations for the optimal data fits are given in Fig. 7.

### Temperature-dependent loop punching model

To characterize the temperature dependence of the loop punching and bubble bursting processes, simulations of bubble growth from an initial depth of 5*a*/2, 9*a*/2, and 17*a*/2 were performed at 500, 1500, 2000, and 2500 K. The number of independent simulations at these initial depths were 40, 30, and 25, respectively. The corresponding lattice parameters used to construct the slabs at these temperatures were 3.1705 Å (500 K), 3.1984 Å (1500 K), 3.2140 Å (2000 K), and 3.2292 Å (2500 K). Similar to the simulations conducted at 933 K, to determine the time step for simulations at higher temperatures, trial simulations with an adaptive time step were performed with a maximum displacement of $d_{\max } = 0.02$ Å per step. This setting corresponds to a time step of $\approx$ 0.45 fs (1500 K), 0.4 fs (2000 K), and 0.35 fs (2500 K). For simplicity and consistency with the He insertion algorithm (one atom per 2 ps), time steps of 0.5 fs for $T\le 1500$ K and 0.4 fs for $T\ge 2000$ K were subsequently used to run the simulations to generate the corresponding data set.

**Figure 8:**
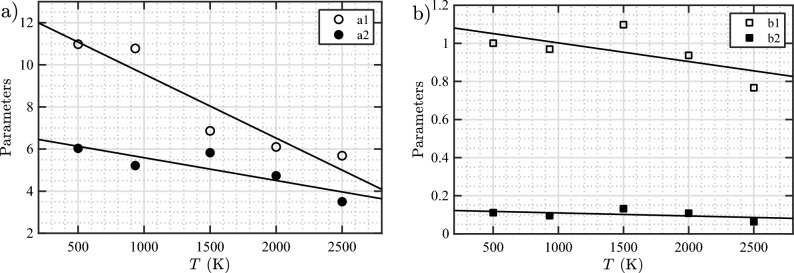
Temperature dependence of parameters in the loop punching model for the $N_\text {He}/N_\text {V}$ (Eq. 2); (**a**) $a_1$ and $a_2$ and (**b**) $b_1$ and $b_2$. Solid lines represent optimal linear fits to the data according to the equations $a_1 = 12.60 - 3.04(T/10^3)$; $a_2 = 6.67 - 1.08(T/10^3)$; $b_1 = 1.100 - 9.78(T/10^5)$; and $b_2 = 0.126 - 1.587(T/10^5)$.

The resulting equation for $N_\text {He}/N_\text {V}$ as a function of $N_\text {V}$ at the loop punching events is parameterized as $N_\text {He}/N_\text {V}= a_1/(N_\text {V})^{b_1} + a_2/(N_\text {V})^{b_2}$, where the two terms are used to capture two different bubble size regimes studied here. The first term is dominant for smaller bubbles, while the second term describes the larger ones. Fitted parameters were then obtained for each temperature. Note that for 933 K, data from all six different initial depths were used in the fitting procedure. In Fig. 8, the fitted parameters $a_1$, $a_2$, $b_1$, and $b_2$ are plotted as functions of temperature. The amplitude parameters $a_1$ and $a_2$ decrease with increasing temperature by a similar percentage from 500 to 2500 K; $a_1$ decreases by 43% (from about 11.0 to 5.7), while $a_2$ decreases by 42% (from 6.0 to 3.5). The exponents $b_1$ and $b_2$ also generally decrease with increasing temperature. From 500 to 2500 K, $b_1$ decreases by about 23% from 1.00 to 0.77, while $b_2$ decreases by 43% from 0.111 to 0.063. Subsequently, polynomial, power, and exponential functions were explored to fit the parameters as functions of temperature, and a linear function was found to be optimal. Thus, we arrive at the following temperature-dependent model for $N_\text {He}/N_\text {V}$ at the loop punching event:

2a $$\begin{aligned} N_\text {He}/N_\text {V}= \,\,& {} a_1/N_\text {V}^{b_1} + a_2/N_\text {V}^{b_2} \end{aligned}$$

2b $$\begin{aligned} a_1= \,\,& {} 12.60 - 3.04(T/10^3) \end{aligned}$$

2c $$\begin{aligned} a_2= \,\,& {} 6.67 - 1.08(T/10^3) \end{aligned}$$

2d $$\begin{aligned} b_1= \,\,& {} 1.100 - 9.78(T/10^5) \end{aligned}$$

2e $$\begin{aligned} b_2= \,\,& {} 0.126 - 1.587(T/10^5) \end{aligned}$$

where *T* is the absolute temperature.

**Figure 9:**
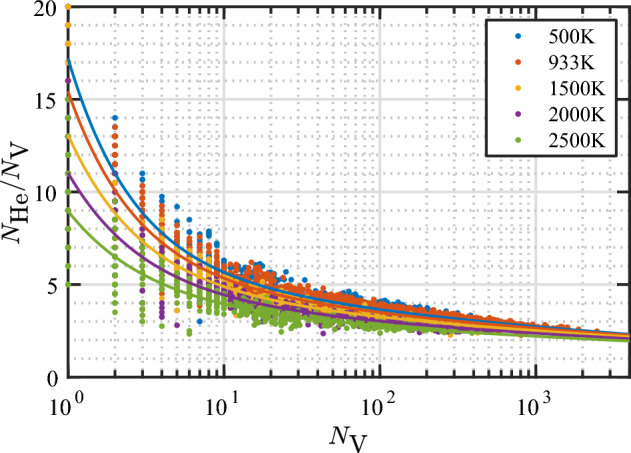
Scatter plots of $N_\text {He}/N_\text {V}$ versus $N_\text {V}$ at loop punching events at various temperatures, along with the model (Eq. 2) predictions represented by the solid lines. Separate plots for each temperature are given in the Supplementary Fig. 7.

In the model, the parameters in the first term exhibit a stronger temperature dependence compared to the corresponding ones in the second term. This signifies that loop punching events from small bubbles are more sensitive to temperature than those from large bubbles. The sum of the values of the parameters $a_1$ and $a_2$ represents the number of He atoms required to initiate a loop punching event from a bubble with a single vacancy. In this case, these numbers are about 17 (at 500 K), 15 (933 K), 13 (1500 K), 11 (2000 K), and 9 He atoms (2500 K). Figure 9 shows the scatter plots of $N_\text {He}/N_\text {V}$ versus $N_\text {V}$ at all the temperatures studied in this work, along with the model (Eq. 2). It can be seen that the model describes the data reasonably well.

**Figure 10:**
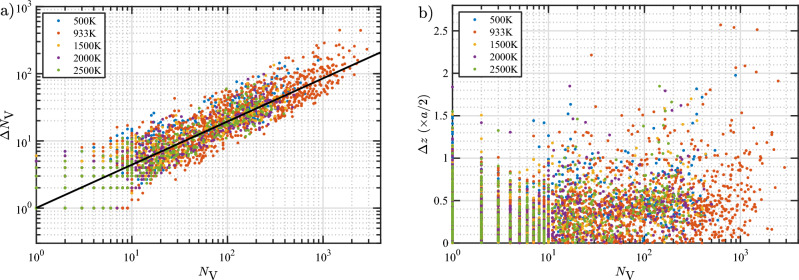
Scatter plots of (**a**) $\Delta N_\text {V}$ and (**b**) $\Delta z$ versus $N_\text {V}$ at loop punching events for all temperatures. The solid line in (**a**) is an optimal fit to the data according to the equation $\Delta N_\text {V}= 1.01 N_\text {V}^{0.643}$. The average $\Delta z$ over all the data is 0.4*a*/2.

We follow the same procedure to derive the temperature-dependent model for the increase in the bubble size ($\Delta N_\text {V}$) at loop punching events. However, from the scatter plots shown in Fig. 10a, the data points from various temperatures appear to overlap with each other within the spread of the data. The results indicate that the size of the emitted loop at a loop punching event does not depend on temperature. Hence, we used a universal fitting procedure involving all the data sets and the optimally fitted model for emitted loop size as function of bubble size $N_\text {V}$ is

3 $$\begin{aligned} \Delta N_\text {V} = 1.01N_\text {V}^{0.643}. \end{aligned}$$

It is intriguing that the fitted exponent of 0.643 is close to 2/3, which implies proportionality with surface area.

Scatter plots of the displacement of the center of mass of the bubbles at loop punching events as a function of $N_\text {V}$ for all temperatures are also insensitive to temperature (Fig. 10b). This is consistent with the lack of temperature dependence for $\Delta N_\text {V}$, because the shift of the bubble position is related to the number of vacancies that is created at the loop punching event. In addition, as previously observed in the simulations at 933 K, the data for $\Delta z$ at other temperatures do not exhibit any correlation with the bubble size. Therefore, we conclude that the displacement of the bubble at the loop punching event is not dependent on temperature or $N_\text {V}$. The average $\Delta z$ over the full data set is 0.4*a*/2.

### Temperature-dependent bubble bursting model

**Figure 11:**
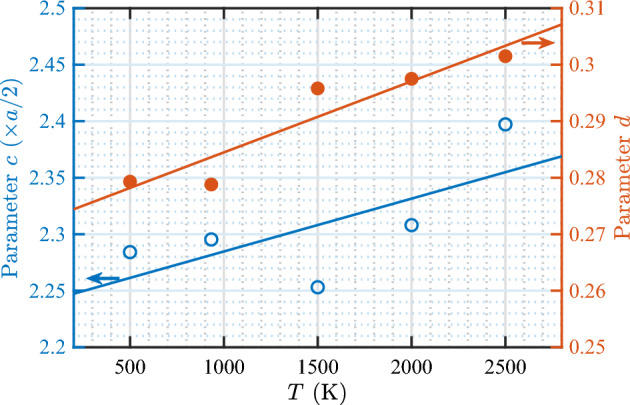
Temperature dependence of parameters in the bubble bursting model for the burst depth *h* (Eq. 4). The solid lines represent optimal linear fits to the corresponding data sets according to the equations $c = 2.24 + 4.68(T/10^5)$; and $d = 0.272 + 1.26(T/10^5)$.

Next, we study the influence of temperature on the bubble bursting process. Data for the depth of the bubble at the time of burst, *h*, versus $N_\text {V}$ were fitted according to the function $h = c N_\mathrm{V}^d$, which was previously shown to describe the data at 933 K very well. The parameters *c* and *d* were then determined for each temperature used in this study. Figure 11 shows parameters *c* and *d* as a function of temperature. Both parameters increase with increasing temperature, although the value of *c* at 1500 K is lower than that at 500 and 933 K, and the value of *d* at 933 K is slightly lower than that at 500 K. As we show below, both parameters are only weakly dependent on temperature. Therefore, deviations of some data points from the general trend can be expected. Nevertheless, the overall trend is evident. Subsequently, a linear fit function is used to capture the general trend of *c* and *d* with temperature. The resulting temperature-dependent bubble bursting model for *h* as a function of bubble size is:

4a $$\begin{aligned} h= \,\,& {} cN_\mathrm{V}^d \end{aligned}$$

4b $$\begin{aligned} c= \,\,& {} 2.24 + 4.68(T/10^5) \end{aligned}$$

4c $$\begin{aligned} d= \,\,& {} 0.272 + 1.26(T/10^5) \end{aligned}$$

where *c* and *h* are given in units of *a*/2.

**Figure 12:**
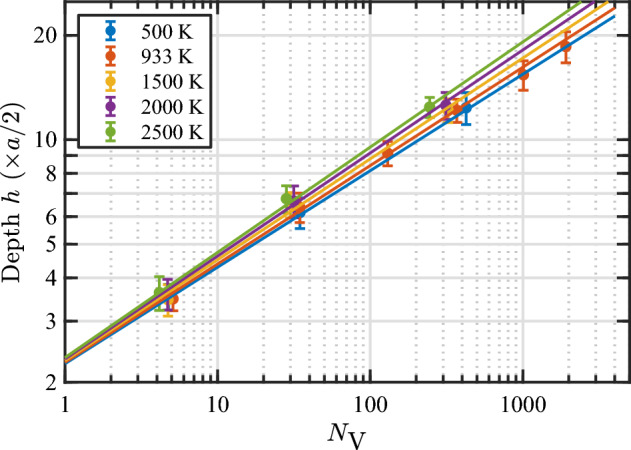
Average values and standard deviations of *h* as a function of $N_\text {V}$ at bubble bursting events at various temperatures, along with the model (Eq. 4) predictions represented by the solid lines. The average values and standard deviations are calculated within each group of bubbles nucleated at the same initial depth. Note that the full data set (not the average values) is used to fit the data. The scatter plots of the full data set for each temperature are given in the Supplementary Fig. 8.

In the model (Eq. 4), the weak dependence of *c* and *d* on temperature is evident: the values of these parameters increase by only a few percentage points for every 1000 kelvins. Figure 12 displays the data of *h* versus $N_\text {V}$ for various temperatures, along with the model predictions. It is evident in the figure that the model describes the relationship very well. In addition, it can be seen that *h* for large bubbles is more sensitive to temperature than that for small bubbles. For instance, increasing the temperature from 500 to 2500 K increases the burst depth by less than one (002) layer thickness (*a*/2) for bubbles smaller than 10 vacancies (*i.e.*, practically no change), by two layers for bubbles with $10^2$ vacancies ($\approx$ 1.5 nm in diameter), and by four layers for bubbles with $10^3$ vacancies ($\approx$ 3.1 nm in diameter). Regardless of the different sensitivities of *h* for bubbles with different sizes, the model clearly reveals that for a given size, the bubble can burst from a relatively deeper position at a higher temperature.

**Figure 13:**
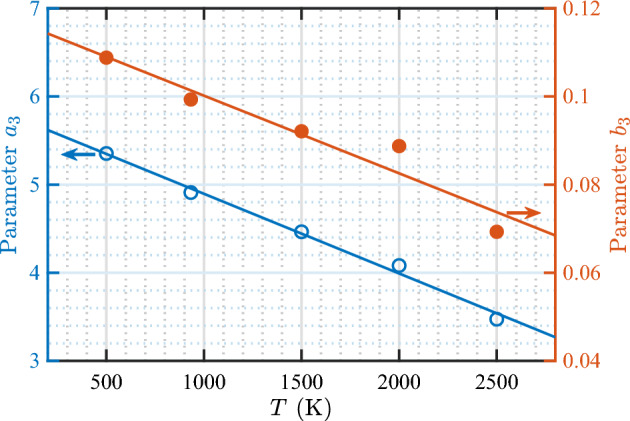
Temperature dependence of parameters in the bubble bursting model for the bursting $N_\text {He}/N_\text {V}$ (Eq. 5). The solid lines represent optimal linear fits to the data according to the equations $a_3 = 5.80{-}9.04(T/10^4)$ and $b_3 = 0.118{-}1.762(T/10^5)$.

As it is done in the case of the loop punching model, the temperature-dependent bubble bursting model for $N_\text {He}/N_\text {V}$ at the time of bursting is parameterized with a power law; $N_\text {He}/N_\text {V}= a_3/N_\text {V}^{b_3}$. In this case, a single term was found to be sufficient to fit the data (adding a second term did not improve the fit). A good fit was obtained for each temperature. Figure 13 shows the fitted values of $a_3$ and $b_3$ as a function of temperature. Both parameters decrease linearly with increasing temperature. Only the $b_3$ values at 2000 and 2500 K show minor deviation from the linear trend, but still follow a monotonic trend. Subsequently fitting $a_3$ and $b_3$ versus temperature with a linear function, we obtain the temperature-dependent model for $N_\text {He}/N_\text {V}$ at bubble bursting events as

5a $$\begin{aligned} N_\text {He}/N_\text {V}= a_3/N_\text {V}^{b_3} \end{aligned}$$

5b $$\begin{aligned} a_3= 5.80{-}9.04(T/10^4) \end{aligned}$$

5c $$\begin{aligned} b_3= 0.118{-}1.762(T/10^5) \end{aligned}$$

where all parameters are dimensionless. Parameter $a_3$ decreases by almost twofold with increasing temperature by 1000 degrees, while $b_3$ is only weakly dependent on temperature (decreases only by $\approx$ 2% for every increase in temperature by 1000 K).

**Figure 14:**
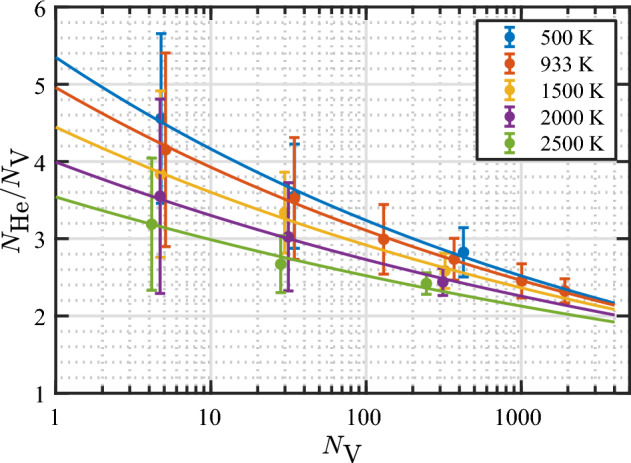
Average values and standard deviations of $N_\text {He}/N_\text {V}$ as a function of $N_\text {V}$ at bubble bursting events at various temperatures, along with the model (Eq. 5) predictions represented by the solid lines. The average values and standard deviations are calculated within each group of bubbles nucleated at the same initial depth. Note that the full data set (not the average values) is used to fit the data. The scatter plots of the full data set for each temperature are given in the Supplementary Fig. 9.

Figure 14 shows the data of $N_\text {He}/N_\text {V}$ at bursting as a function of bubble size for various temperatures, along with the model curves (Eq. 5). It is evident that the model describes the data very well. It can be seen that the $N_\text {He}/N_\text {V}$ ratio at bursting is sensitive to temperature, particularly for small bubbles. Increasing the temperature from 500 to 2500 K decreases the $N_\text {He}/N_\text {V}$ ratio as follows: from 4.2 to 3.0 ($-29\%$) for $N_\text {V}= 10$, 3.2 to 2.5 ($-22\%$) for $N_\text {V}= 100$, and 2.5 to 2.1 ($-16\%$) for $N_\text {V}= 1000$.

### Equation of state of He bubbles in W

In this section, we derive an EOS for He bubbles in W. We follow a similar approach that was successfully employed in the derivation of an EOS for xenon bubbles in uranium dioxide^43^. Firstly, we derive an EOS for a free He gas. Subsequently, the free-gas EOS is modified for He bubbles in W by correcting the He gas density to account for the interaction between He and W atoms.

To derive the EOS for free He gas, we performed thermalization simulations (*NVT* ensemble) of He atoms contained in a $4~\text {nm}\times 4~\text {nm}\times 4$ nm simulation box at $T = 500$, 933, 1500, 2000, or 2500 K. The box was randomly filled with He atoms corresponding to a gas density ($\rho$) up to about 190 He/nm$^3$. For each ($T,\rho$), the simulation was performed for 400 ps. Pressure (*P*) was sampled every 50 fs, time-averaged every 10 ps, and saved to a file. It was found that the pressure was well-equilibrated within 300 ps. Thus, pressure data in the subsequent 100 ps were averaged and taken as the final pressure.

**Figure 15:**
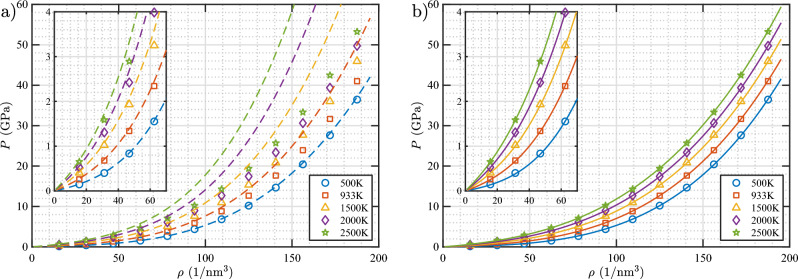
Pressure versus gas density of a free He gas at various temperatures. In (**a**), the dashed lines are isotherms according to the EOS-MLB^21^. In (**b**), the solid lines are isotherms according to the EOS derived in this study (Eq. 6).

The *P* versus $\rho$ plots for a free He gas are shown in Fig. 15a, along with the existing Mills–Liebenberg–Bronson EOS (EOS-MLB)^21^. The EOS-MLB was fitted to experimental data of a He gas in the pressure range of 0.2–2 GPa and temperature range of 75–300 K. From the figure, it is evident that the agreement between the EOS-MLB and MD data at 500 K is excellent, even up to the highest $\rho$ (which corresponds to a pressure of $\approx$ 37 GPa at this temperature). However, the EOS-MLB overpredicts the MD pressure at higher temperatures and densities. Therefore, we derive a new EOS based on a virial EOS fitted to the MD data as follows:

6a $$\begin{aligned} P= \,\,& {} \rho kT (1+B\rho _\mathrm{r} + C\rho _\mathrm{r}^2 + D\rho _\mathrm{r}^3) \end{aligned}$$

6b $$\begin{aligned} B= \,\,& {} b_0 + b_1/T_\mathrm{r} + b_2/T_\mathrm{r}^2 \end{aligned}$$

6c $$\begin{aligned} C= \,\,& {} c_0 + c_1/T_\mathrm{r} + c_2/T_\mathrm{r}^2 \end{aligned}$$

6d $$\begin{aligned} D= \,\,& {} d_0 + d_1/T_\mathrm{r} + d_2/T_\mathrm{r}^2 \end{aligned}$$

6e $$\begin{aligned} \rho _\mathrm{r}= \,\,& {} \rho /\rho _\mathrm{c} \end{aligned}$$

6f $$\begin{aligned} T_\mathrm{r}= \,\,& {} T/T_\mathrm{c} \end{aligned}$$

where *k* is the Boltzmann constant of $1.380649\times 10^{-5}$ GPa nm$^3$/K, $\rho _\mathrm{r}$ and $T_\mathrm{r}$ are the reduced density and temperature, and $\rho _\mathrm{c}$ and $T_\mathrm{c}$ are the density and temperature of He at the critical point (10.419 nm$^{-3}$ and 5.195 K, respectively^44^). Even though it is not used in the above equations, the pressure at the critical point is 227.46 kPa^44^. With the density and temperature expressed in a reduced form, all parameters in the virial coefficients are dimensionless. The optimally fitted parameter values are summarized in Table 2. Note that adding a fifth virial term in the EOS did not improve the fitting, thus, we concluded that the above four-term virial EOS is sufficient for our study. In Fig. 15b, the pressure calculated using the new EOS is plotted as a function of the gas density for various temperatures. The MD data are superimposed in the plot to demonstrate how well the new EOS describes the MD data. It is evident that the new EOS accurately predicts the MD data at all temperatures and densities considered here. The highest density in the MD data corresponds to a pressure of approximately 54 GPa at 2500 K. Given how well the new EOS fits the data, it can be expected to remain applicable to pressures above 54 GPa.

**Table 2:** Fitted parameters $b_i$, $c_i$, and $d_i$ in the virial EOS for a free He gas (Eq. 6), and $f_i$ in the $\Delta R$ formula for the bubble EOS (Eq. 7).

*i*	$b_i$	$c_i$	$d_i$	$f_i$
0	0.09683	0.002676	− 0.0001442	$-0.009508$
1	11.6	4.761	0.1616	$2.626\times 10^{-7}$
2	− 975.5	− 94.62	4.283	0.0004175
3				$1.684\times 10^{-7}$

Based on the He–W interatomic potential used in this study, which was fitted to DFT data, the interaction between He and W is repulsive at all distances. The repulsion will give rise to additional pressure in the bubble, compared to a free gas. To inform how the free-gas EOS can be modified for bubbles, we performed thermalization simulations of an isolated He bubble in bulk W as a function of bubble diameter, gas density, and temperature. To construct a bubble, a void with a specified diameter was created in a bulk bcc W supercell (the lattice parameter at 933 K was arbitrarily used for this construction, but this choice does not affect the results), and subsequently filled with a prescribed number of He atoms. Table 3 summarizes the number of vacancies for each bubble size, the number of He atoms, and the size of the bcc supercell used for the derivation of the bubble EOS.

Each system was thermalized for 400 ps under zero external pressure (Nosé–Hoover thermostat plus barostat) at 500, 933, 1500, 2000, and 2500 K. Bubble pressure was computed from the atomic stresses (Eq. 1) with the bubble volume calculated based on the Voronoi tessellation method. The pressure was sampled every 50 fs, time-averaged every 10 ps, and saved to a file. As in the case with a free gas, the pressure was sufficiently equilibrated within 300 ps. Thus, pressure data in the following 100 ps were averaged and taken as the final pressure.

**Table 3:** Void diameter (*D*), $N_\text {V}$, $N_\text {He}$, and size of bcc supercell (given in multiple of *a*, where *a* is the lattice parameter) used to construct an isolated He bubble in bulk W for the derivation of an EOS for He bubbles.

Label	*D* (nm)	$N_\text {V}$	$N_\text {He}$	Supercell
D1nm	1	34	10, 20, ..., 120	$33 \times 33 \times 33$
D1.5nm	1.5	108	30, 60, ..., 300	$35 \times 35 \times 35$
D2nm	2	266	50, 100, ..., 600	$37 \times 37 \times 37$
D3nm	3	886	200, 400, ..., 2400	$41 \times 41 \times 41$

As mentioned, the EOS for the bubbles is derived based on the new free-gas EOS, but by modifying the gas density to account for the W–He repulsion. The gas density ($\rho$) is calculated from the unmodified bubble gas density ($\rho _\mathrm{B}$) as follows:

7a $$\begin{aligned} \rho= \,\,& {} \rho _\mathrm{B} \left( \frac{R}{R+\Delta R}\right) \end{aligned}$$

7b $$\begin{aligned} \Delta R= \,\,& {} (f_0 + f_1T_\mathrm{r})\rho _\mathrm{Br} + (f_2 + f_3T_\mathrm{r})\rho _\mathrm{Br}^2 \end{aligned}$$

7c $$\begin{aligned} \rho _\mathrm{Br}= \,\,& {} \rho _\mathrm{B}/\rho _\mathrm{c} \end{aligned}$$

where *R* is the bubble radius calculated from the bubble volume assuming a spherical bubble. Subsequently, the parameters $f_0$, $f_1$, $f_2$, and $f_3$ are fitted and the values are given in Table 2.

**Figure 16:**
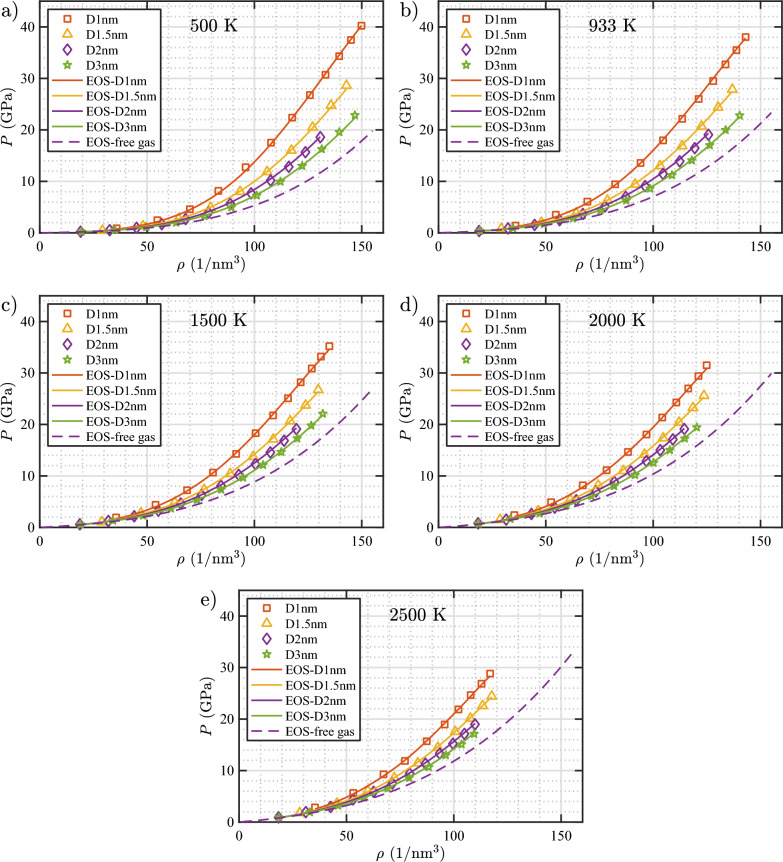
Pressure versus gas density isotherms of He bubbles in bulk W (see Table 3 for information on the labeling of the bubbles). Data points are obtained from MD simulations. Solid lines are isotherms calculated using the bubble EOS (Eqs. 6 and 7), and dashed lines are isotherms calculated using the EOS for a free gas (Eq. 6).

Figure 16 shows the *P* versus $\rho$ plots for the bubbles as obtained from the simulations, along with the curves calculated with the bubble EOS and the free-gas EOS derived in this study. As evident from the figure, the bubble EOS accurately predicts the pressure for all bubble sizes, bubble gas densities, and temperatures considered in this study. In addition, it can be clearly seen that the free-gas EOS underestimates the bubble pressure as expected because it does not account for the W–He repulsion.

So far, we have derived the EOS for the bubbles. In order to use the EOS to calculate the pressure at the loop punching and/or bubble bursting events, we need to derive a model to compute the bubble volume *V* as a function of $N_\text {V}$, $N_\text {He}/N_\text {V}$, and *T*. A simple way to define a volume is by multiplying the number of vacancies with the atomic volume of a W atom in a bcc lattice ($\Omega = 0.5a^3$), that is, $V = N_\text {V}\Omega$. However, since the interaction between He and W atoms is not a hard-wall interaction (*i.e.*, zero for distances greater than a certain distance, and infinite elsewhere), the space explored by the He atoms (effective volume) inside the bubble should be expected to also depend on the gas density and temperature (a hypothesis that will be clearly demonstrated at the end of this section).

Conveniently, our bulk bubbles were constructed based on a fixed number of vacancies (for each “approximate” bubble size). This allows us to study the dependence of volume on density only (for a given set of bubbles with the same $N_\text {V}$ and at a certain temperature). We found that a term that is proportional to density is needed to fit the “higher” density regime, while another term that is inversely proportional to density is needed to fit the “lower” density regime. Subsequently, the dependence of the proportionality constants is studied as a function of $N_\text {V}$ and *T*. We found that the following parameterization of the volume model is sufficient to capture the corresponding physics:

8a $$\begin{aligned} \frac{V}{\Omega N_\text {V}}= \,\,& {} 1 - a_1 N_\text {V}^{-a_2} + b_1 N_\text {V}^{-b_2} \eta - c_1 N_\text {V}^{-c_2}/\eta \end{aligned}$$

8b $$\begin{aligned} \eta= \,\,& {} N_\text {He}/N_\text {V}\ \end{aligned}$$

8c $$\begin{aligned} a_1= \,\,& {} a_{10} + a_{11} T_\mathrm{r} \end{aligned}$$

8d $$\begin{aligned} a_2= \,\,& {} a_{20} + a_{21} T_\mathrm{r} \end{aligned}$$

8e $$\begin{aligned} b_1= \,\,& {} b_{10} + b_{11} T_\mathrm{r} \end{aligned}$$

8f $$\begin{aligned} b_2= \,\,& {} b_{20} + b_{21} T_\mathrm{r} \end{aligned}$$

8g $$\begin{aligned} c_1= \,\,& {} c_{10} + c_{11} T_\mathrm{r} \end{aligned}$$

8h $$\begin{aligned} c_2= \,\,& {} c_{20} + c_{21} T_\mathrm{r} \end{aligned}$$

In the above model, the volume ratio ($V/(N_\text {V}\Omega )$) correctly approaches unity as $N_\text {V}$ approaches $\infty$. The third term adds a positive correction in the high density regime, while the fourth term contributes a negative correction in the low density regime. Finally, the optimally fitted parameter values are given in Table 4.

**Table 4:** Fitted parameters $a_{1i}$, $a_{2i}$, $b_{1i}$, $b_{2i}$, $c_{1i}$, and $c_{2i}$ in the volume model of He bubbles in W as formulated in Eq. (8).

*i*	$a_{1i}$	$a_{2i}$	$b_{1i}$	$b_{2i}$	$c_{1i}$	$c_{2i}$
0	0.5572	0.1543	0.4342	0.2027	0.2086	0.3414
1	$5.511\times 10^{-4}$	$3.526\times 10^{-5}$	$1.947\times 10^{-4}$	$-1.328\times 10^{-4}$	$-1.087\times 10^{-4}$	$-9.824\times 10^{-6}$

**Figure 17:**
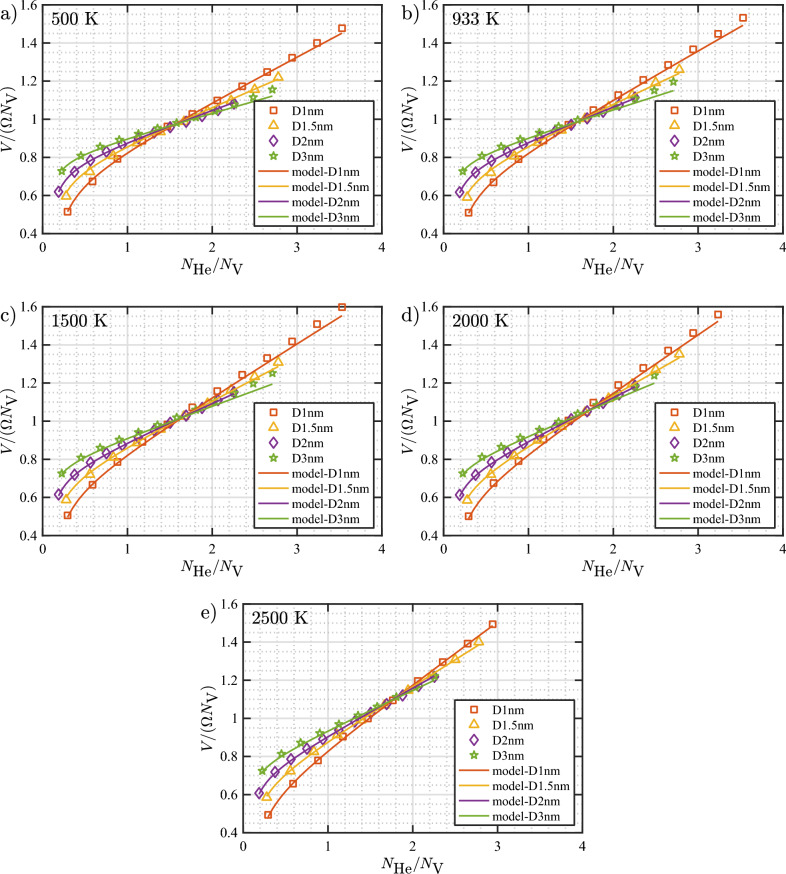
Plots of the ratio of bubble volume (*V*) to $N_\text {V}\Omega$ as a function of $N_\text {He}/N_\text {V}$ for He bubbles in bulk W. Data points are colored based on the number of vacancies (see Table 3 for more information). Data points are from MD simulations, while the solid lines represent calculation using the volume model derived in Eq. (8).

Figure 17 shows how the volume ratio varies as a function of $N_\text {He}/N_\text {V}$ for a given $N_\text {V}$ and *T*. In Fig. 17, MD data are plotted together with the corresponding curves calculated with the volume model. At 500 K, the volume ratio is approximately equal to 1 in the $N_\text {He}/N_\text {V}$ range of 1.6–1.8, depending on the bubble size ($N_\text {V}$). This range gradually decreases to 1.2–1.6 at 2500 K. It can be clearly seen that above this range (the “higher” $N_\text {He}/N_\text {V}$ regime), the volume ratio becomes increasingly larger than 1, while the opposite is true towards the “lower” $N_\text {He}/N_\text {V}$ regime.

In the bubbles labelled as D1nm, D1.5nm, D2nm, and D3nm, there are 34, 108, 266, and 886 vacancies, respectively. Looking back at the loop punching data of subsurface bubbles (Fig. 9), at 933 K, the $N_\text {He}/N_\text {V}$ ratio at the loop punching events for bubbles containing the above values of vacancies is 4.1, 3.4, 3.1, and 2.7, respectively, as calculated with the loop punching model. At these values of $N_\text {He}/N_\text {V}$, the bubble volume (as calculated with the volume model) is 1.64, 1.38, 1.26, and 1.15 times larger than $N_\text {V}\Omega$. At 2500 K, the corresponding ratios are 1.56, 1.37, 1.28, and 1.19, revealing a small dependence on temperature. This analysis shows a significant deviation if one were to use $N_\text {V}\Omega$ as the volume for the overpressurized subsurface bubbles.

### Pressure in subsurface He bubbles

**Figure 18:**
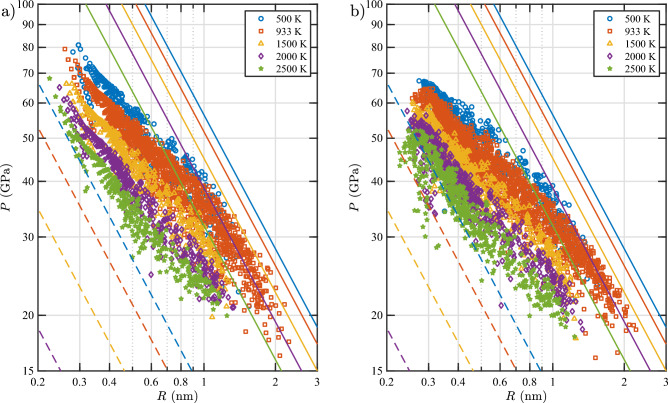
Loop punching pressure, *P*, of subsurface He bubbles in W as a function of bubble radius, *R*. In (**a**), the pressure is calculated using the bubble EOS (Eqs. 6 and 7) where the bubble volume is calculated according to the volume model (Eq. 8). In (**b**), the pressure and volume are obtained directly from MD simulations. Solid and dashed lines represent the theoretical loop-punching pressures ($P = (2\gamma +Gb)/R$) and equilibrium pressures ($P = 2\gamma /R$), respectively, for spherical bubbles in bulk W as derived in Ref.^28^. The theoretical equilibrium line at 2500 K is outside the view area.

In this section we present the pressure in the subsurface bubbles at the loop punching events as calculated using the newly derived bubble EOS and the volume model. The inputs for these calculations are $N_\text {V}$, $N_\text {He}/N_\text {V}$, and *T*. Figure 18a shows the results, where the pressure is plotted as a function of the bubble radius *R* (where *R* is calculated from the bubble volume, assuming a spherical bubble). Plots for bubbles over the temperature range from 500 to 2500 K are presented. For all bubbles, the pressure exceeds 15 GPa. The majority of the bubbles exhibit a loop punching pressure above 20 GPa. Comparing the data at various temperatures, it is evident that the loop punching pressure is lower at higher temperature, which can be understood as a consequence of material softening.

For bubbles in a bulk material, a commonly used theoretical model for loop punching pressure is $P = (2\gamma + Gb)/R$, where $\gamma$ is the free energy per unit area of the internal surface of the bubble cavity, *G* is the shear modulus of the material (here W), *b* is the magnitude of the Burgers vector of the <111> interstitial loop (to be punched out), and *R* is the bubble radius. The values of $\gamma$, *G*, and *b* as a function of temperature can be found in^28^. The theoretical curve of loop punching pressure for bulk bubbles is superimposed onto the data in Fig. 18a. Apparently, the loop punching pressure of the subsurface bubbles is below the theoretical line of bulk bubbles. A free surface attracts an interstitial loop, lowering the pressure to cause a loop punching event. Computational studies on a similar interaction between He clusters and free surfaces in W can be found in^30–32^.

To compare the loop punching pressure of subsurface bubbles calculated with the EOS with that directly obtained from the MD simulations, we plot the pressure from MD simulations in Fig. 18b. It can be seen that the pressure calculated with the bubble EOS (along with the bubble volume model prediction) compares very well with the MD pressure. Therefore, we conclude that a consistent set of models for loop punching, bubble bursting, bubble EOS, and bubble volume has been successfully developed in this study.

## Conclusions

We have derived a set of empirical models fitted to MD data to describe the conditions that cause loop punching and bursting events in over-pressurized He bubbles in the subsurface region of He-implanted W. Data at 500, 933, 1500, 2000, and 2500 K were generated to fit the parameters in the models. The models are intended to inform mesoscale simulations of He accumulation and damage evolution on plasma-exposed tungsten surfaces. Since such mesoscale simulations often store the information about bubbles in terms of the number of vacancies ($N_\text {V}$), the number of He atoms ($N_\text {He}$), and temperature (*T*), the models are conveniently formulated in these terms. Specifically, in the loop punching model, the $N_\text {He}/N_\text {V}$ ratio that causes the event, the resulting change in $N_\text {V}$  and the associated shift of the bubble depth (towards the surface) are expressed as a function of $N_\text {V}$ and *T*. In the bursting model, for a given $N_\text {V}$ and *T*, a bubble must reach a certain depth and $N_\text {He}/N_\text {V}$ ratio simultaneously in order to burst. To compute the pressure in the bubbles, an EOS for He bubbles in W that accounts for the interaction between He and W atoms has been derived along with a model needed to calculate the volume of the bubbles as a function of $N_\text {V}$, $N_\text {He}/N_\text {V}$ ratio, and *T*. The pressures of subsurface bubbles at the loop punching events as calculated with the bubble-EOS agree very well with the pressures obtained directly from the MD simulations. The majority of the loop punching events occur in the pressure range of 20 to 60 GPa, depending on the radius of the bubbles, the larger the radius the lower the pressure. The loop punching and bursting pressures were observed to decrease at higher temperatures due to material softening. Furthermore, the models reveal that for a given size, a deeper bubble can burst at a higher temperature.

In addition, a new EOS for a free He gas has been developed to improve the existing free-gas EOS that was developed by Mills, Liebenberg, and Bronson (EOS-MLB). The EOS-MLB was fitted to experimental data of a He gas in the pressure range of 0.2–2 GPa and temperature range of 75–300 K. It was found that the EOS-MLB agrees very well with MD data up to about 500 K. However, it over-predicts the pressure at temperatures of 933 K and above, and becomes increasingly inaccurate at higher temperatures and gas densities. The new free-gas EOS is accurate in predicting all the MD data included in the fitting procedure (the highest pressure is 54 GPa at the highest temperature of 2500 K).

## Supplementary Information

Supplementary Information 1.

## Data Availability

The datasets used and/or analysed during the current study available from the corresponding author on reasonable request.
