# Supplementary material for: Equation of state for He bubbles in W and model of He bubble growth and bursting near W{100} surfaces derived from molecular dynamics simulations
Source: Sci Rep. 2023 Jun 13;13:9601. doi: 10.1038/s41598-023-35803-3 (PMC10264409; doi:10.1038/s41598-023-35803-3)
Supplement: Supplementary file 1 — Supplementary Information 1. [file 41598_2023_35803_MOESM1_ESM.pdf]

Supplementary Information

Equation of State for He Bubbles in W and Model of He Bubble Growth and Bursting Near W{100} Surfaces Derived from Molecular Dynamics Simulations

Wahyu Setyawan[1,*], Dwaipayan Dasgupta[2], Sophie Blondel[2], Giridhar Nandipati[1], Karl D. Hammond[3], Dimitrios Maroudas[4], Brian D. Wirth[2,5]

[1] Pacific Northwest National Laboratory, Richland, WA 99352
[2] Department of Nuclear Engineering, University of Tennessee, Knoxville, TN 37996
[3] Department of Chemical Engineering, University of Missouri, Columbia, MO 65211
[4] Department of Chemical Engineering, University of Massachusetts, Amherst, MA 01003
[5] Fusion Energy Division, Oak Ridge National Laboratory, Oak Ridge, TN 37830

*Corresponding author: wahyu.Setyawan@pnnl.gov

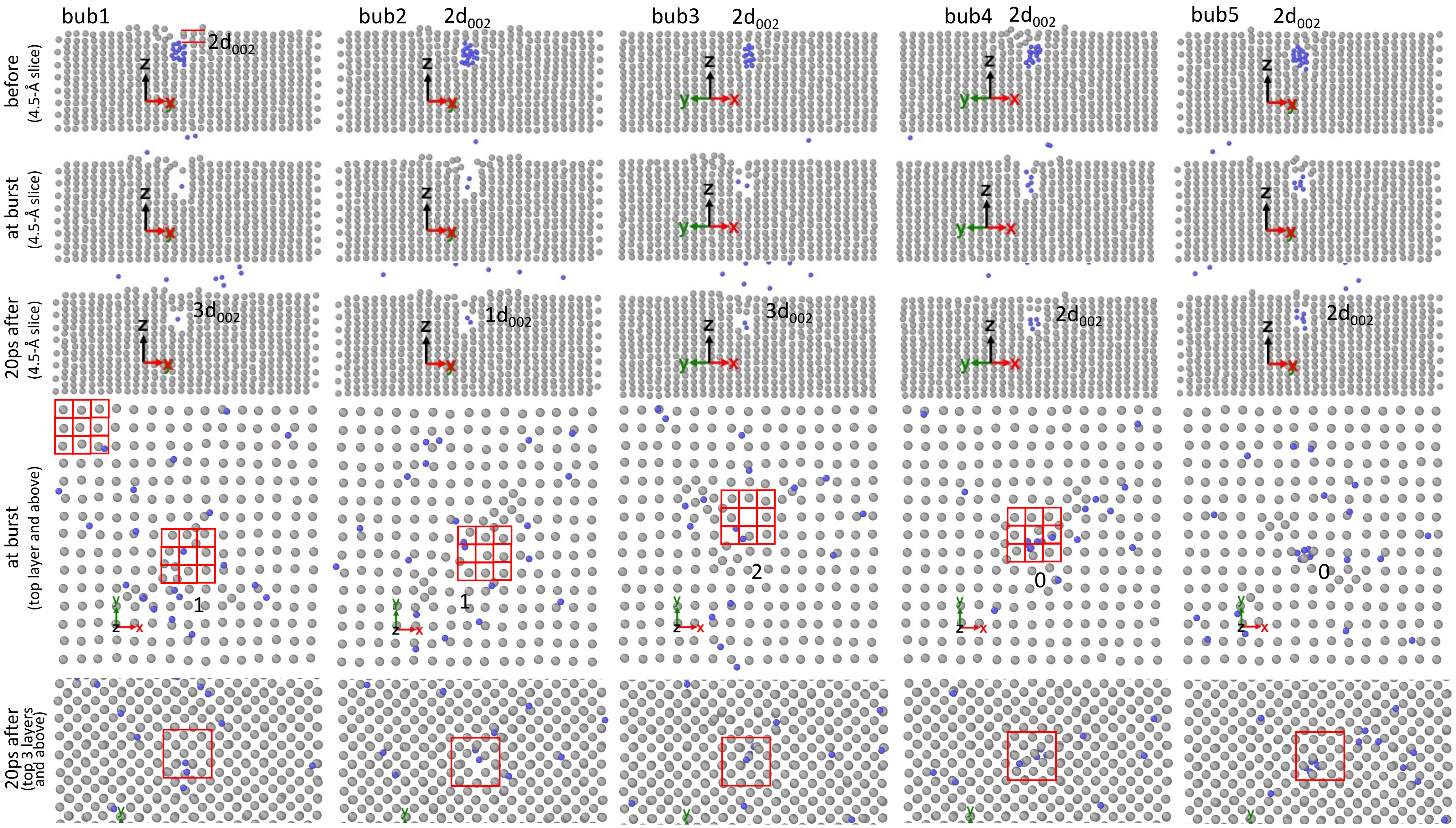

Supplementary Figure 1. Snapshots of bubbles initially nucleated at a depth of 5*a*/2, just before bursting ("before"), just after bursting ("at"), and at the end of the simulations (in this case, "20 ps after" bursting).

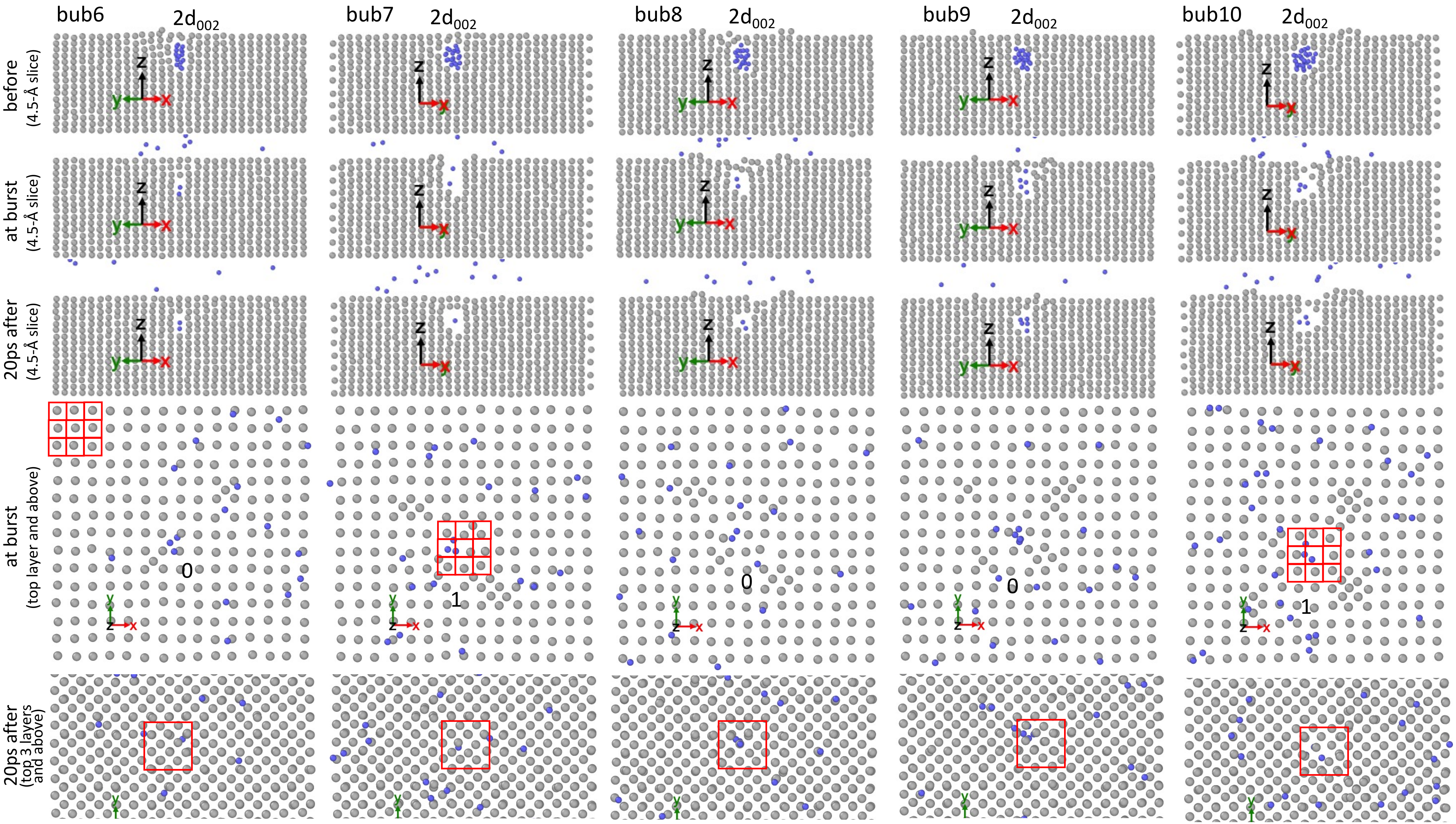

Supplementary Figure 1. Continued.

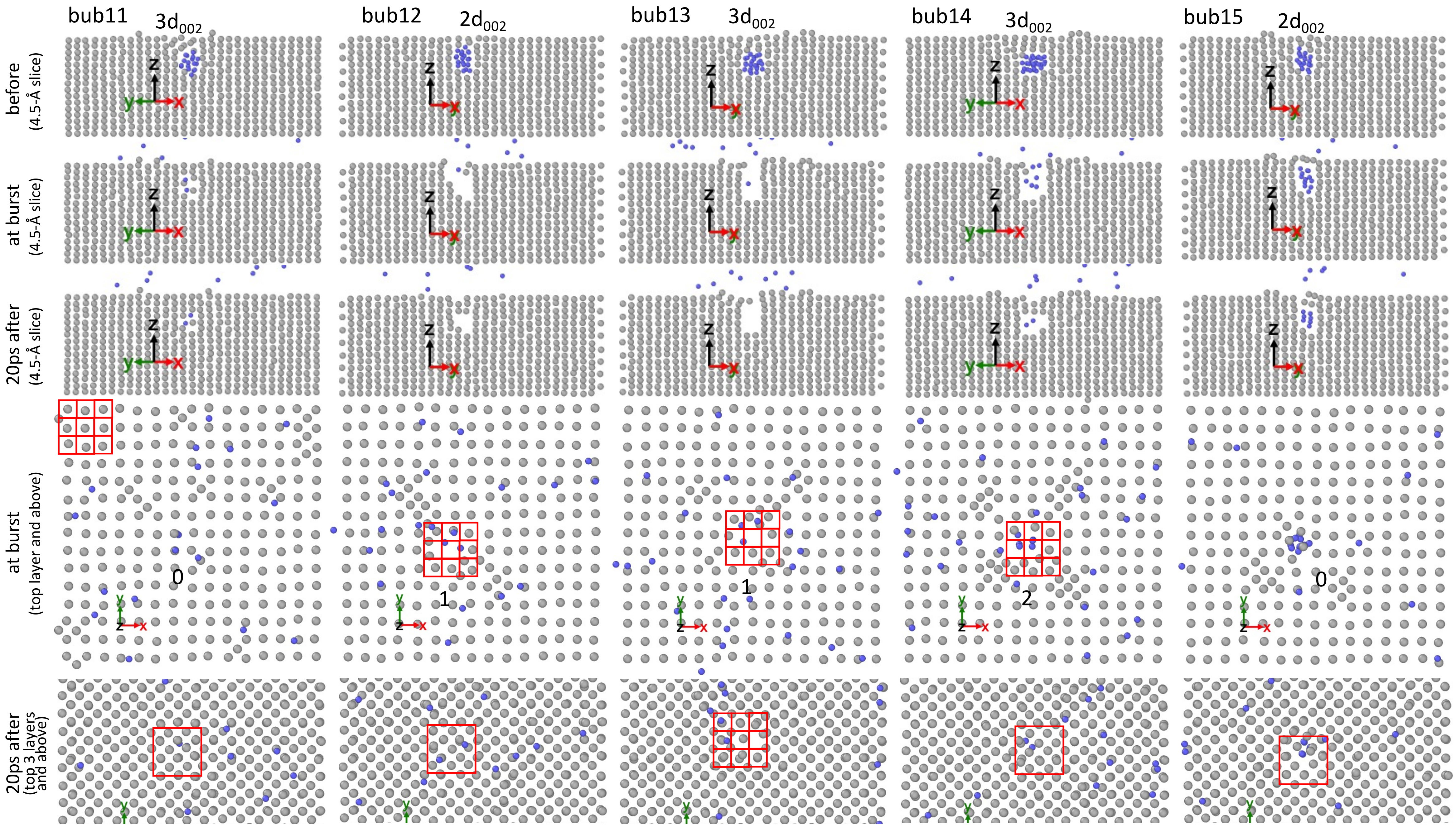

Supplementary Figure 1. Continued.

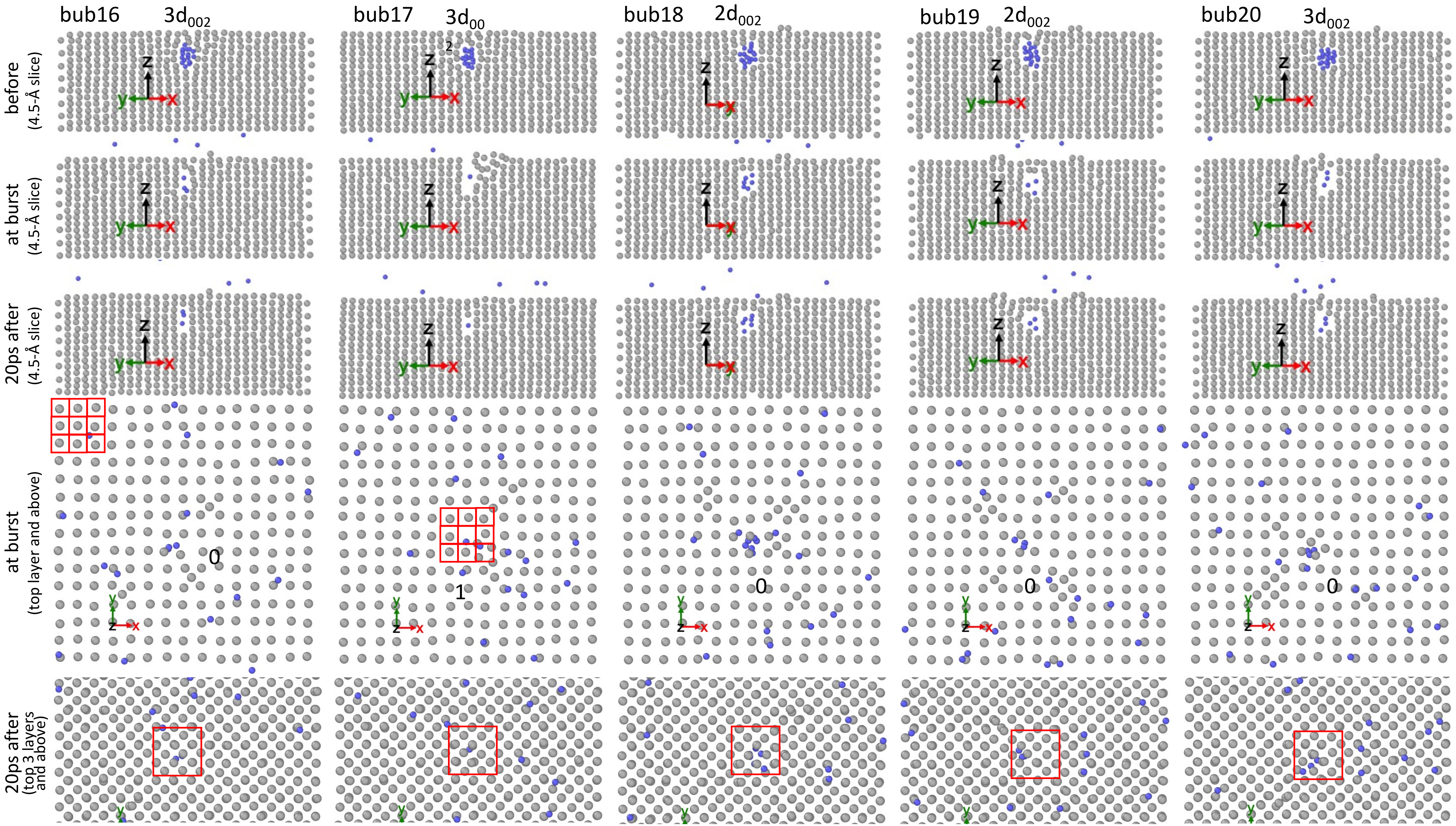

Supplementary Figure 1. Continued.

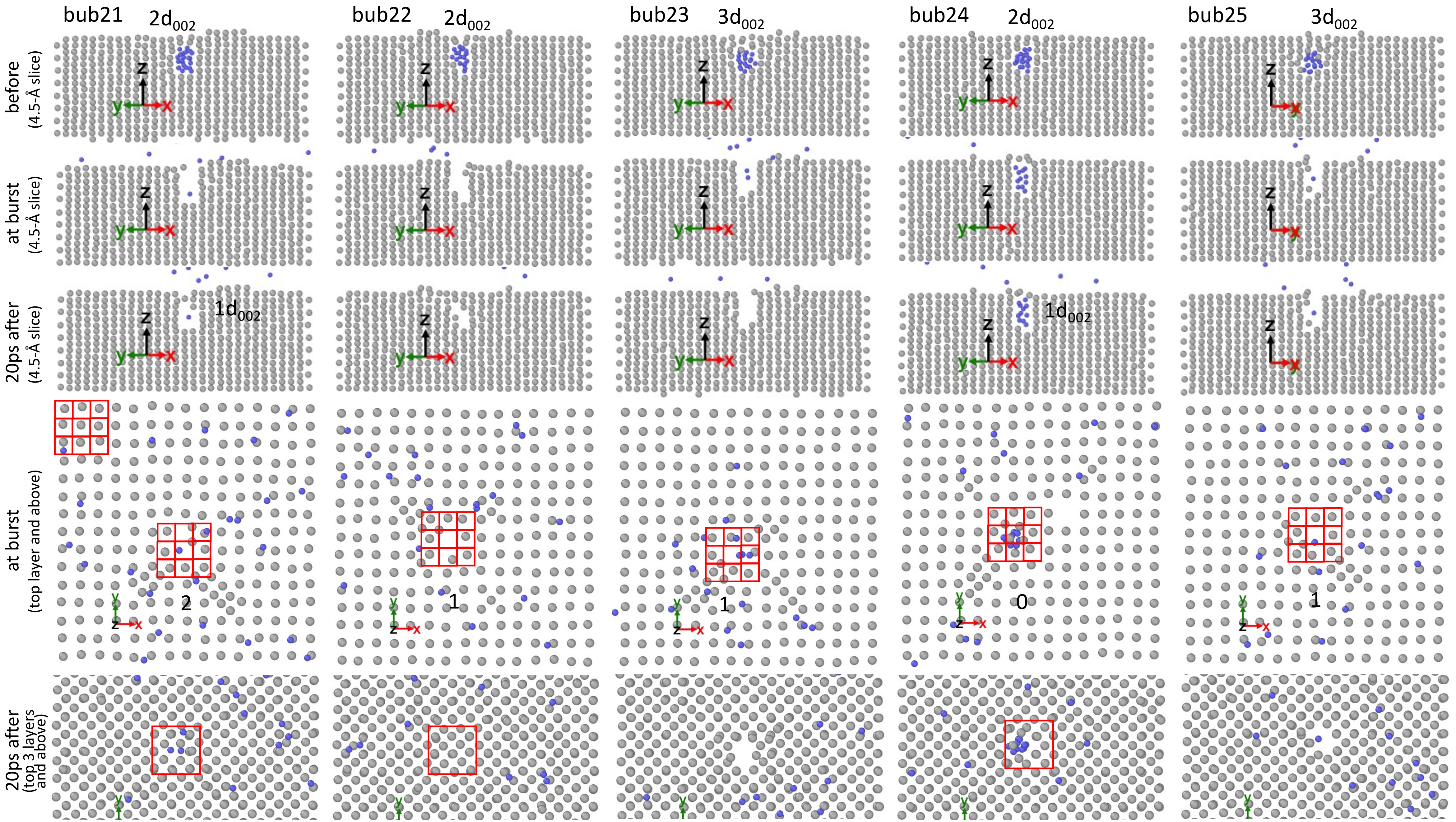

Supplementary Figure 1. Continued.

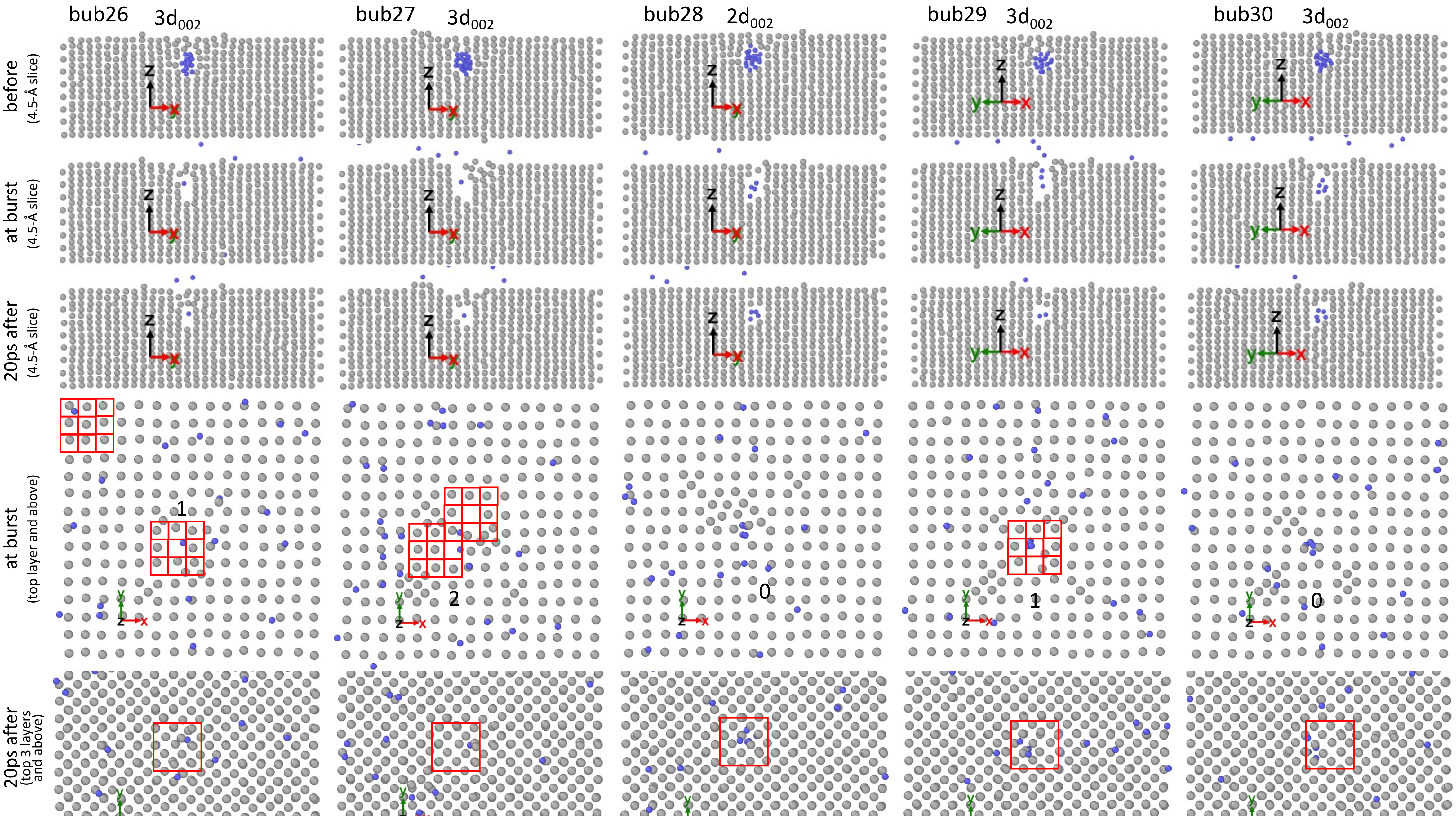

Supplementary Figure 1. Continued.

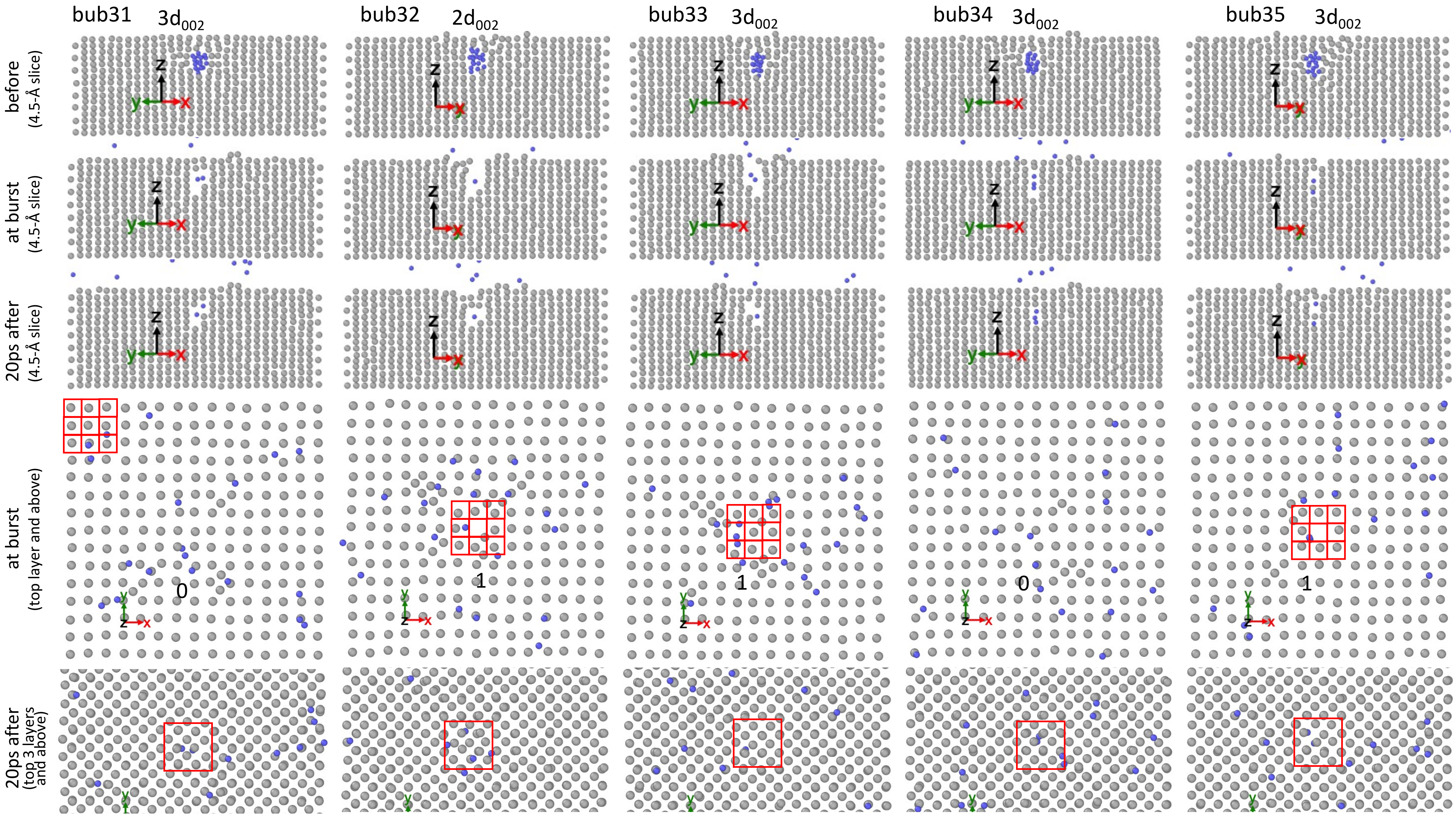

Supplementary Figure 1. Continued.

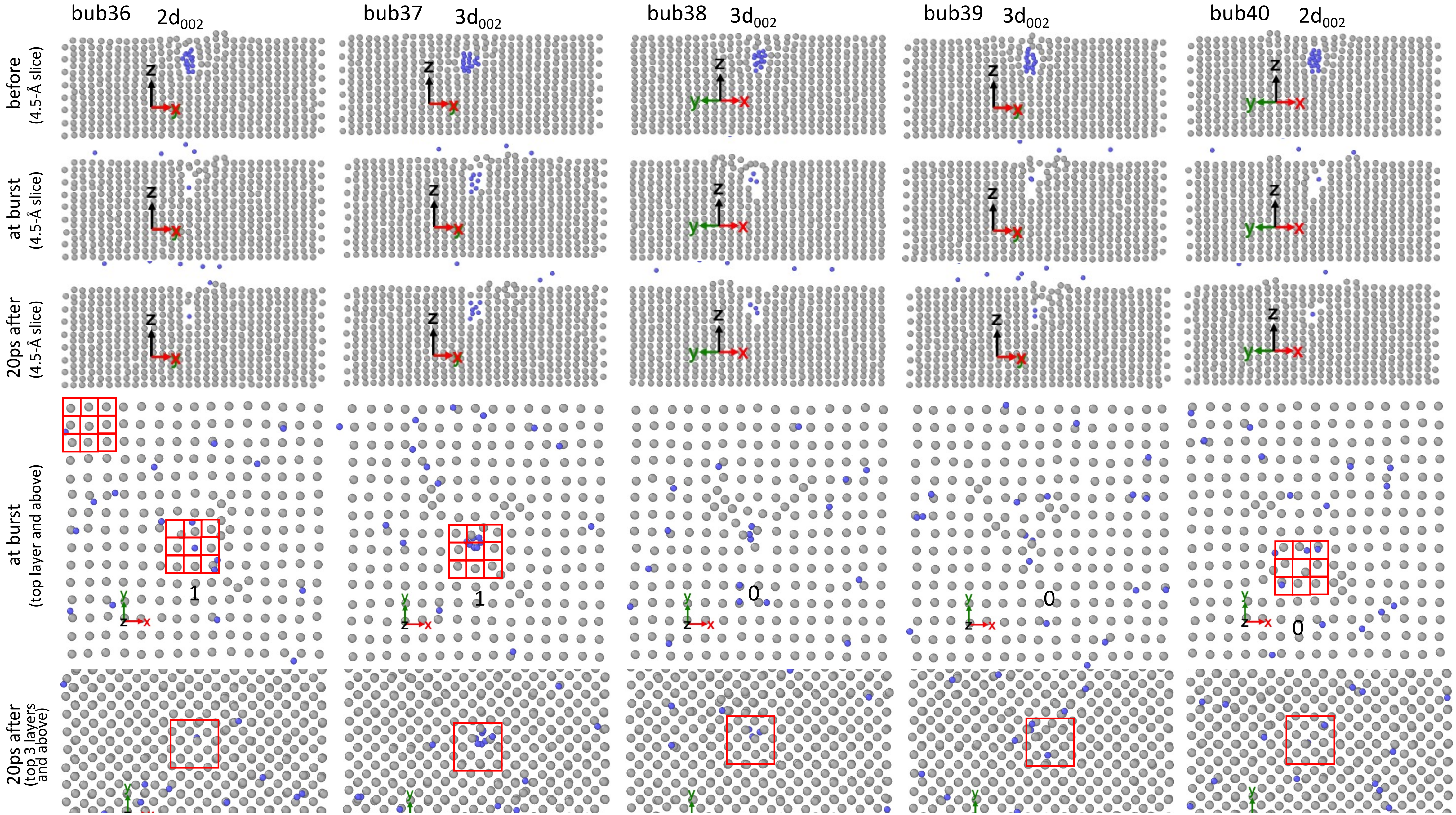

Supplementary Figure 1. Continued.

Supplementary Table 1. Data of thickness of W ligament above the bubble just before bursting (t_{lig}), area of burst hole (A_h), whether or not the bubble is resealed (1: yes, 0: no) after bursting, number of resealed layers, and the fraction of the number of He atoms left if case of resealed bubbles, form bubbles initially nucleated at depth of $5a/2$.

bub	t_{lig} (d_{002})	A_h (a^2)	reseal?	#resealed layers	f_{He}
1	2	1	1	3	0.053
2	2	1	1	1	0.100
3	2	2	1	3	0.125
4	2	0	1	2	0.313
5	2	0	1	2	0.300
6	2	0	1	3	0.125
7	2	1	1	2	0.050
8	2	0	1	3	0.105
9	2	0	1	2	0.250
10	2	1	1	1	0.094
11	3	0	1	3	0.118
12	2	1	1	2	0.000
13	3	1	1	1	0.000
14	3	2	1	2	0.071
15	2	0	1	1	0.389
16	3	0	1	2	0.167
17	3	1	1	3	0.063
18	2	0	1	2	0.316
19	2	0	1	2	0.158
20	3	0	1	3	0.158
21	2	2	1	1	0.043
22	2	1	1	2	0.000
23	3	1	0		
24	2	0	1	1	0.550
25	3	1	0		
26	3	1	1	2	0.063
27	3	2	1	2	0.042
28	2	0	1	3	0.200
29	3	1	1	2	0.111
30	3	0	1	2	0.263
31	3	0	1	2	0.111
32	2	1	1	2	0.053
33	3	1	1	2	0.059
34	3	0	1	3	0.167
35	3	1	1	2	0.133
36	2	1	1	2	0.059
37	3	1	1	1	0.350
38	3	0	1	2	0.176
39	3	0	1	2	0.111
40	2	0	1	3	0.059
average	2.5	0.6	95%	2.1	14%

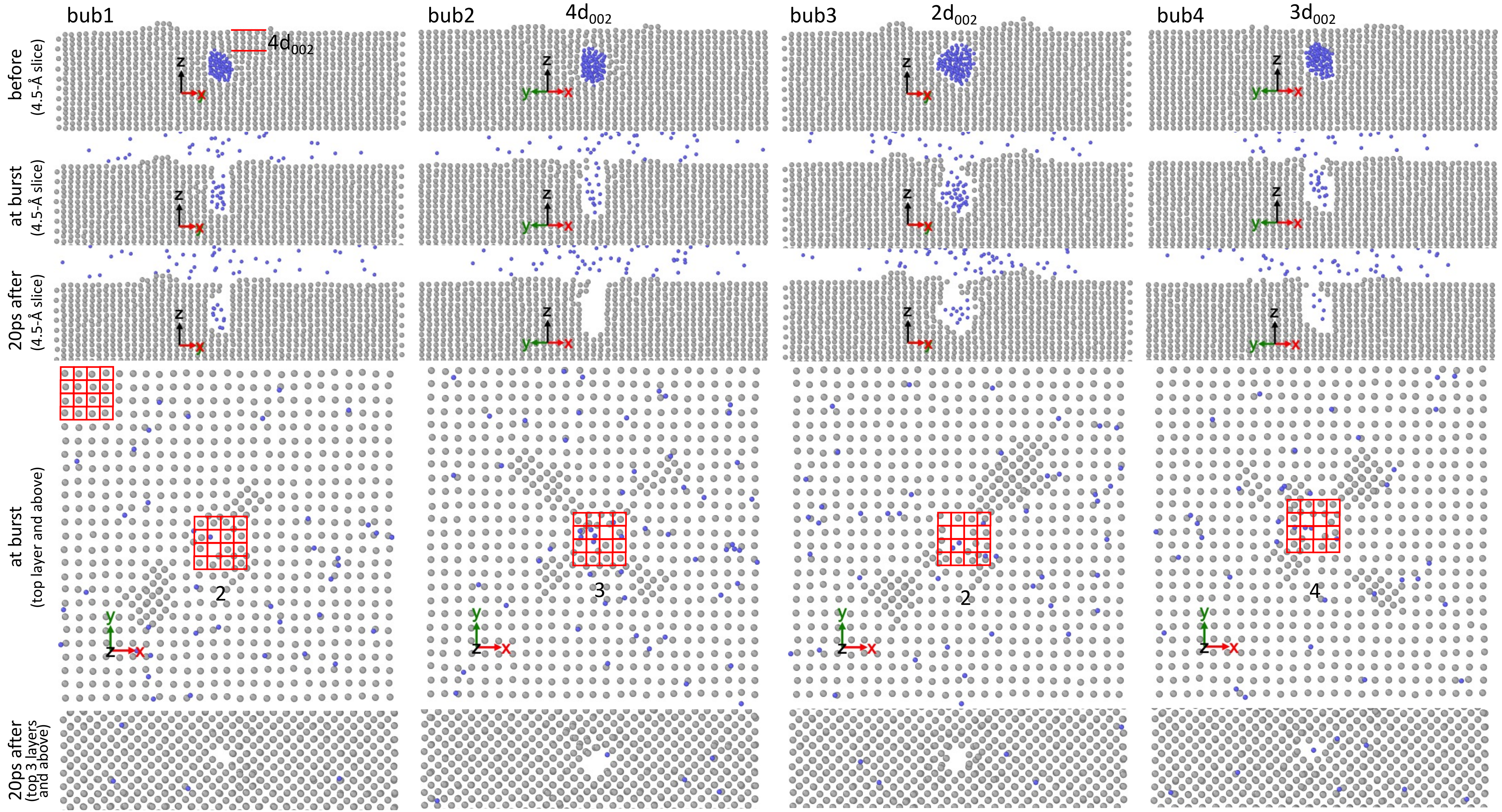

Supplementary Figure 2. Snapshots of bubbles initially nucleated at a depth of $9a/2$, just before bursting ("before"), just after bursting ("at"), and at the end of the simulations (in this case, "20 ps after" bursting).

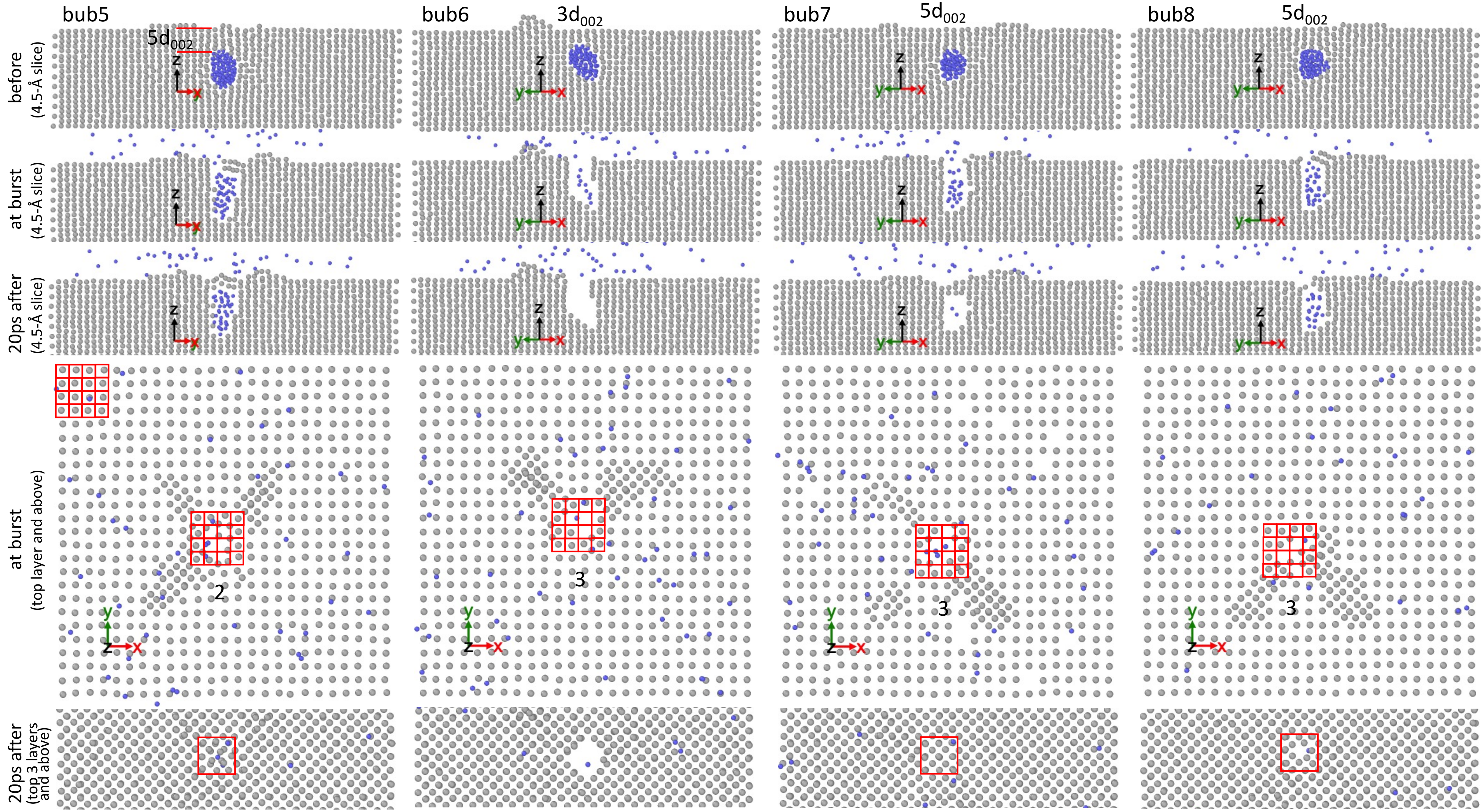

Supplementary Figure 2. Continued.

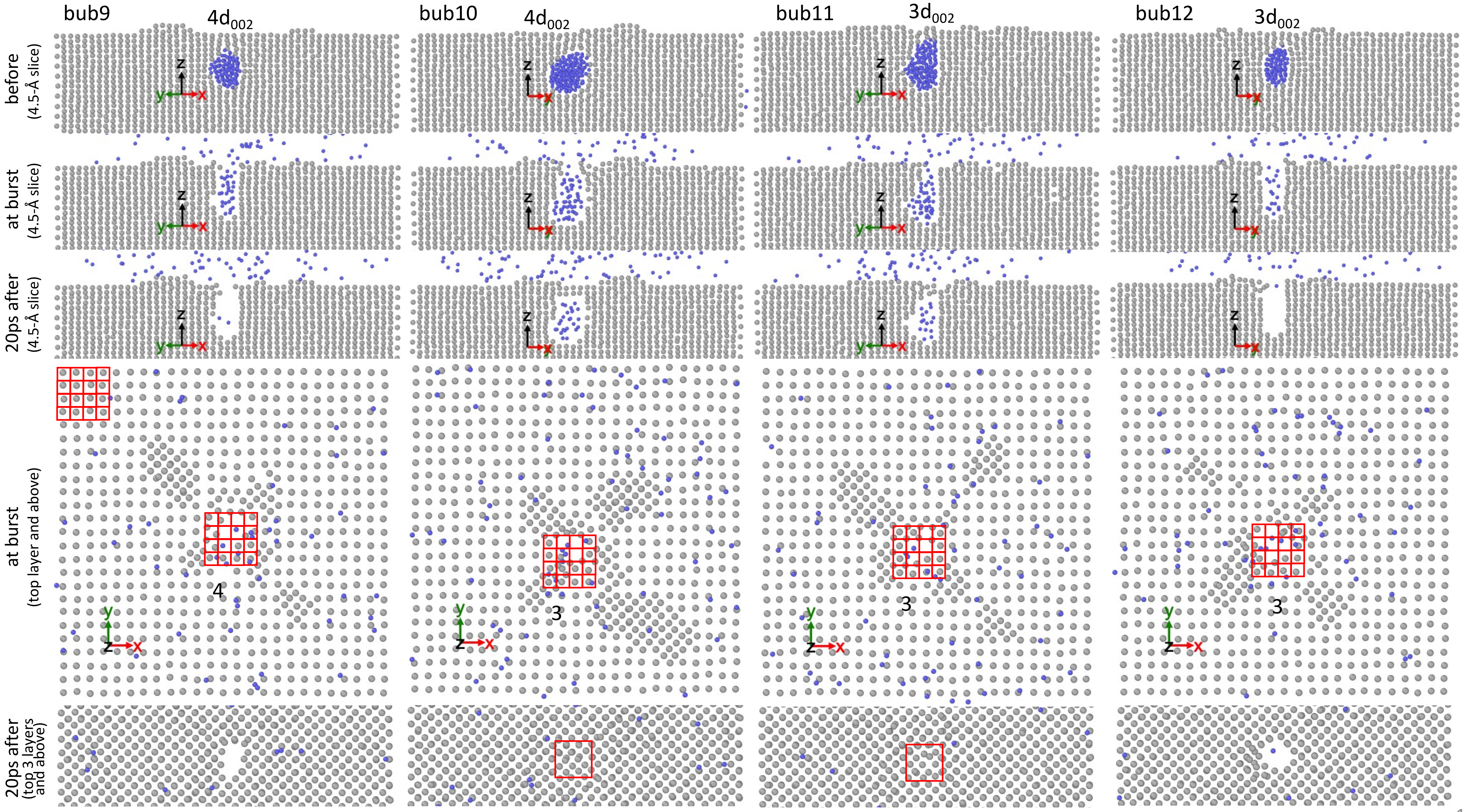

Supplementary Figure 2. Continued.

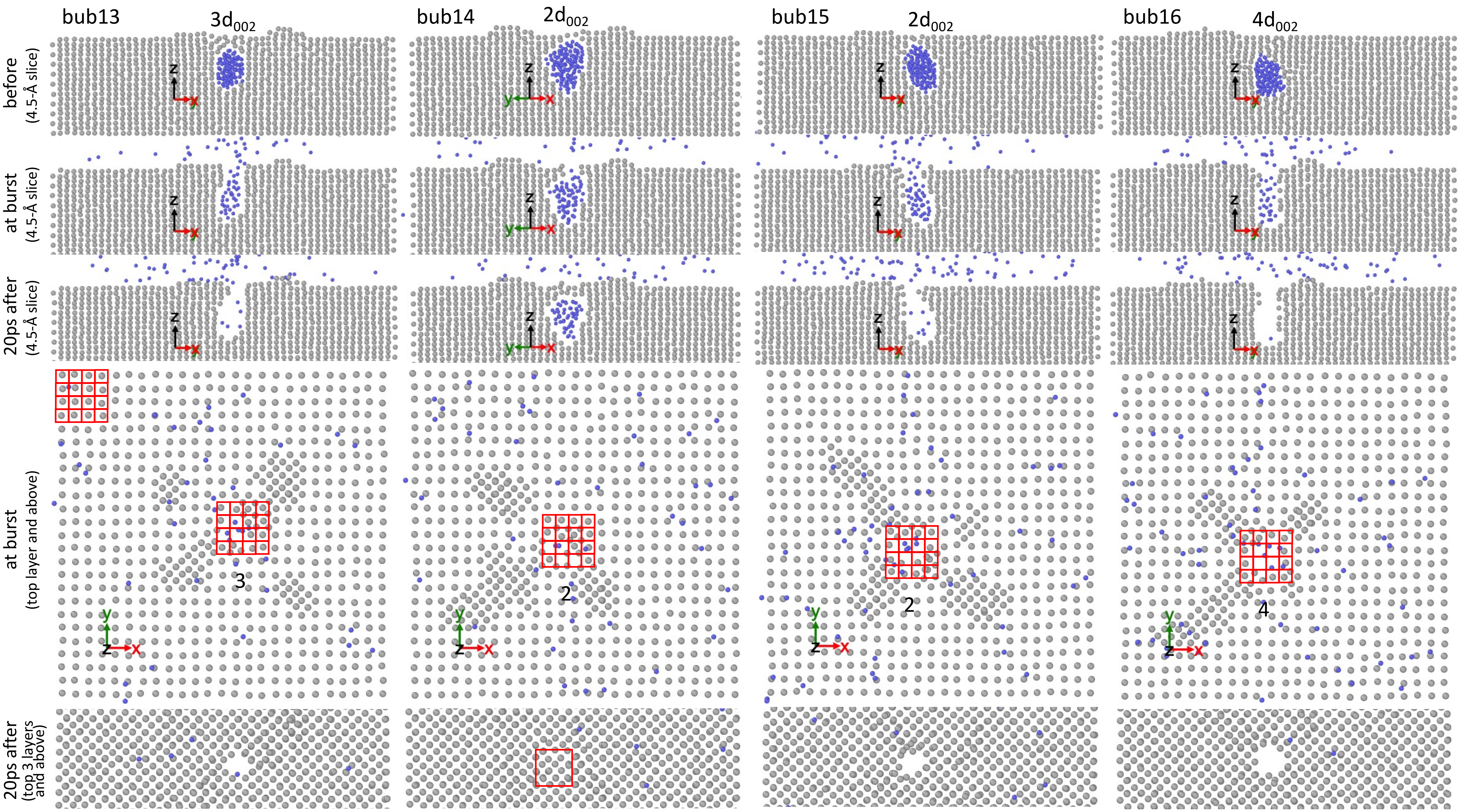

Supplementary Figure 2. Continued.

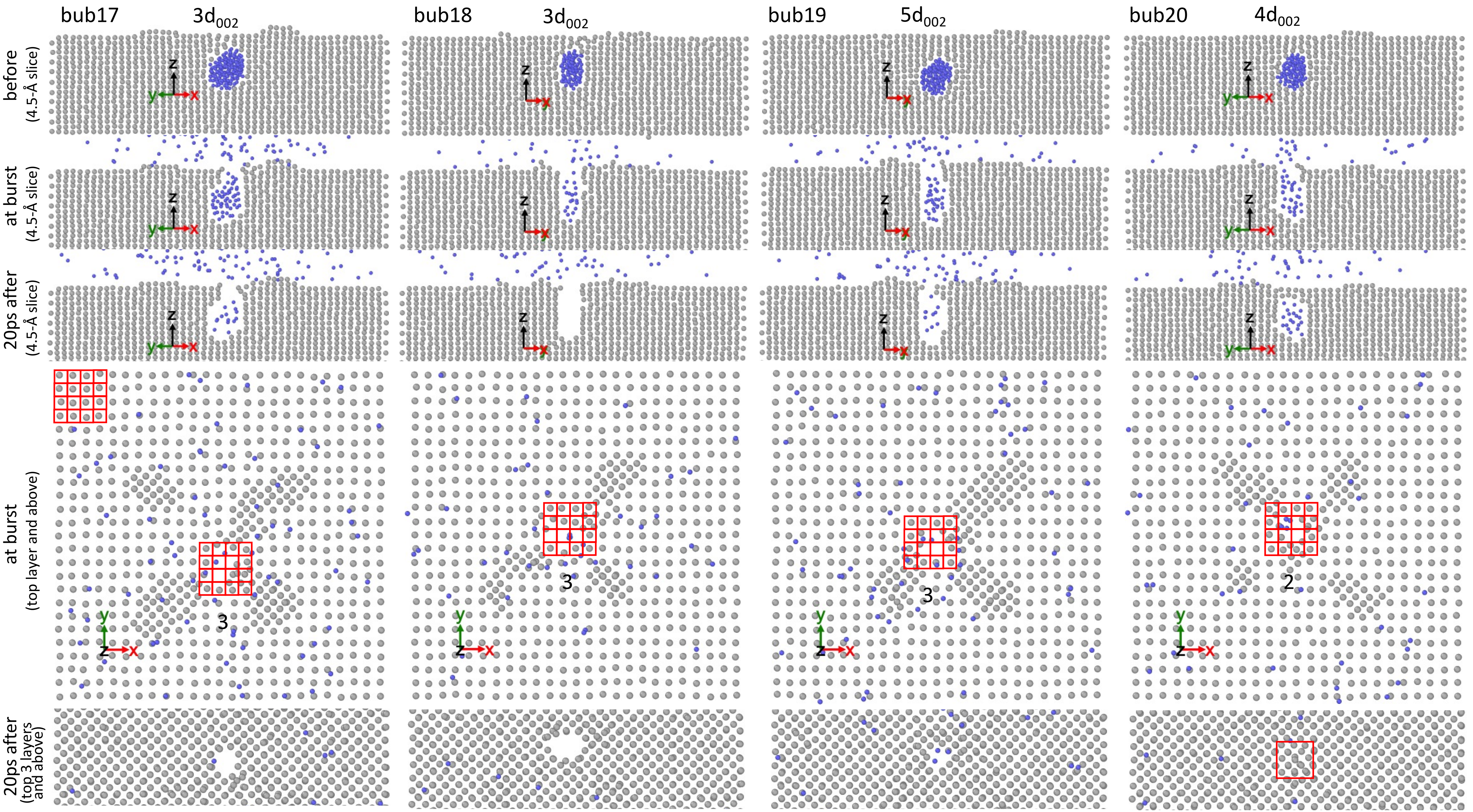

Supplementary Figure 2. Continued.

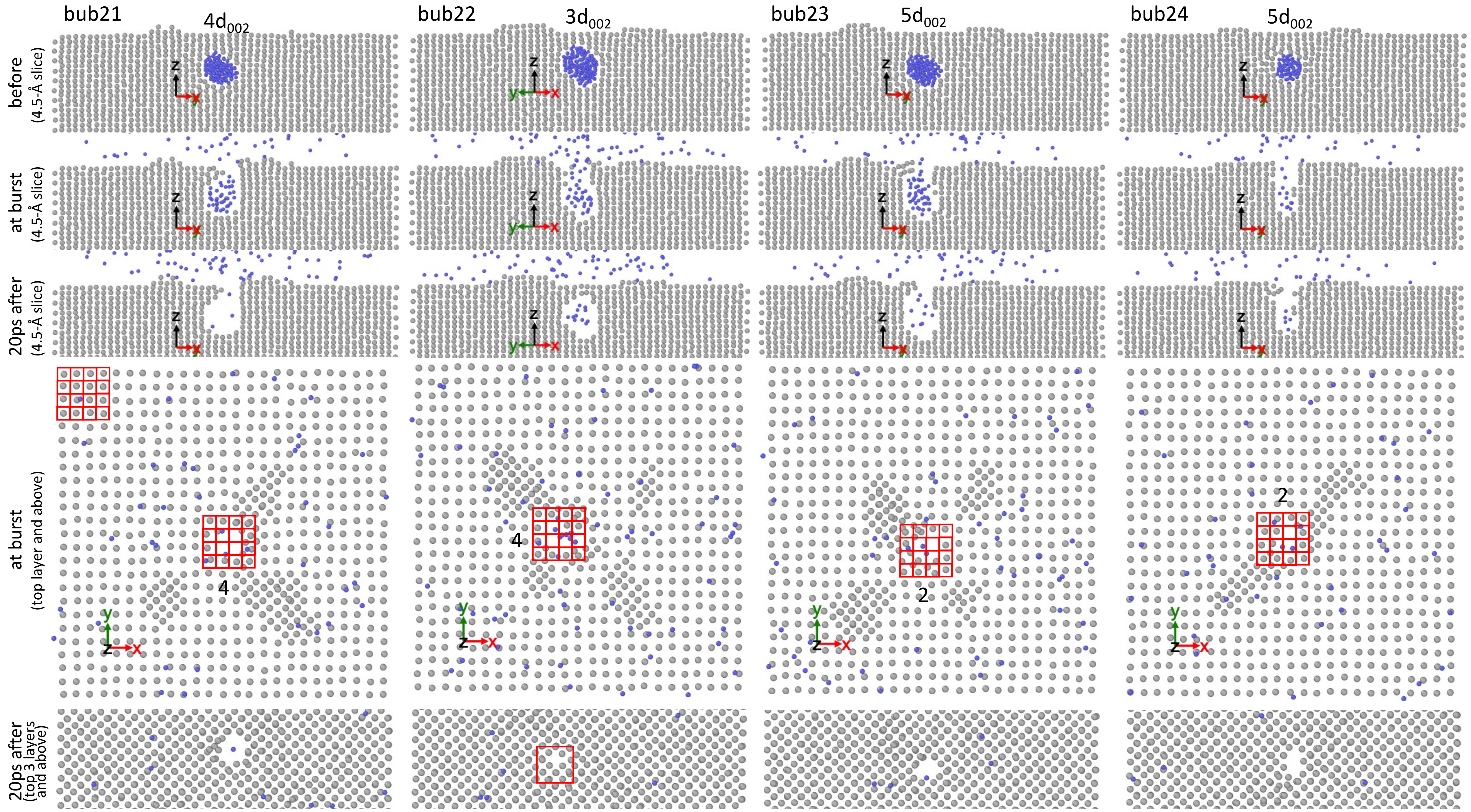

Supplementary Figure 2. Continued.

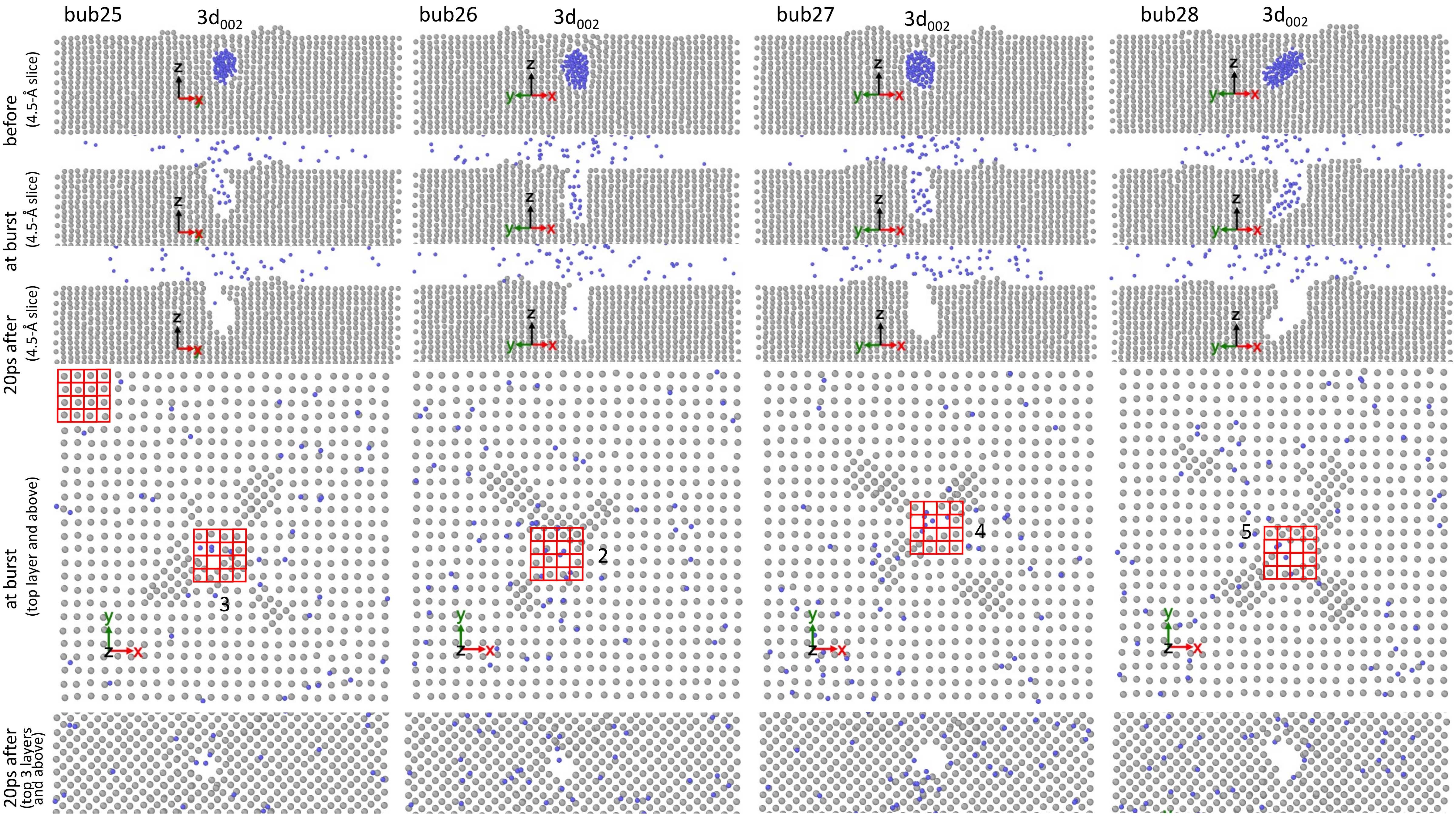

Supplementary Figure 2. Continued.

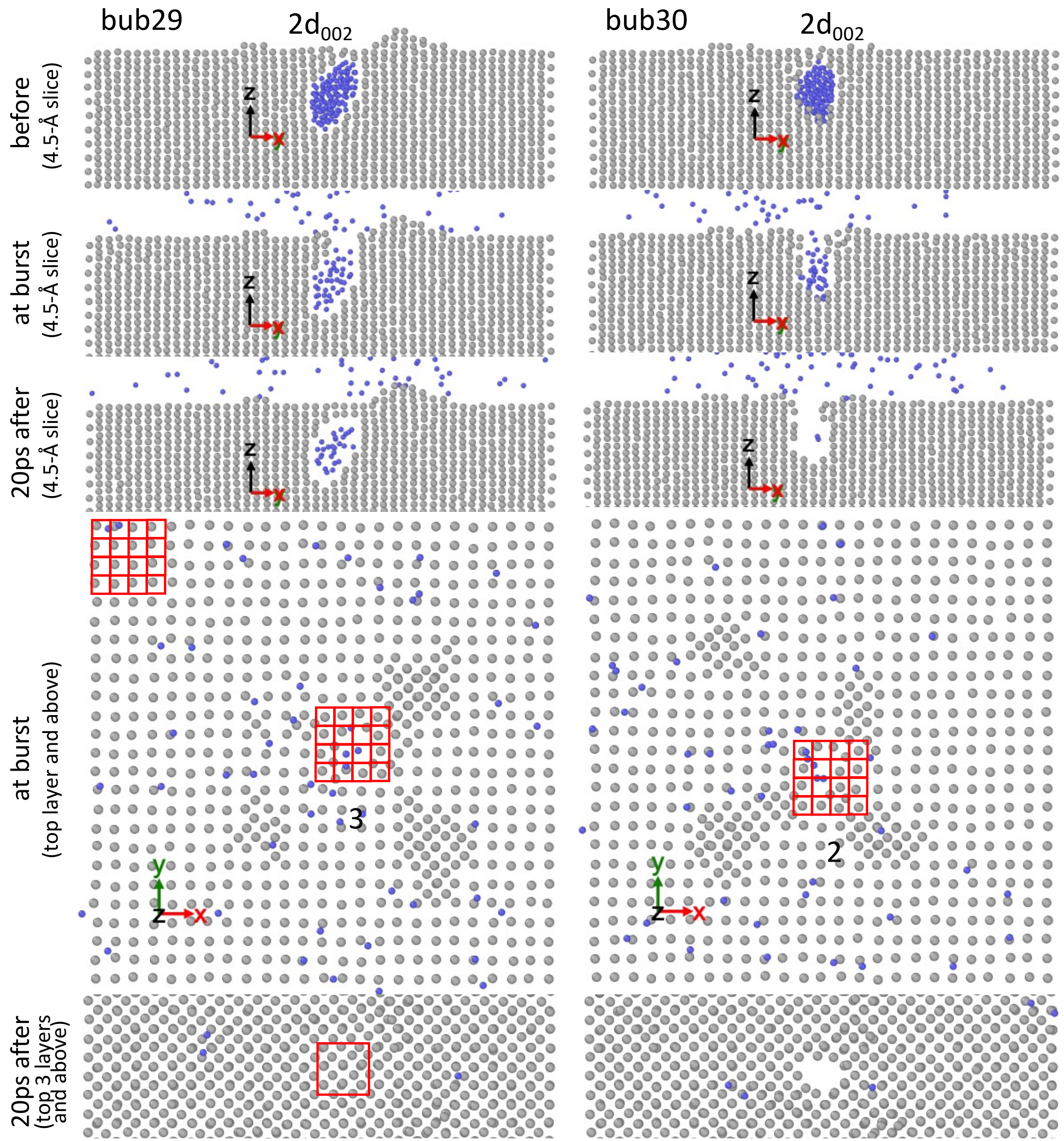

Supplementary Figure 2. Continued.

Supplementary Table 2. Data of thickness of W ligament above the bubble just before bursting (t_{lig}), area of burst hole (A_h), whether or not the bubble is resealed (1: yes, 0: no) after bursting, number of resealed layers, and the fraction of the number of He atoms left if case of resealed bubbles, form bubbles initially nucleated at depth of 9*a*/2.

bub	t_{lig} (d_{002})	A_h (a^2)	reseal?	#resealed layers	f_{He}
1	4	2	0		
2	4	3	0		
3	2	2	0		
4	3	4	0		
5	5	2	1	2	0.349
6	3	3	0		
7	5	3	1	2	0.023
8	5	2	1	1	0.250
9	4	4	0		
10	4	3	1	2	0.143
11	3	3	1	2	0.105
12	3	3	0		
13	3	3	0		
14	2	2	1	2	0.343
15	2	2	0		
16	4	4	0		
17	3	3	0		
18	3	3	0		
19	5	3	0		
20	4	2	1	2	0.186
21	4	4	0		
22	3	4	1	1	0.076
23	5	2	0		
24	5	2	0		
25	3	3	0		
26	3	2	0		
27	3	4	0		
28	3	5	0		
29	2	3	1	2	0.212
30	2	2	0		
average	3.5	2.9	30%	1.8	19%

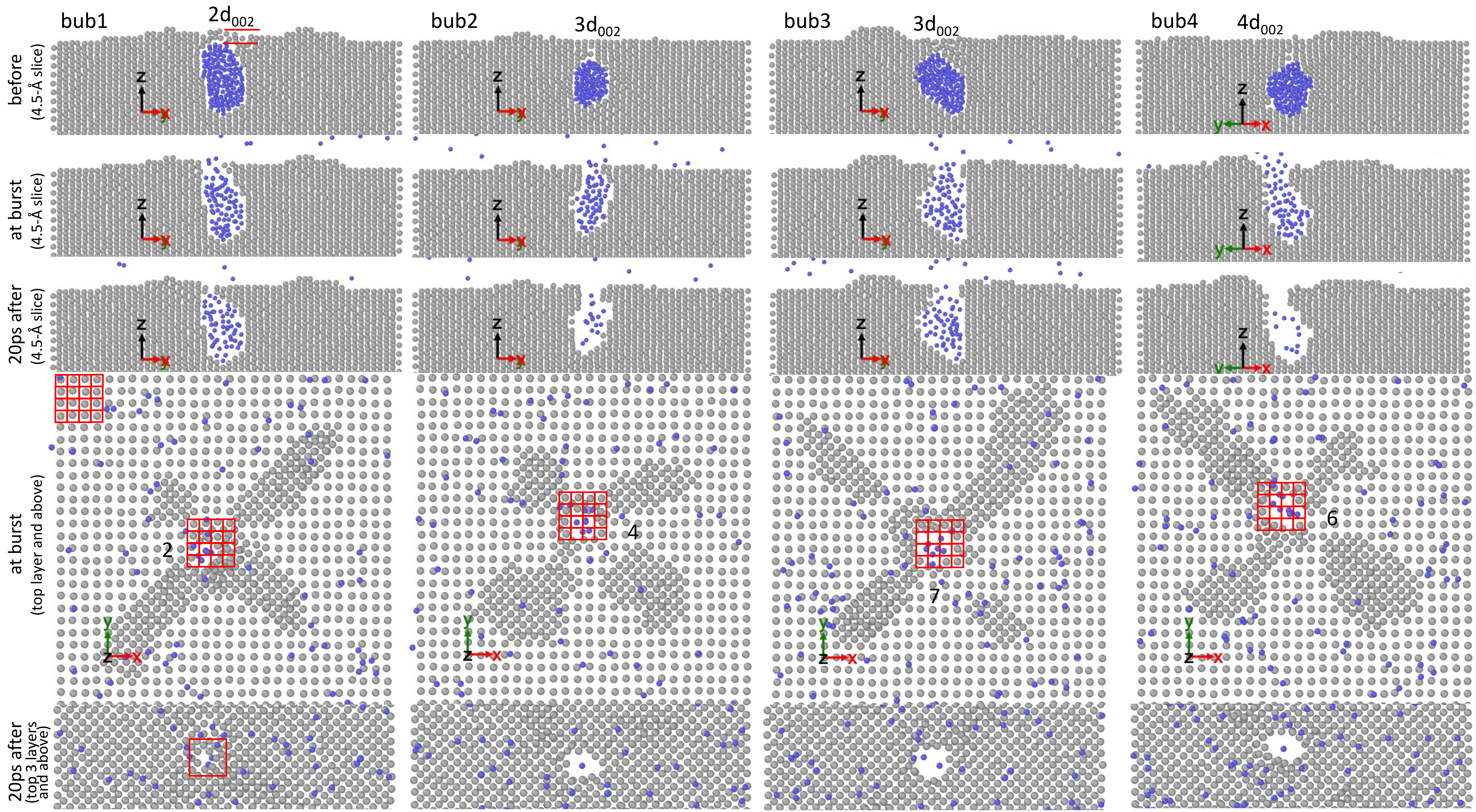

Supplementary Figure 3. Snapshots of bubbles initially nucleated at a depth of 13a/2, just before bursting (“before”), just after bursting (“at”), and at the end of the simulations (in this case, “20 ps after” bursting).

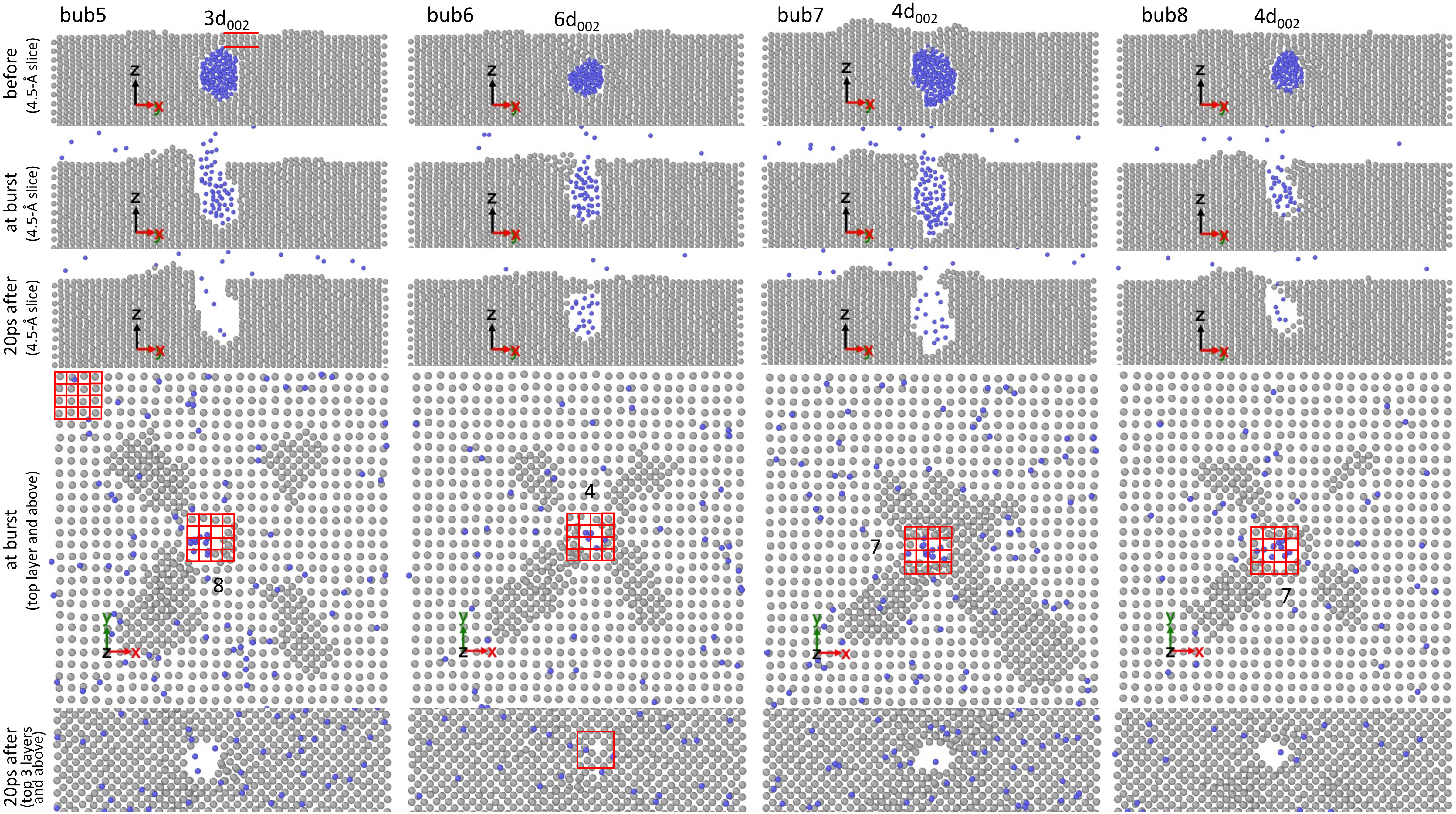

Supplementary Figure 3. Continued.

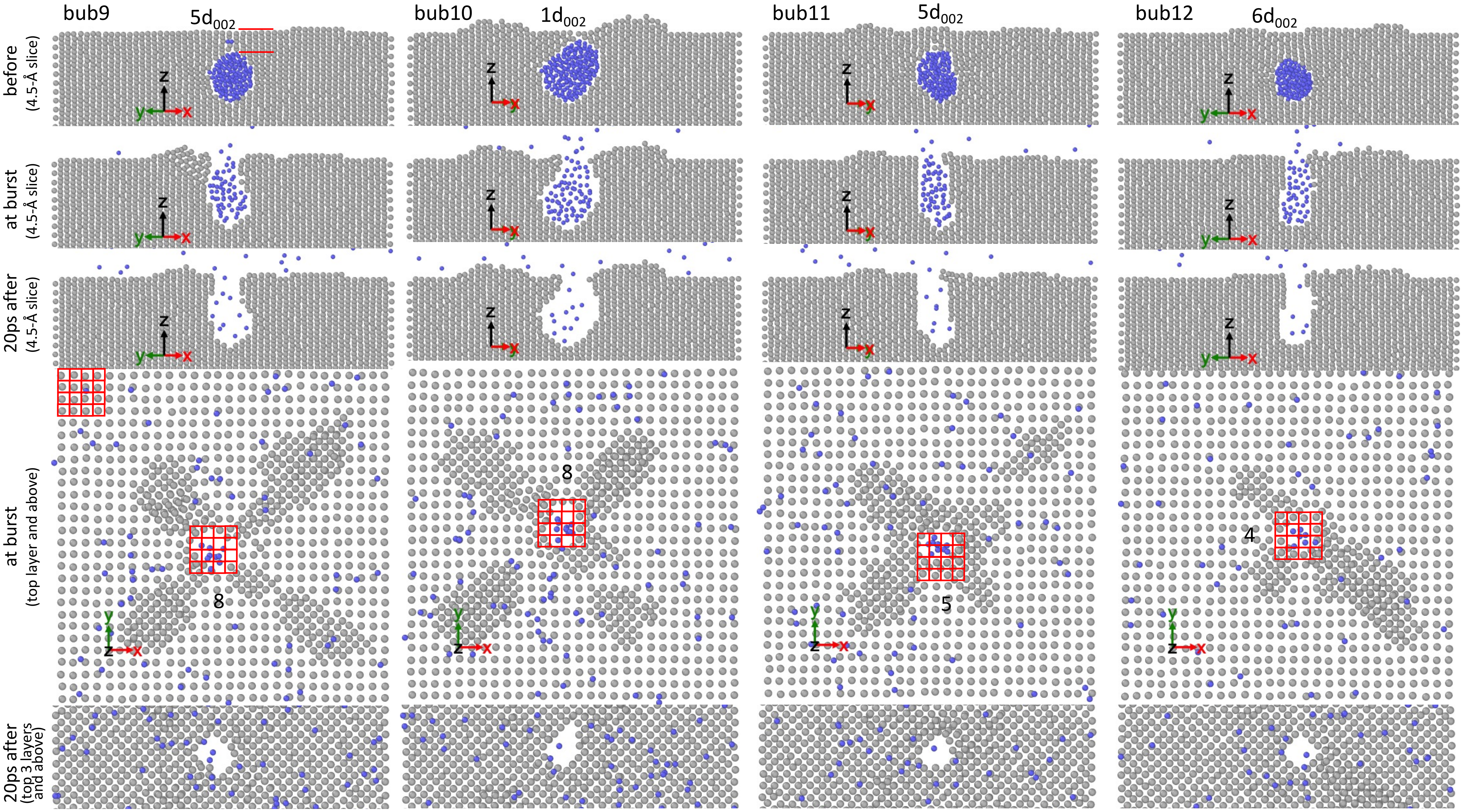

Supplementary Figure 3. Continued.

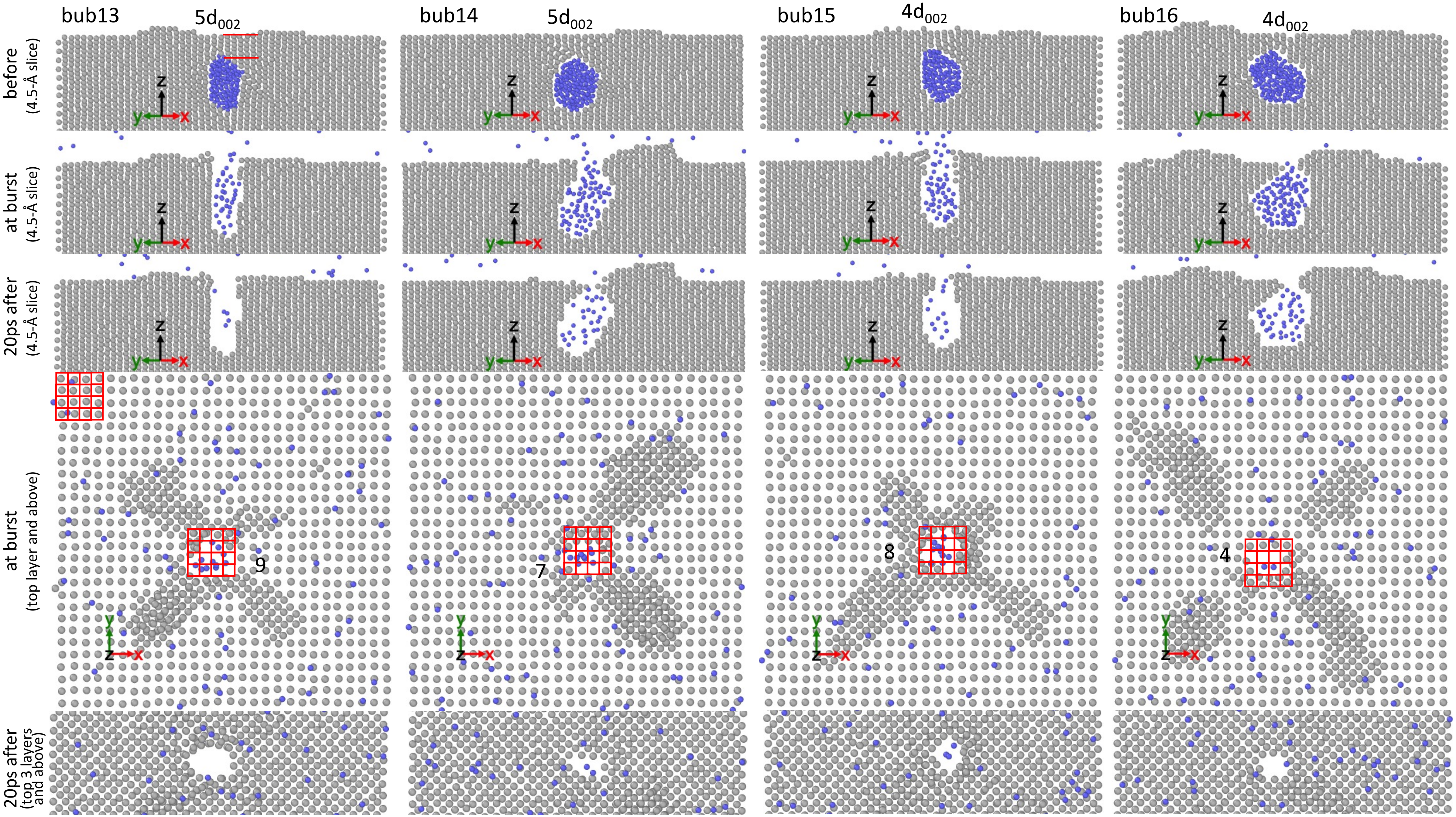

Supplementary Figure 3. Continued.

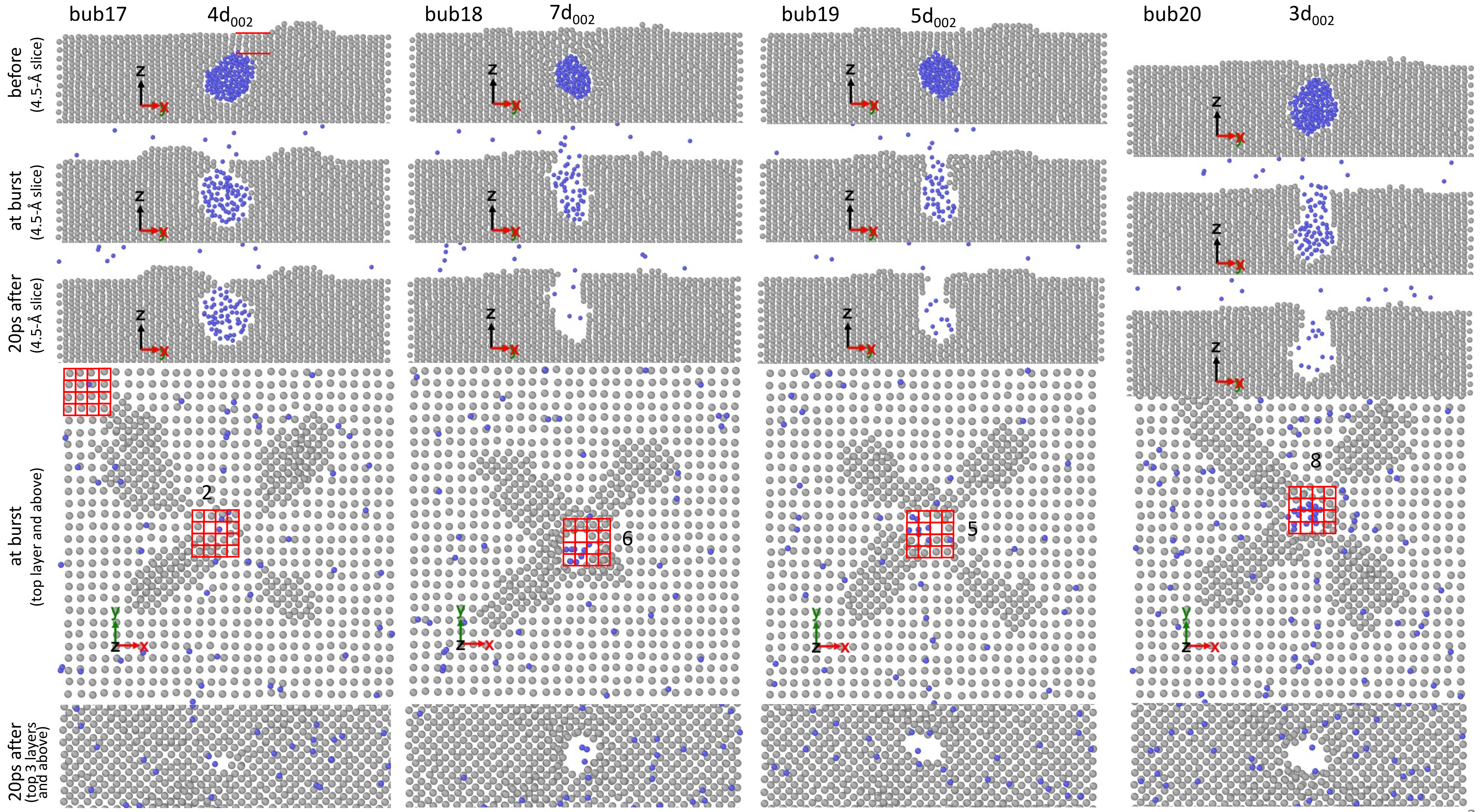

Supplementary Figure 3. Continued.

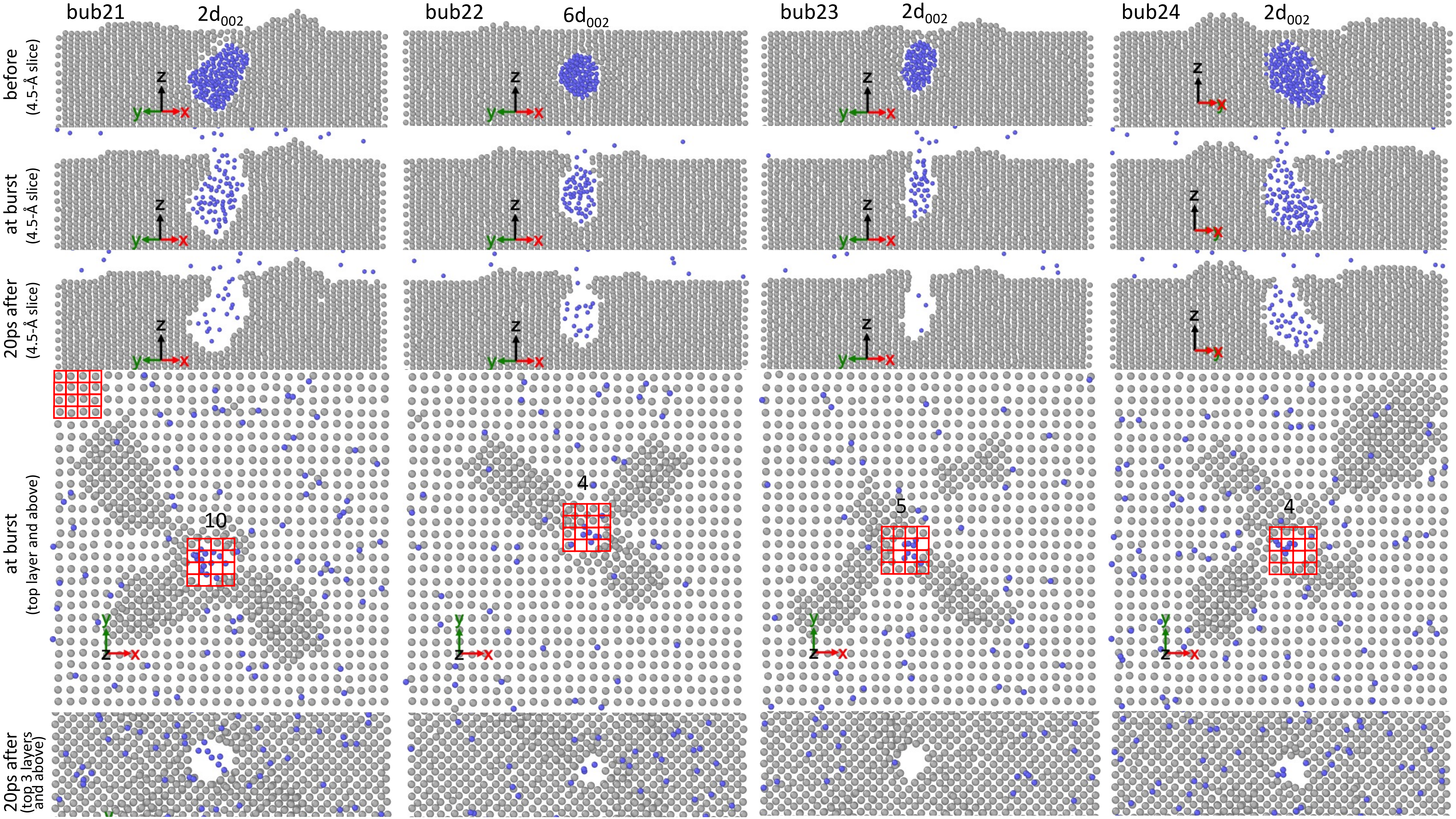

Supplementary Figure 3. Continued.

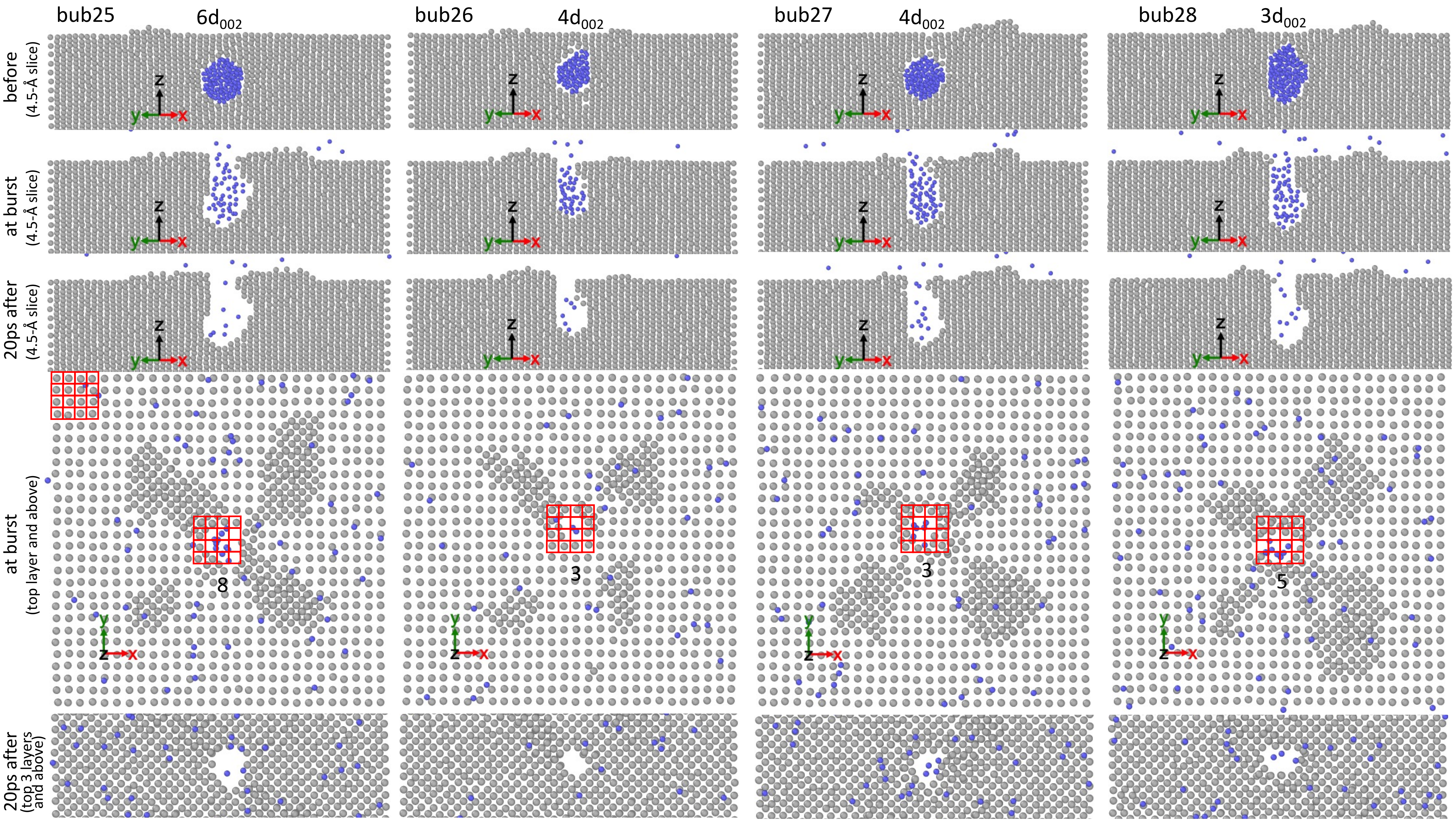

Supplementary Figure 3. Continued.

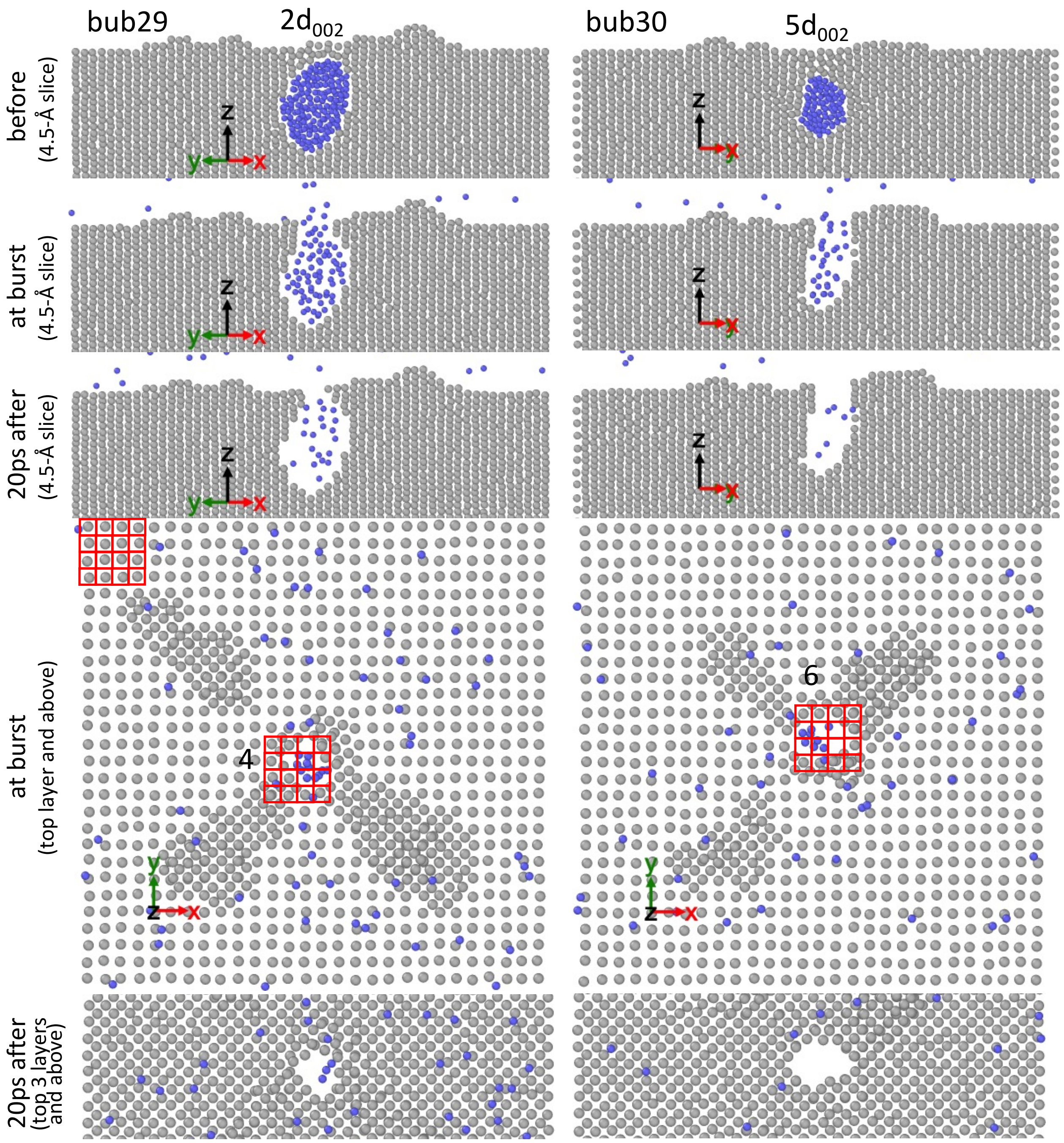

Supplementary Figure 3. Continued.

Supplementary Table 3. Data of thickness of W ligament above the bubble just before bursting (t_{lig}), area of burst hole (A_h), whether or not the bubble is resealed (1: yes, 0: no) after bursting, number of resealed layers, and the fraction of the number of He atoms left if case of resealed bubbles, form bubbles initially nucleated at depth of 13*a*/2.

bub	t_{lig} (d_{002})	A_h (a^2)	reseal?	#resealed layers	f_{He}
1	2	2	1	1	0.271
2	3	4	0		
3	3	7	0		
4	4	6	0		
5	3	8	0		
6	6	4	1	1	0.154
7	4	7	0		
8	4	7	0		
9	5	8	0		
10	1	8	0		
11	5	5	0		
12	6	4	0		
13	5	9	0		
14	5	7	0		
15	4	8	0		
16	4	4	0		
17	4	2	0		
18	7	6	0		
19	5	5	0		
20	3	8	0		
21	2	10	0		
22	6	4	0		
23	2	5	0		
24	2	4	0		
25	6	8	0		
26	4	3	0		
27	4	3	0		
28	3	5	0		
29	2	4	0		
30	5	6	0		
average	4.0	5.7	7%	1.0	21%

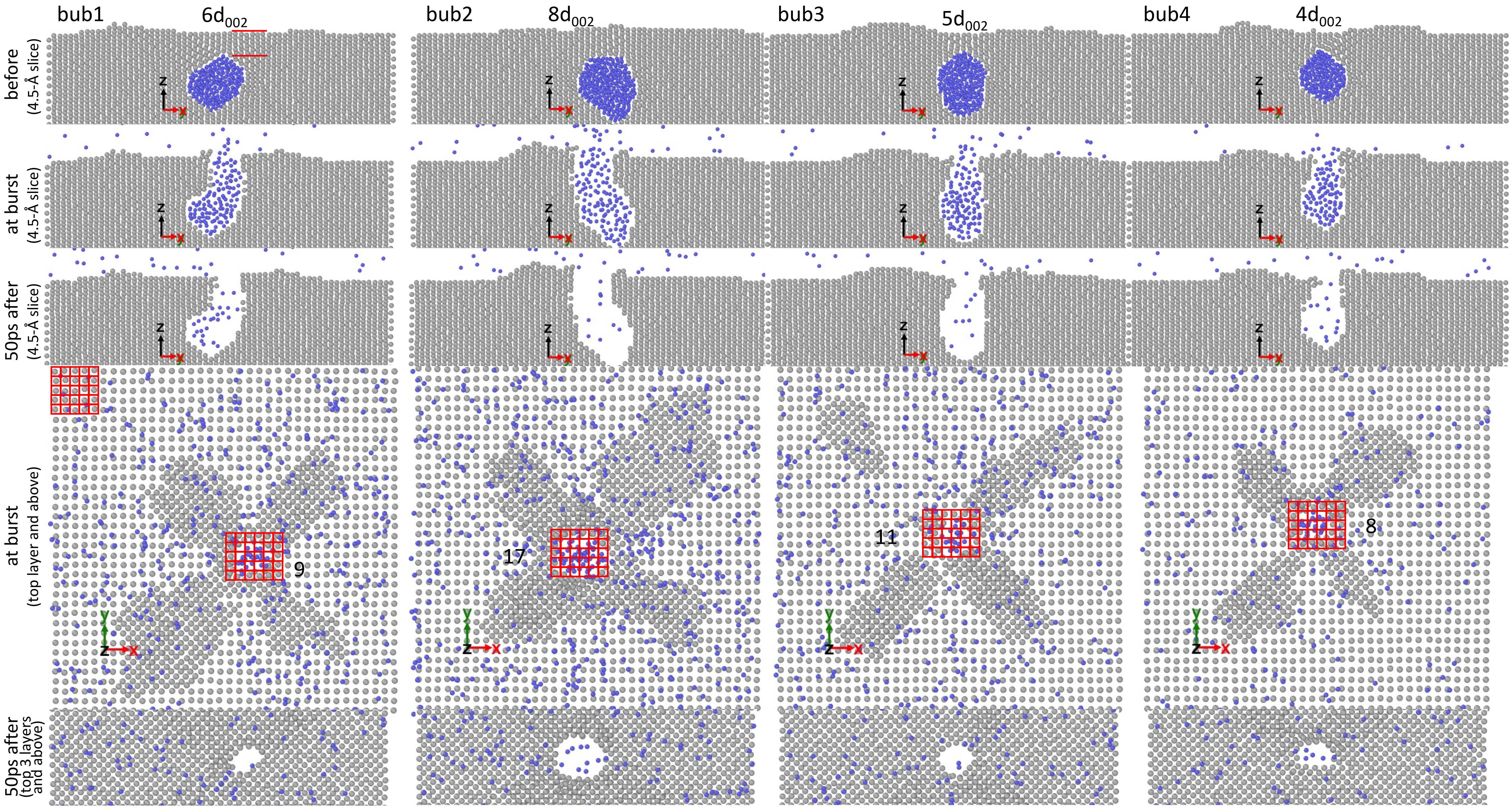

Supplementary Figure 4. Snapshots of bubbles initially nucleated at a depth of 17*a*/2, just before bursting ("before"), just after bursting ("at"), and at the end of the simulations (in this case, "50 ps after" bursting).

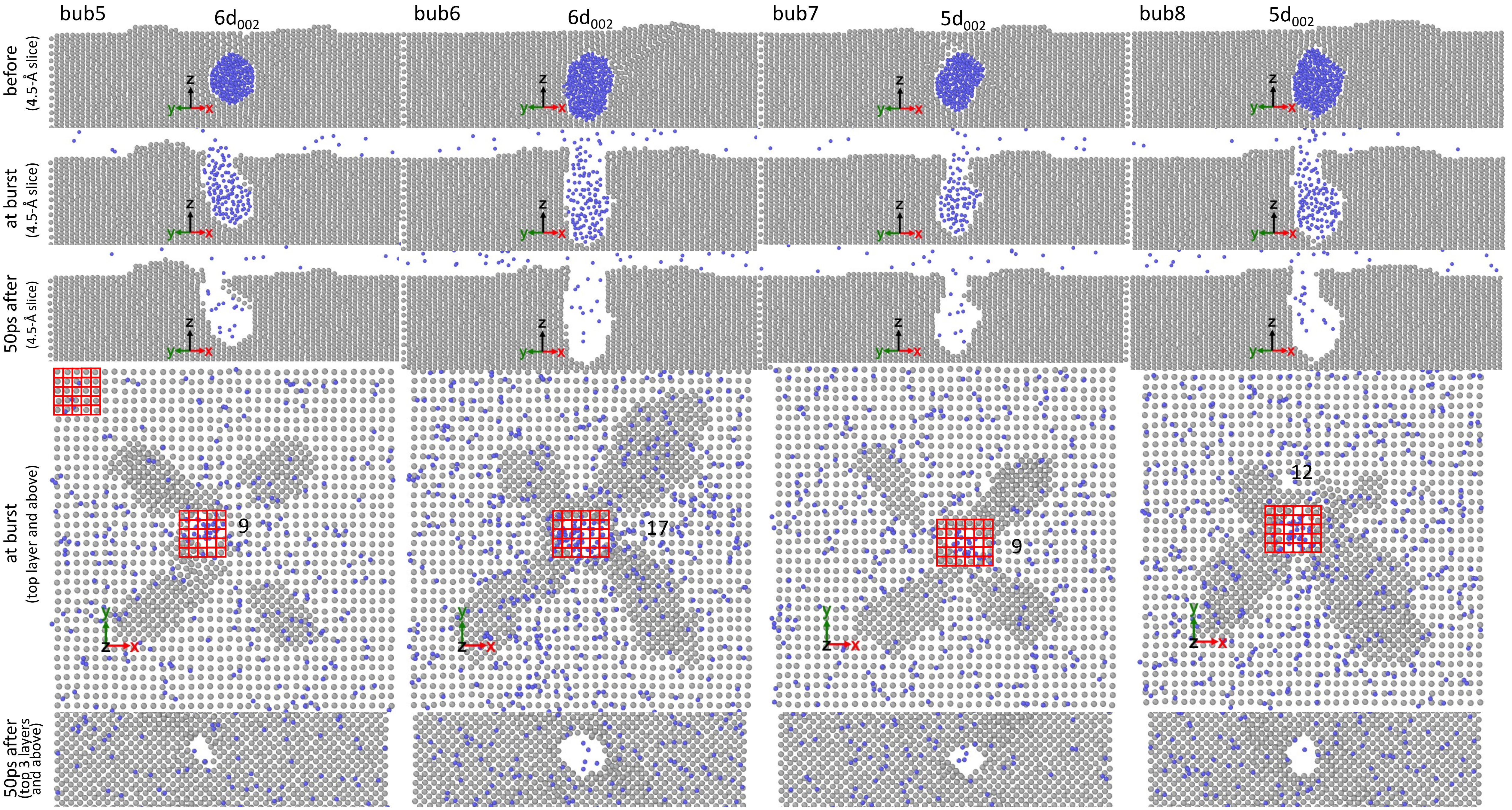

Supplementary Figure 4. Continued.

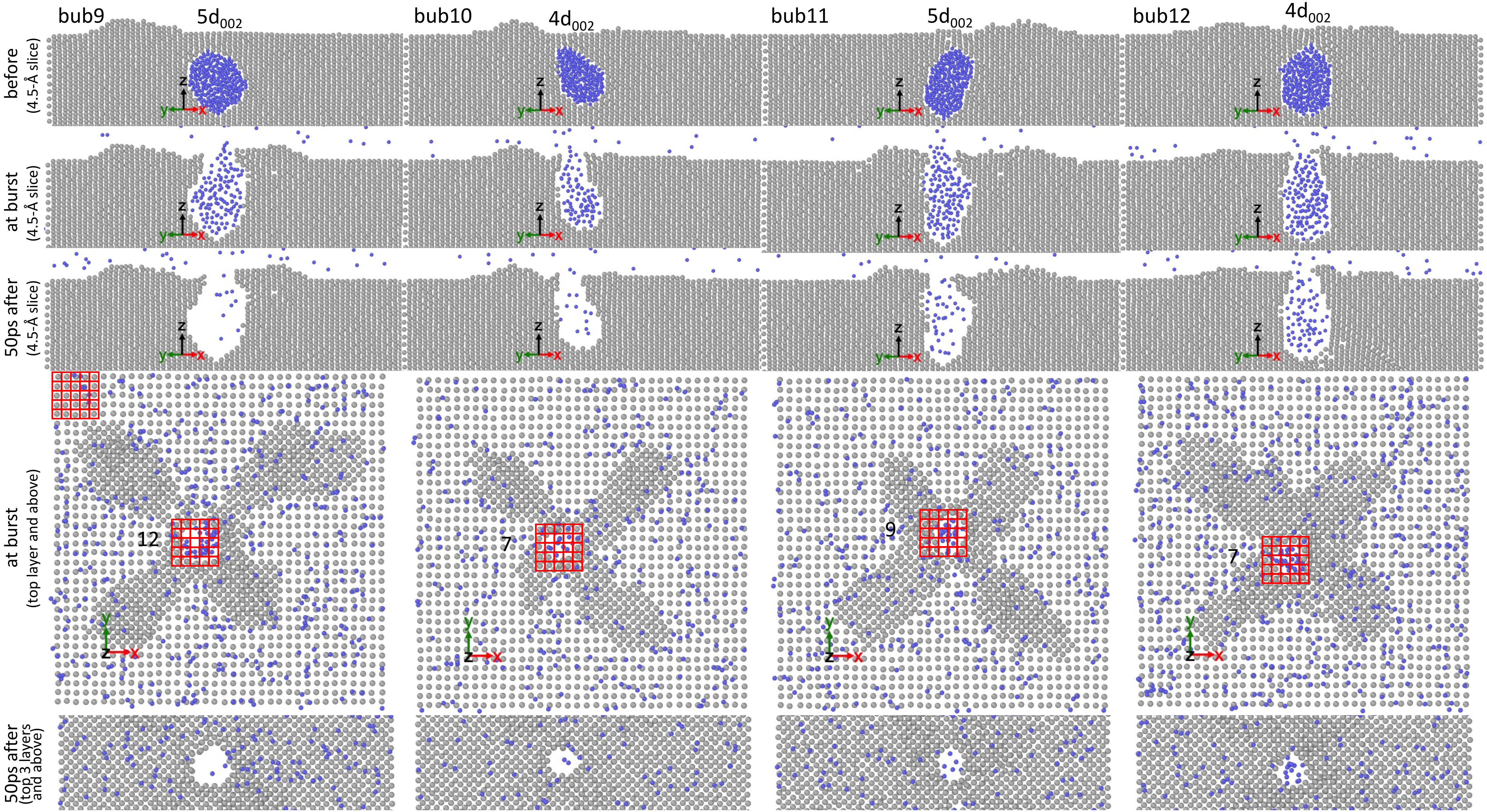

Supplementary Figure 4. Continued.

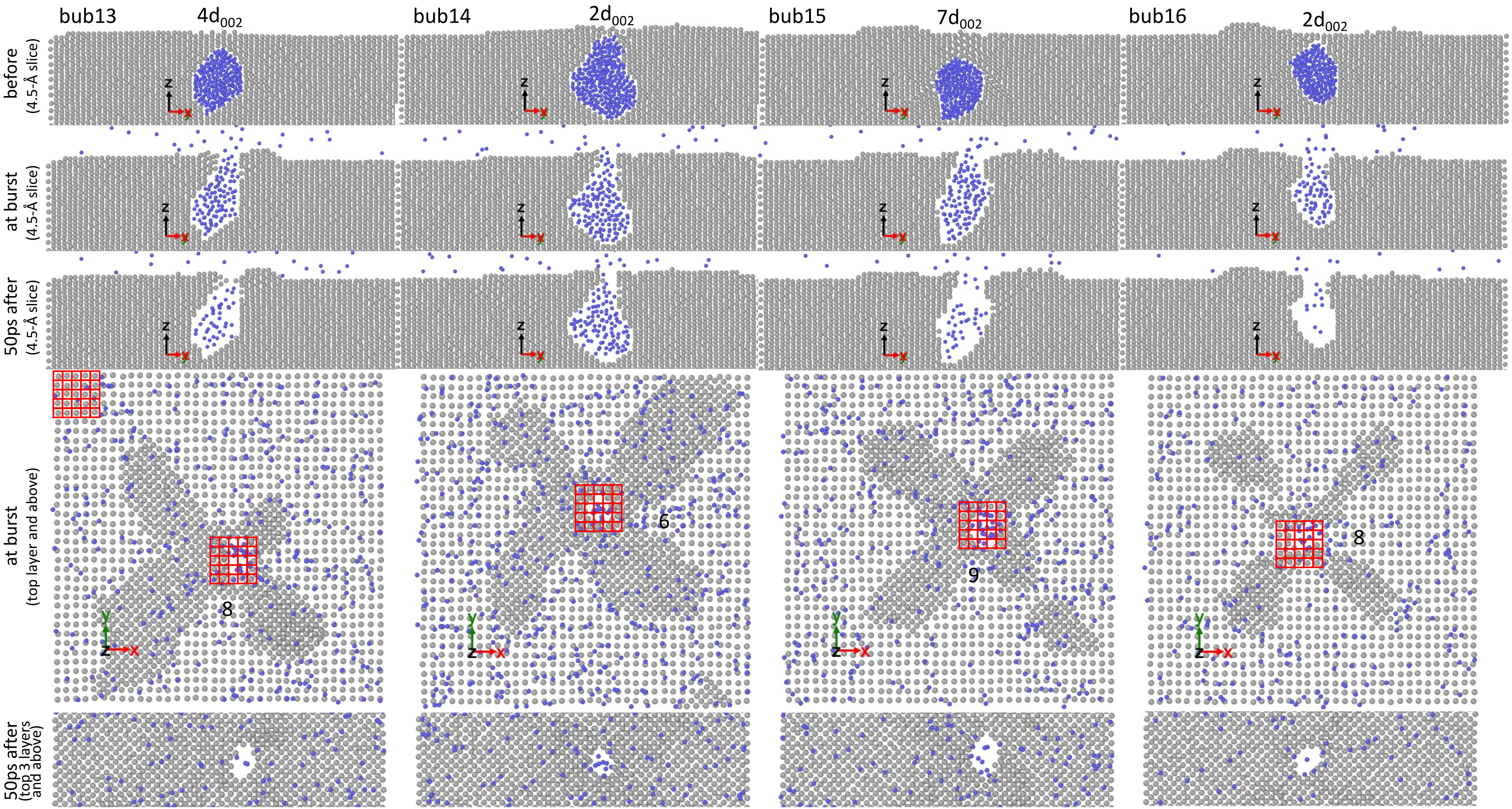

Supplementary Figure 4. Continued.

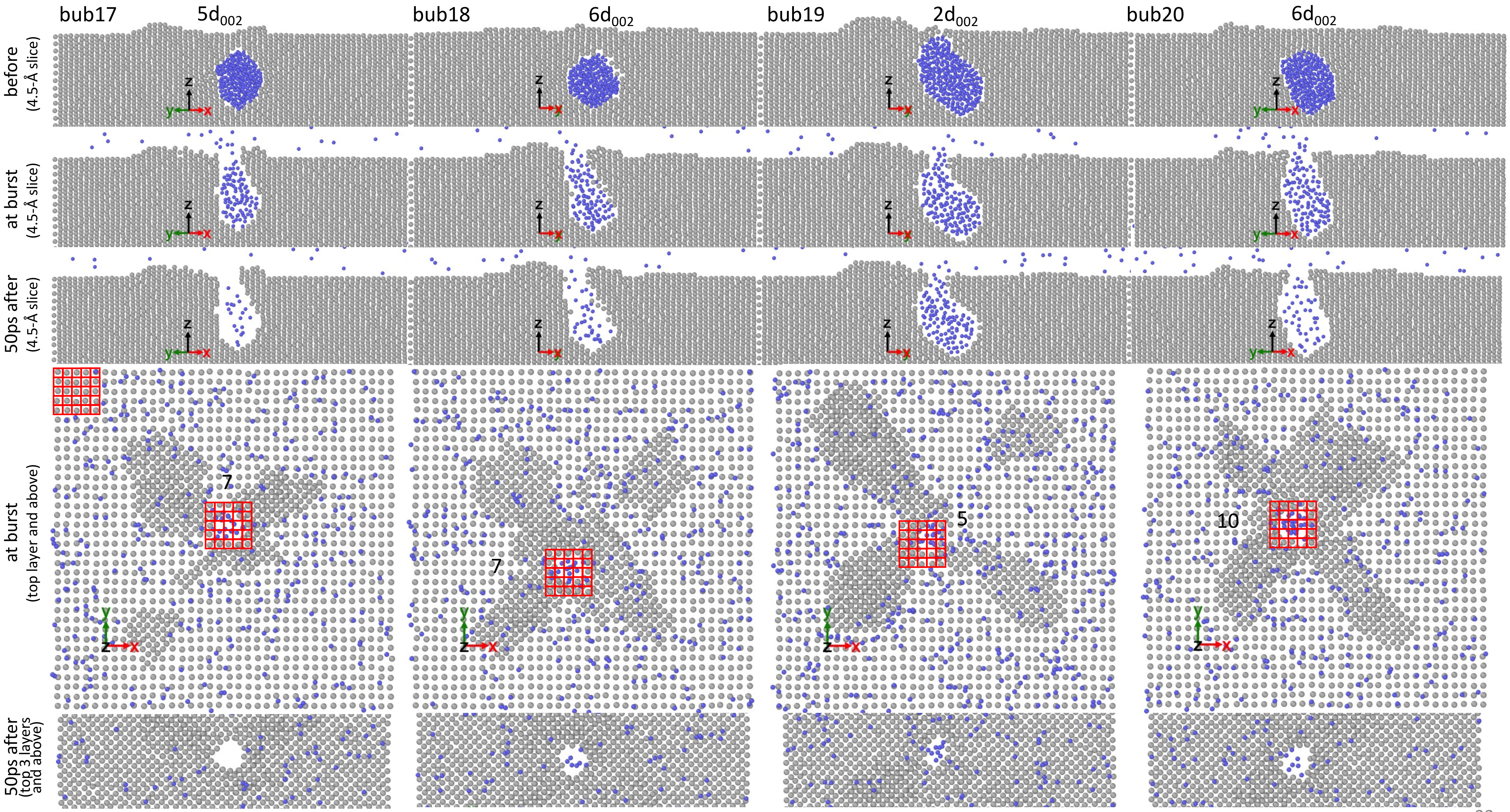

Supplementary Figure 4. Continued.

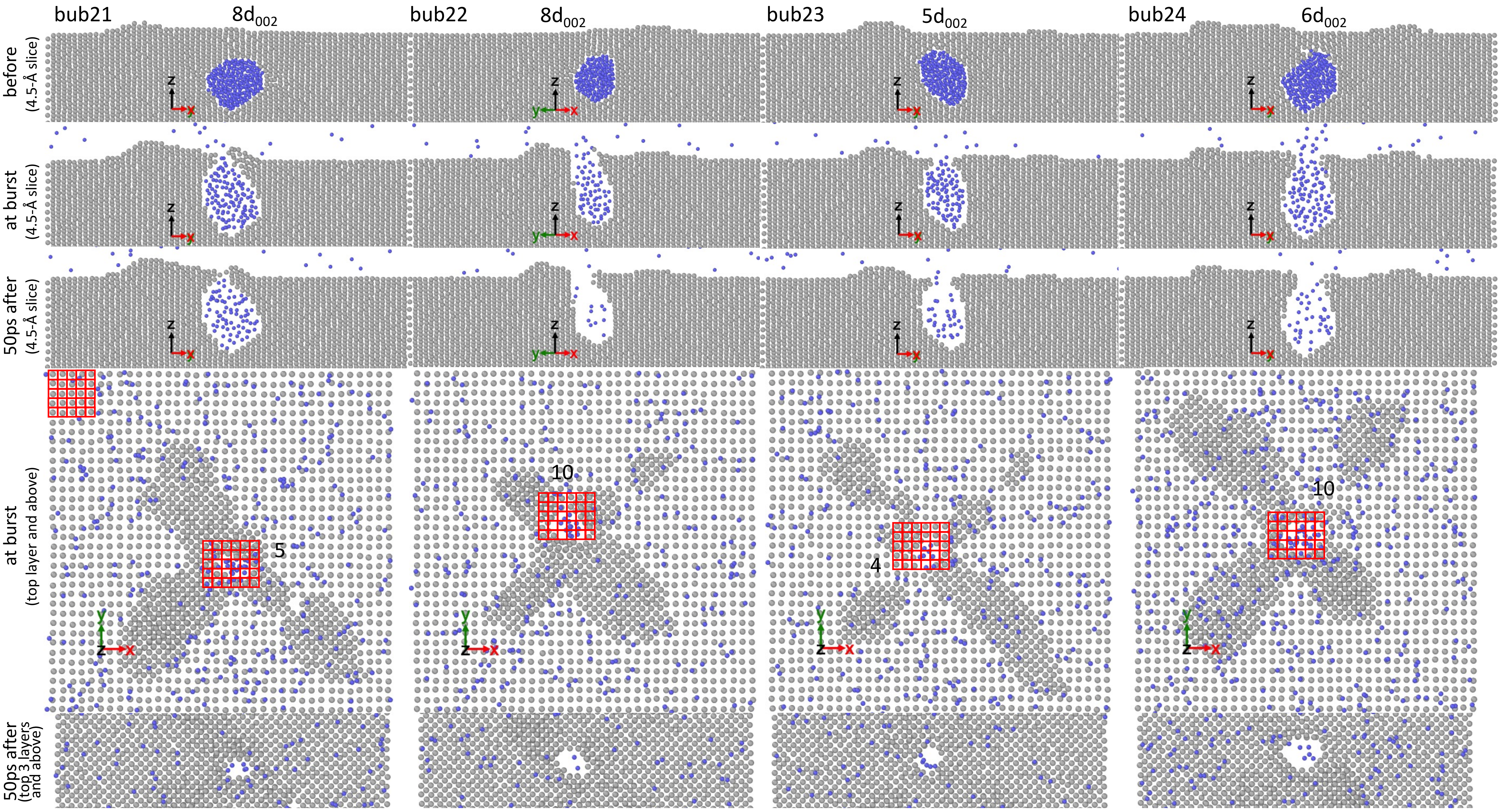

Supplementary Figure 4. Continued.

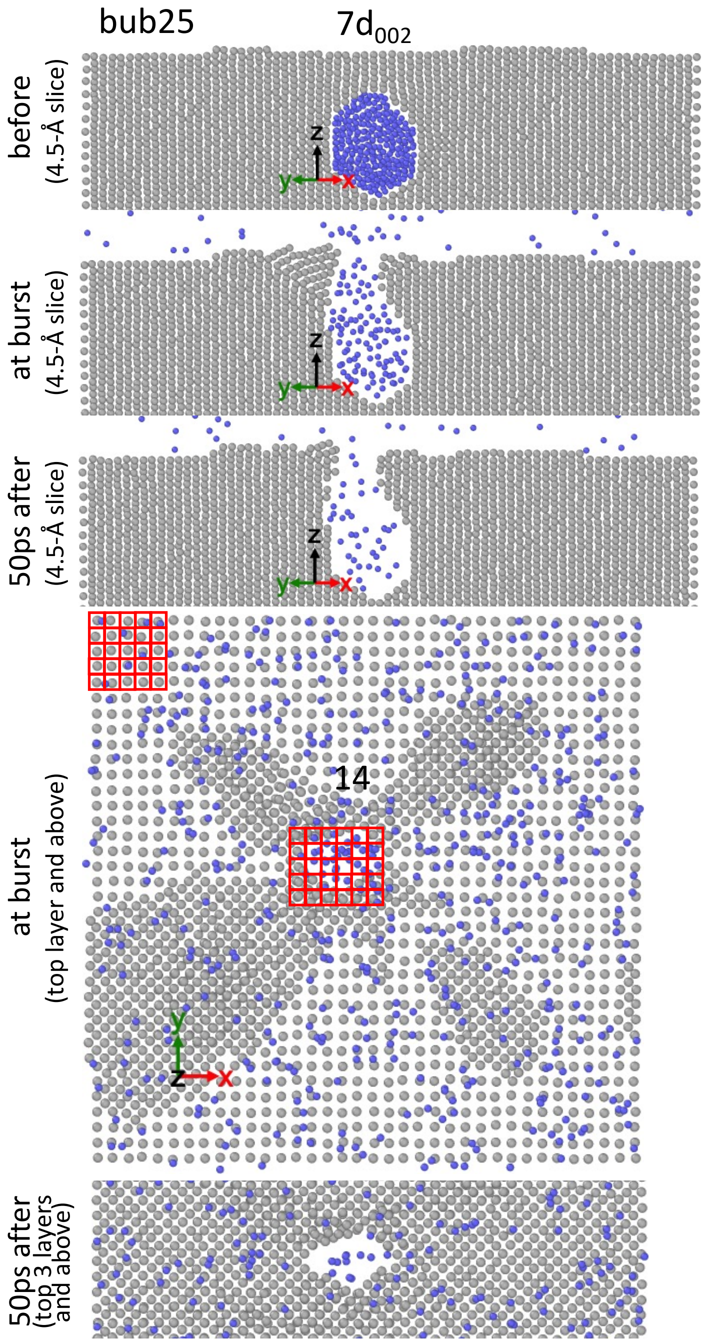

Supplementary Figure 4. Continued.

Supplementary Table 4. Data of thickness of W ligament above the bubble just before bursting (t_{lig}), area of burst hole (A_h), whether or not the bubble is resealed (1: yes, 0: no) after bursting, form bubbles initially nucleated at depth of 17a/2.

bub	t_{lig} (d_{002})	A_h (a^2)	reseal?
1	6	9	0
2	8	17	0
3	5	11	0
4	4	8	0
5	6	9	0
6	6	17	0
7	5	9	0
8	5	12	0
9	5	12	0
10	4	7	0
11	5	9	0
12	4	7	0
13	4	8	0
14	2	6	0
15	7	9	0
16	2	8	0
17	5	7	0
18	6	7	0
19	2	5	0
20	6	10	0
21	8	5	0
22	8	10	0
23	5	4	0
24	6	10	0
25	7	14	0
average	5.2	9.2	0.0

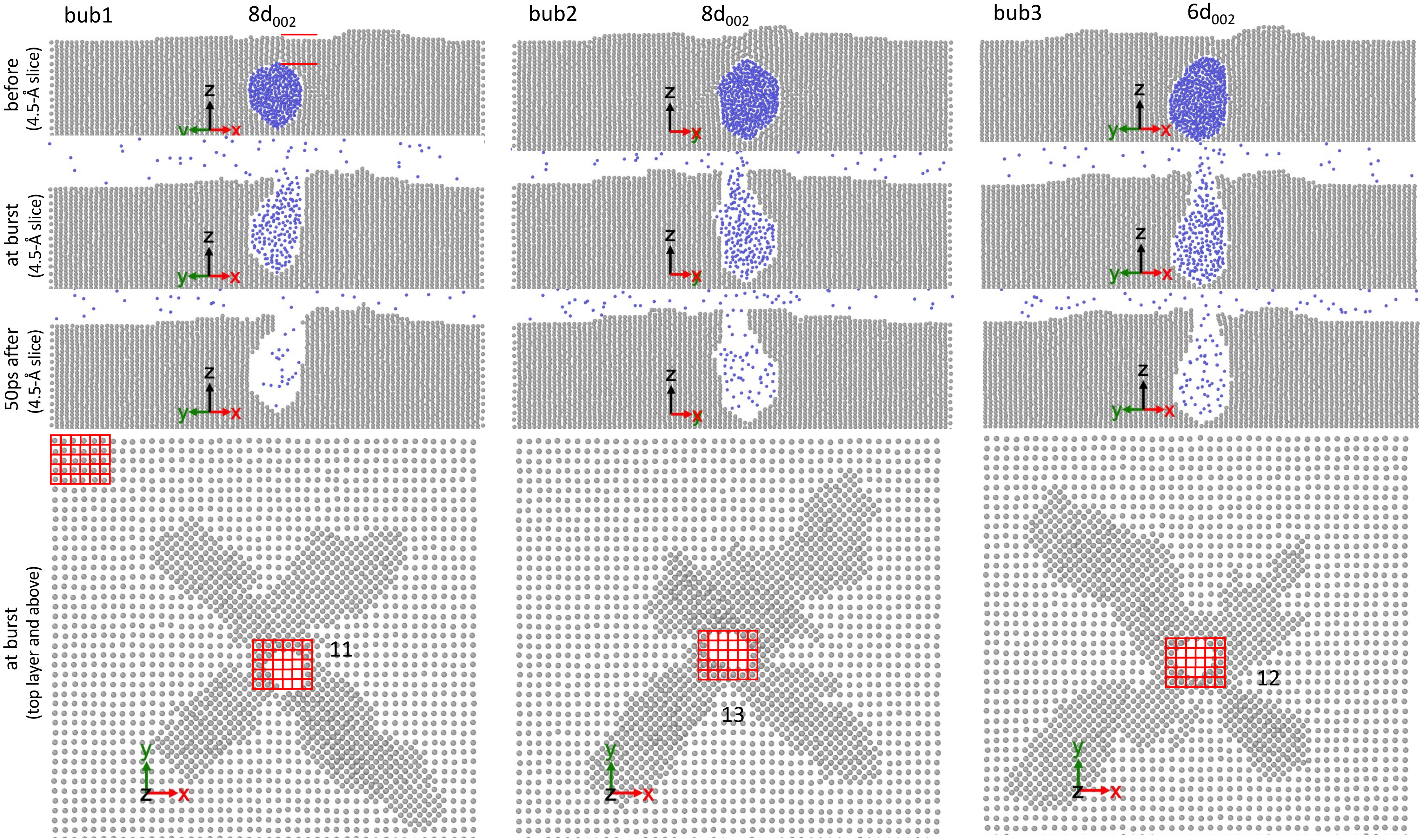

Supplementary Figure 5. Snapshots of bubbles initially nucleated at a depth of 21*a*/2, just before bursting ("before"), just after bursting ("at"), and at the end of the simulations (in this case, "50 ps after" bursting).

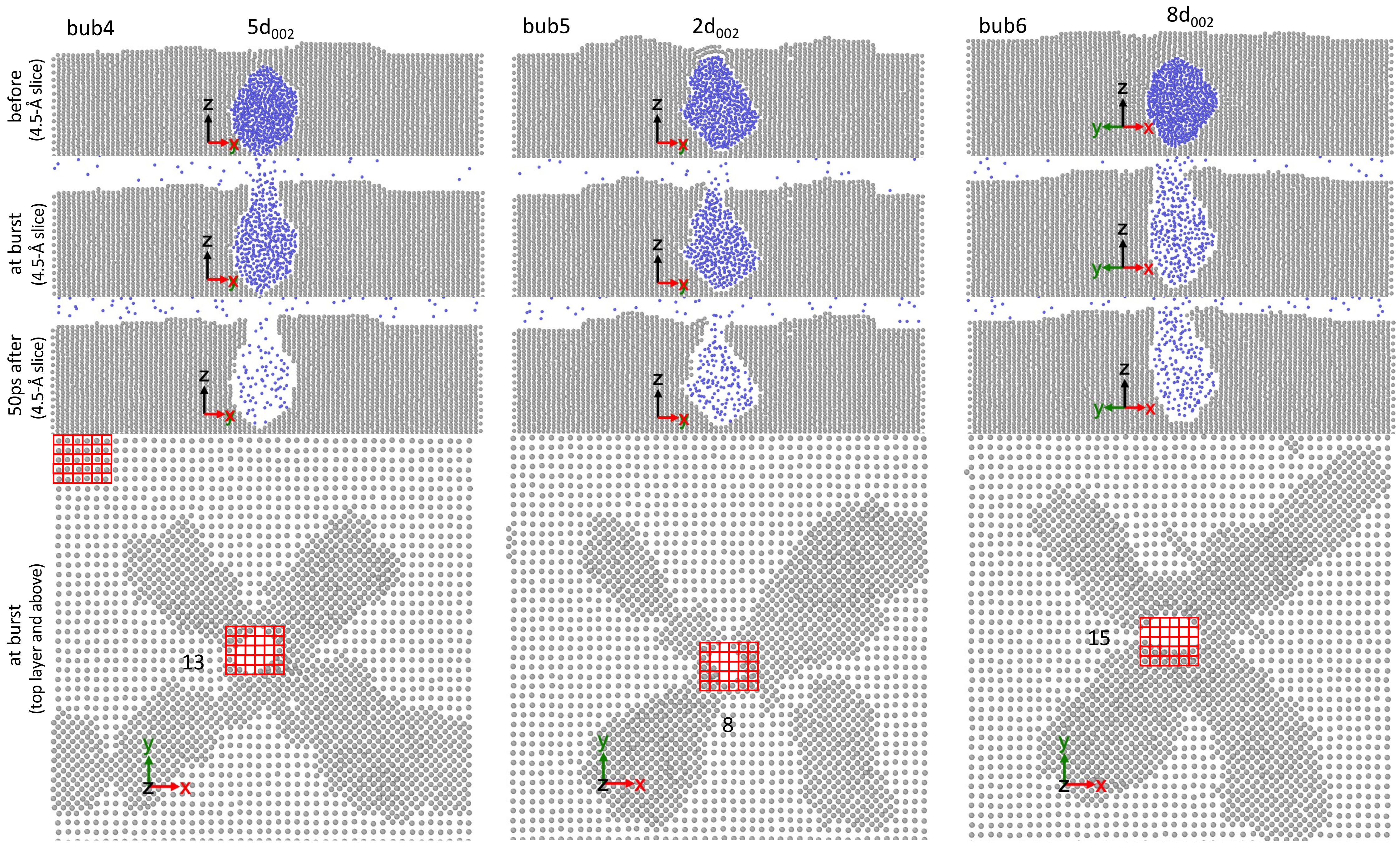

Supplementary Figure 5. Continued.

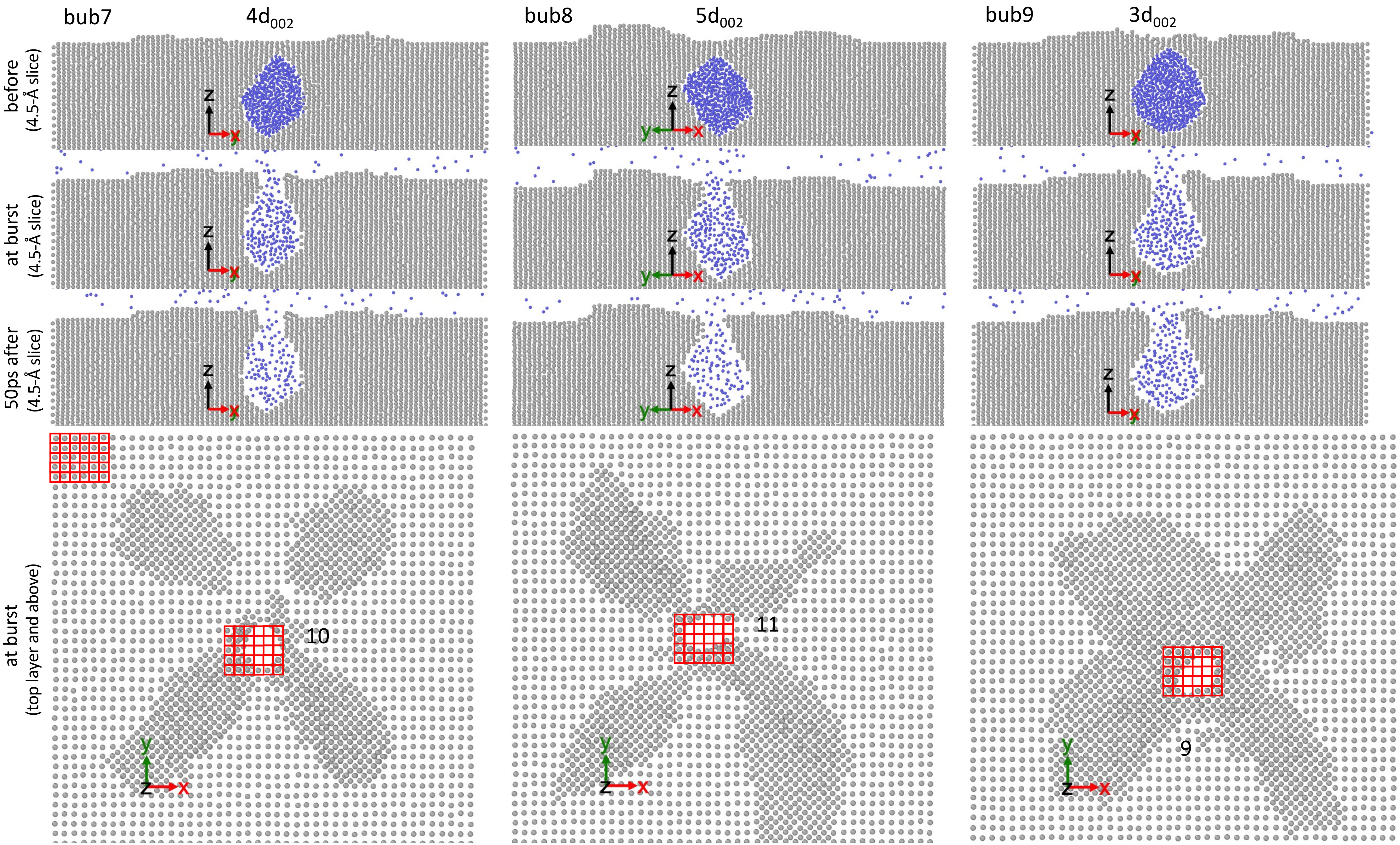

Supplementary Figure 5. Continued.

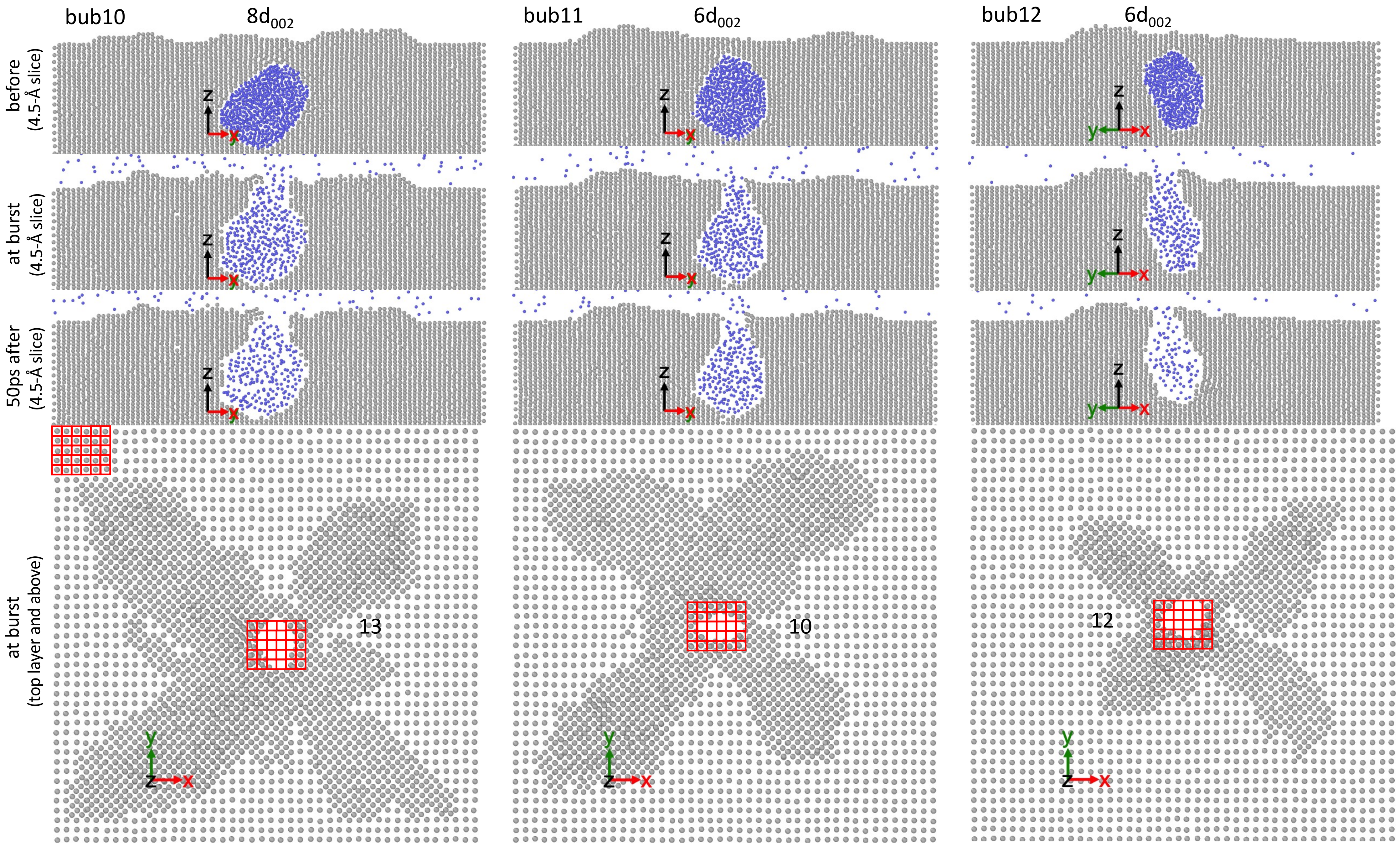

Supplementary Figure 5. Continued.

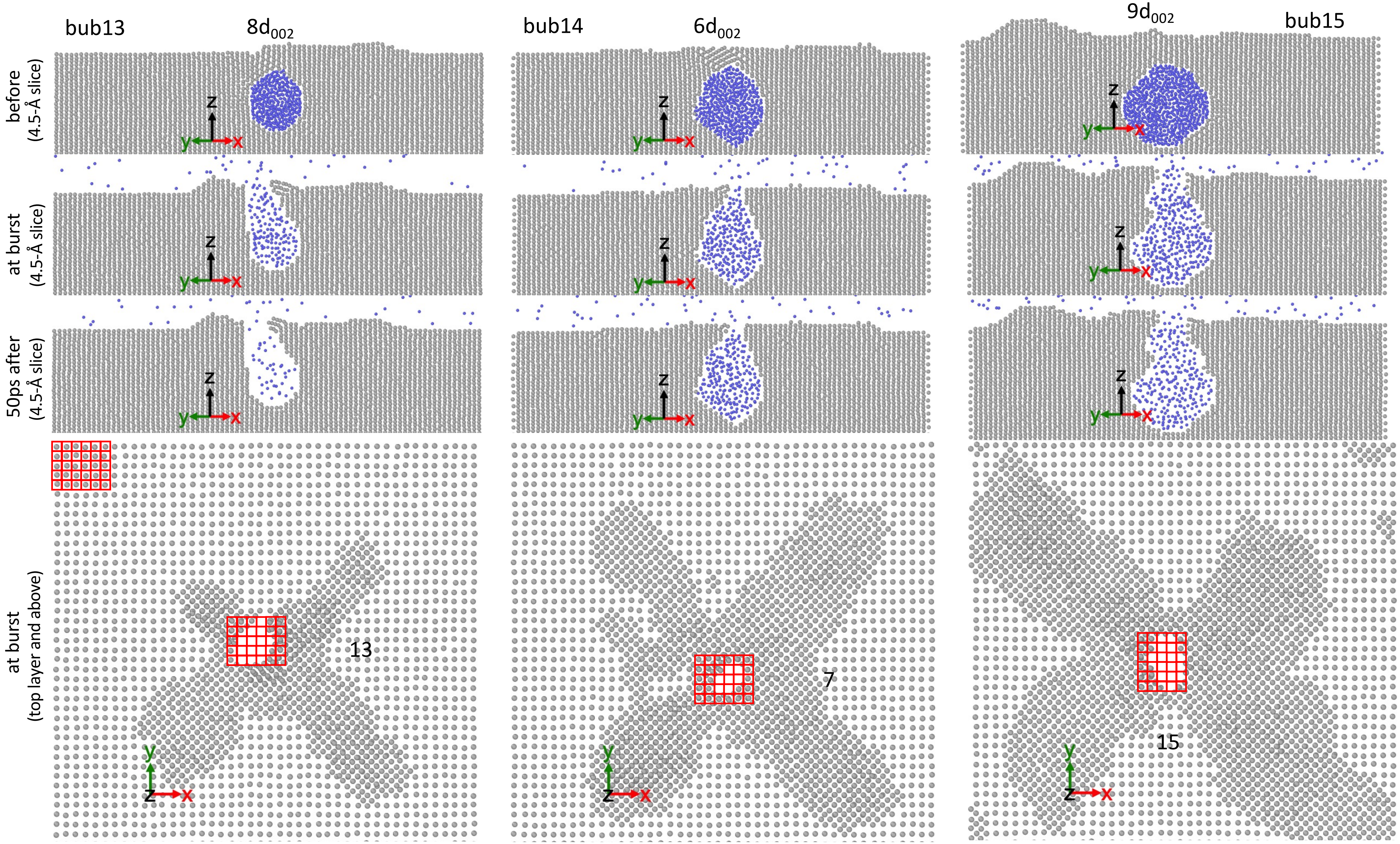

Supplementary Figure 5. Continued.

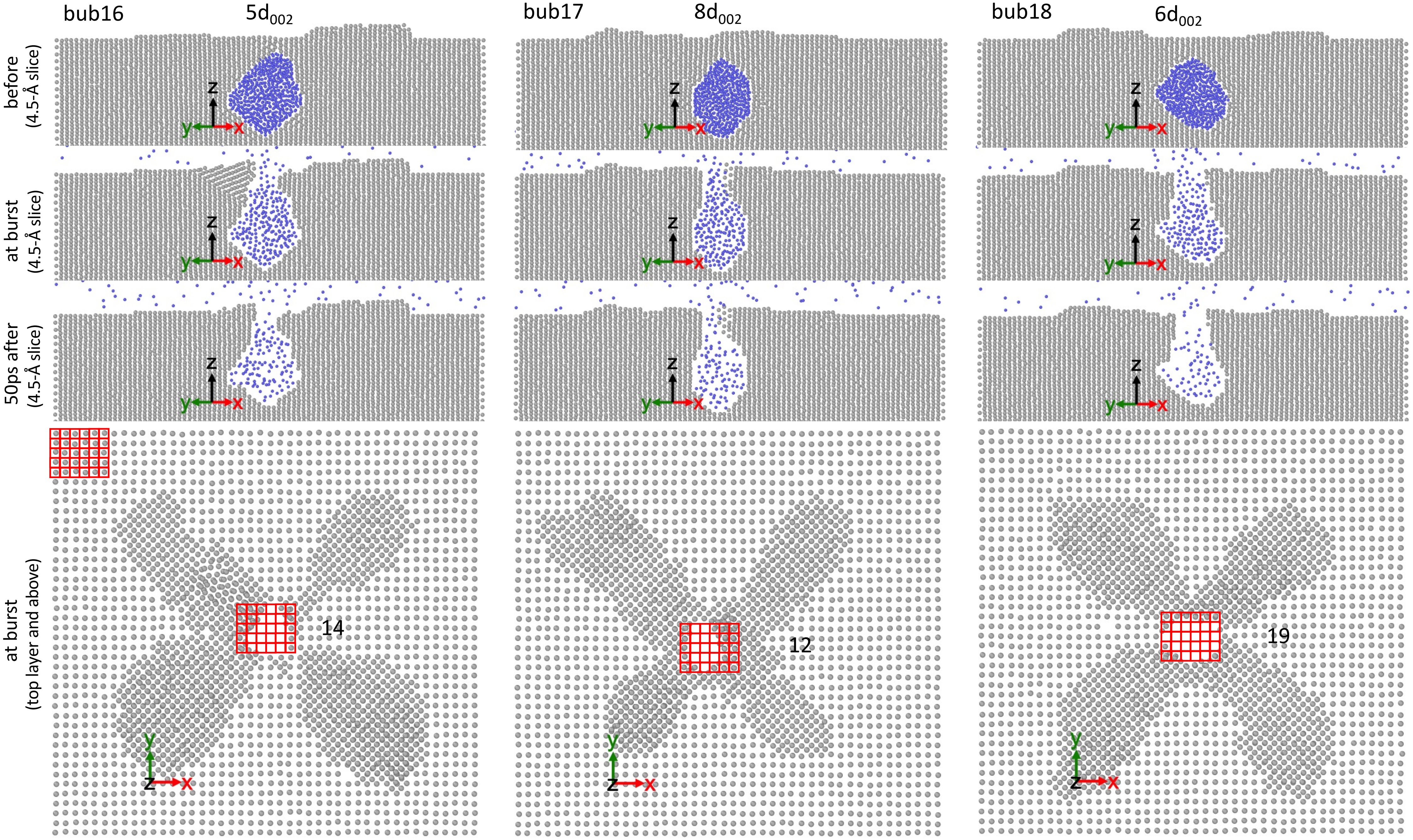

Supplementary Figure 5. Continued.

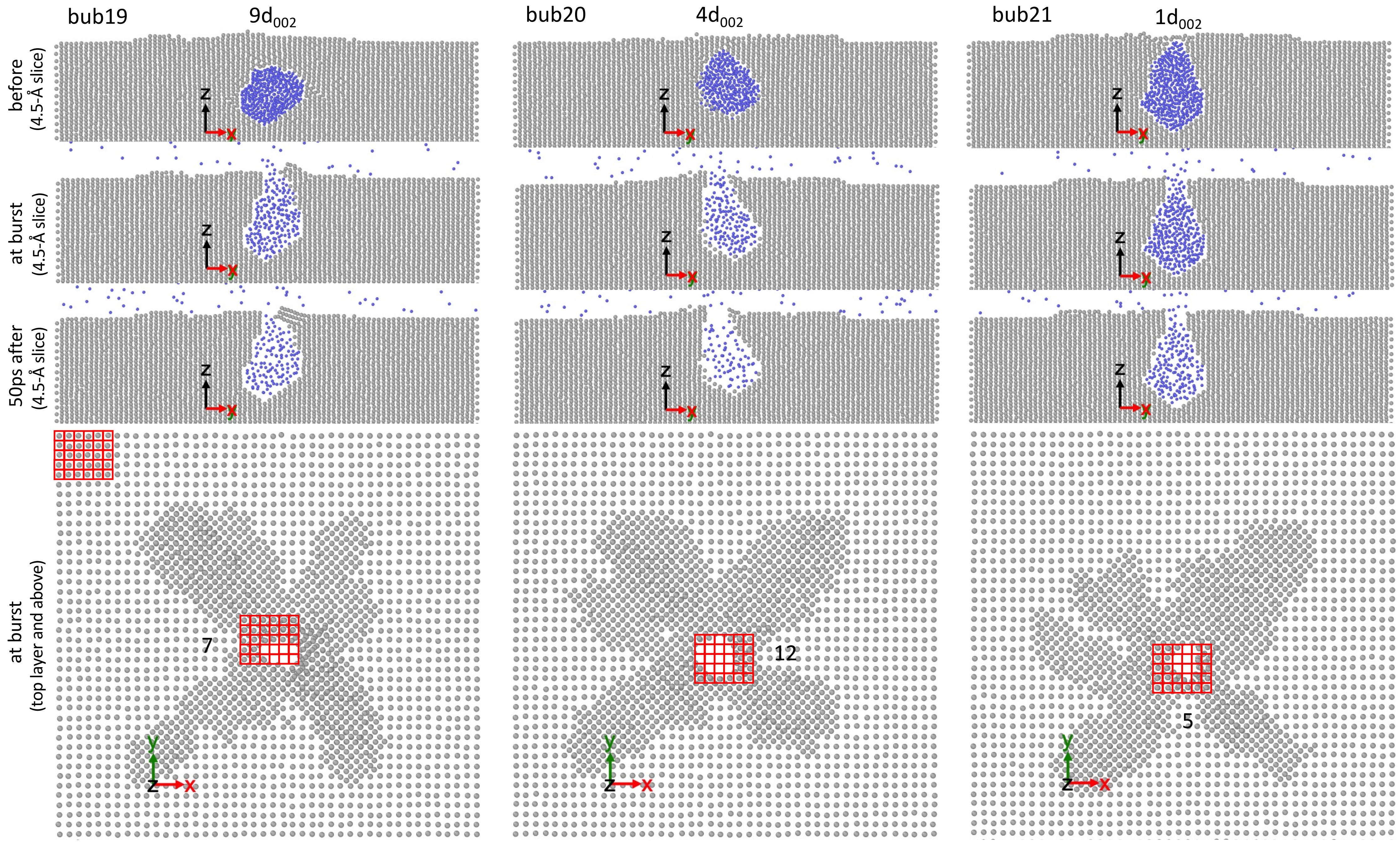

Supplementary Figure 5. Continued.

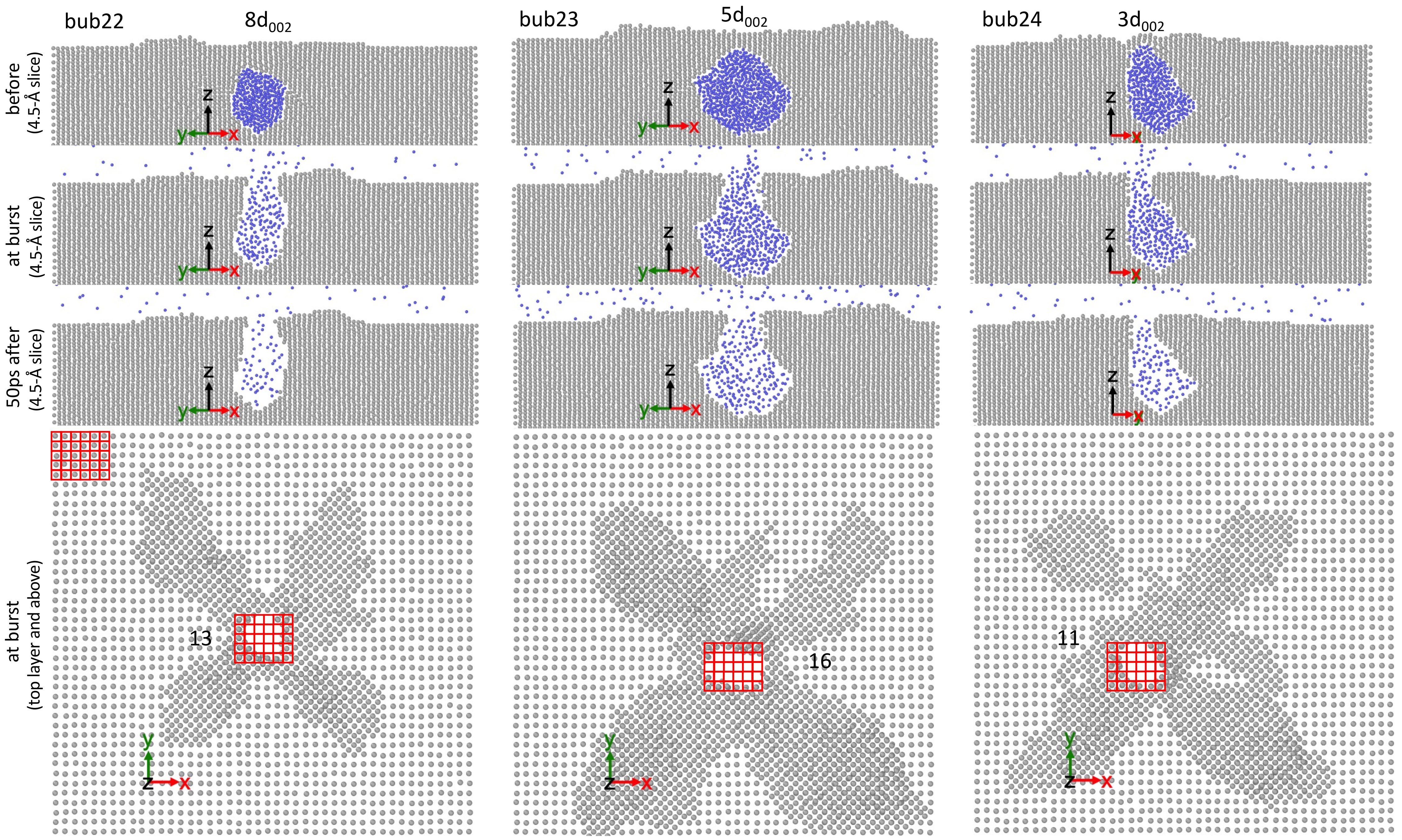

Supplementary Figure 5. Continued.

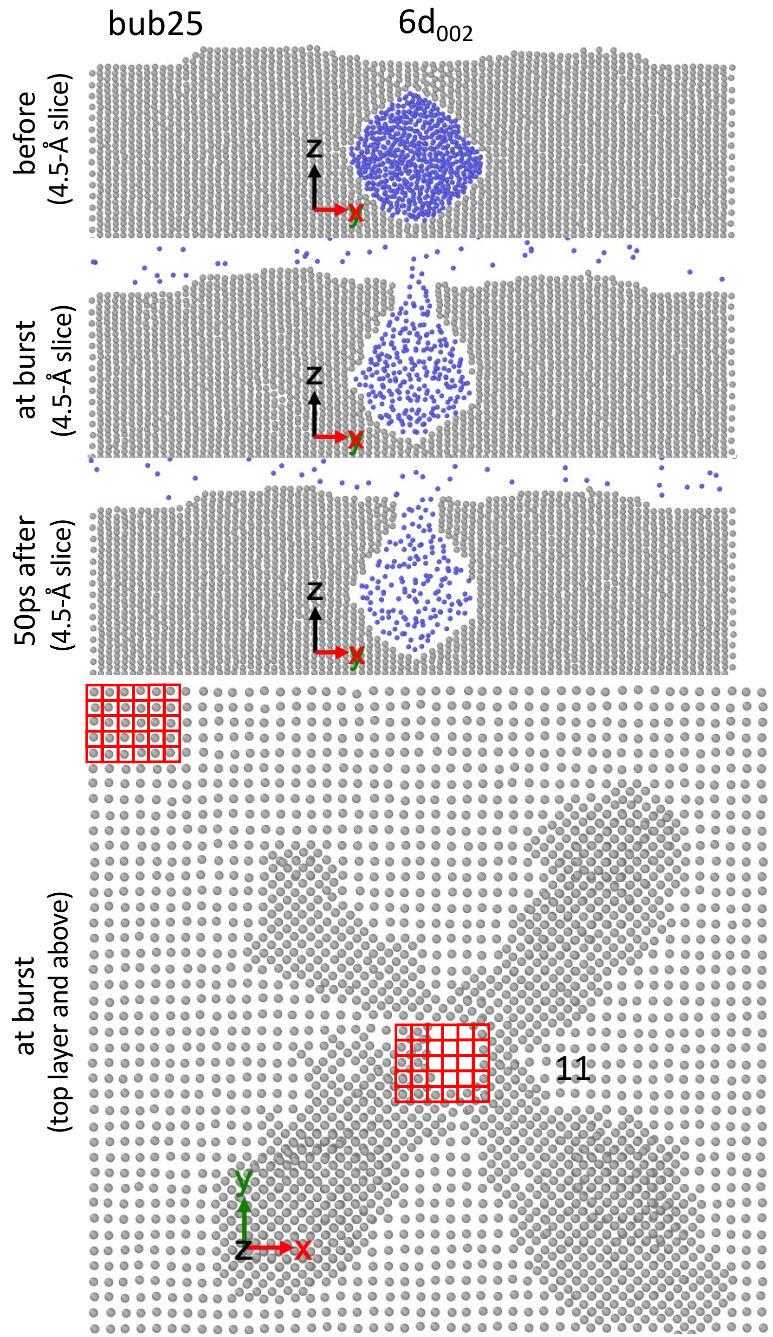

Supplementary Figure 5. Continued.

Supplementary Table 5. Data of thickness of W ligament above the bubble just before bursting (t_{lig}), area of burst hole (A_h), whether or not the bubble is resealed (1: yes, 0: no) after bursting, form bubbles initially nucleated at depth of 21a/2.

bub	t_{lig} (d_{002})	A_h (a^2)	reseal?
1	8	11	0
2	8	13	0
3	6	12	0
4	5	13	0
5	2	8	0
6	8	15	0
7	4	10	0
8	5	11	0
9	3	9	0
10	8	13	0
11	6	10	0
12	6	12	0
13	8	13	0
14	6	7	0
15	9	15	0
16	5	14	0
17	8	12	0
18	6	19	0
19	9	7	0
20	4	12	0
21	1	5	0
22	8	13	0
23	5	16	0
24	3	11	0
25	6	11	0
average	5.9	11.7	0.0

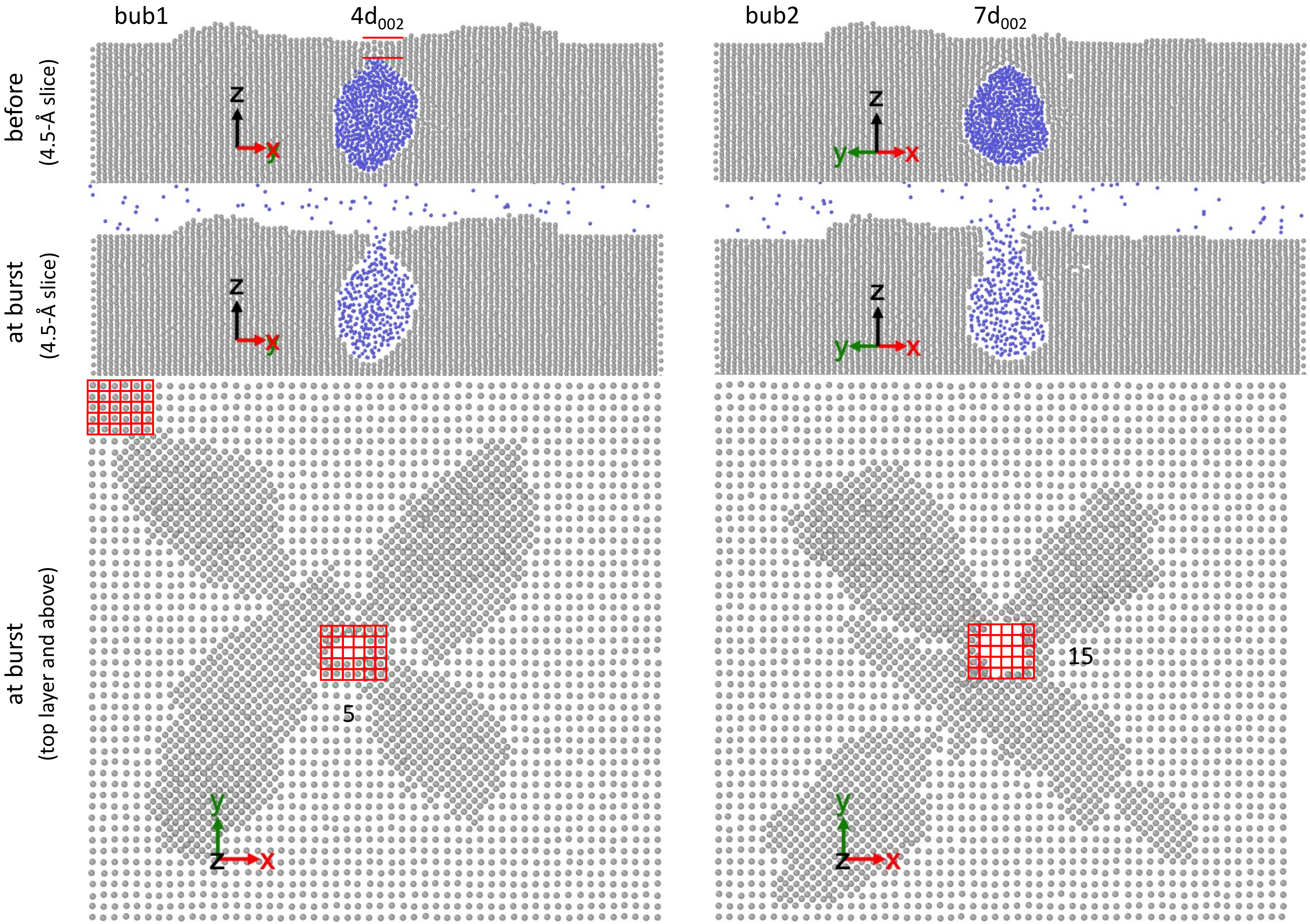

Supplementary Figure 6. Snapshots of bubbles initially nucleated at a depth of 25a/2, just before bursting ("before") and just after bursting ("at").

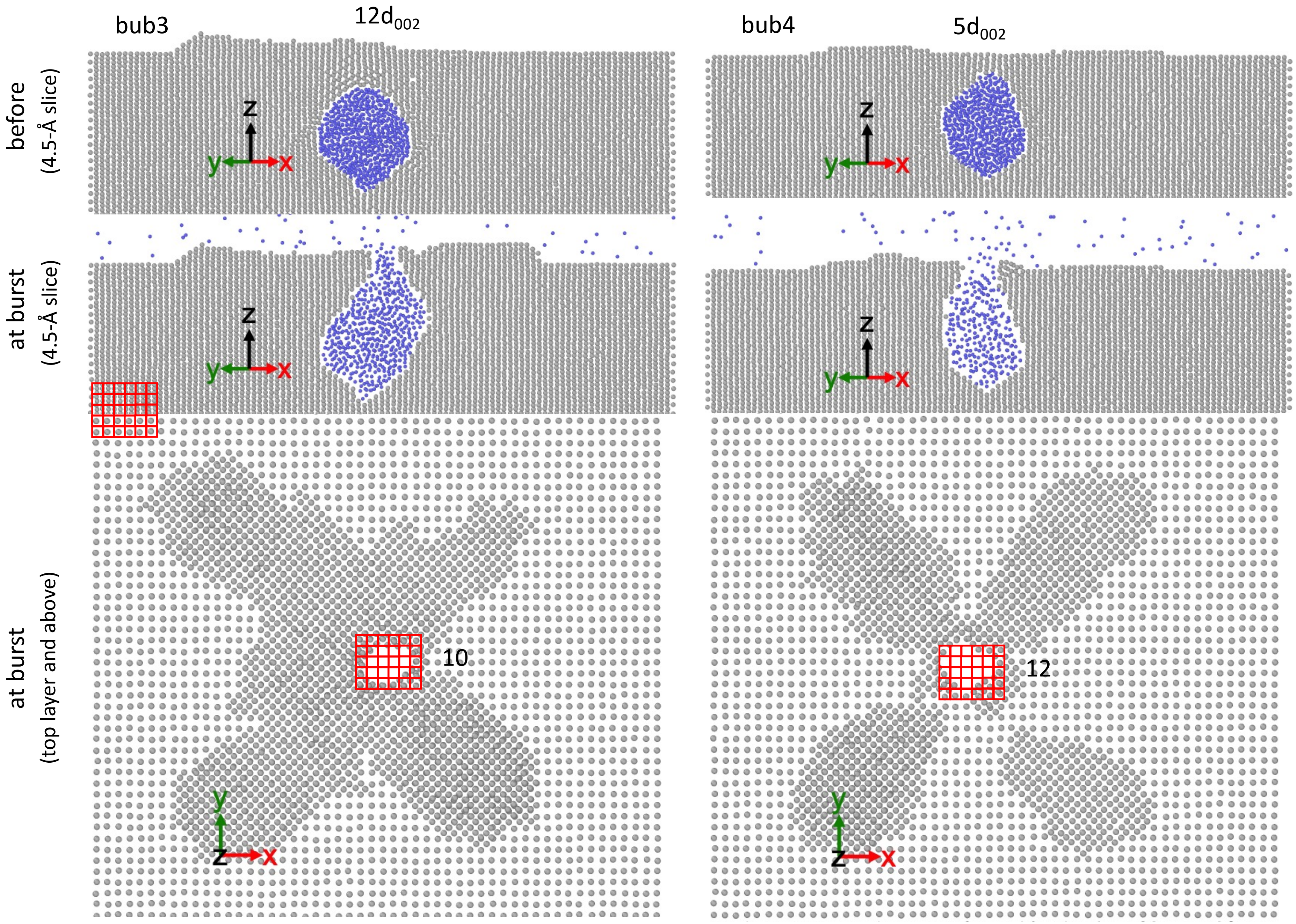

Supplementary Figure 6. Continued.

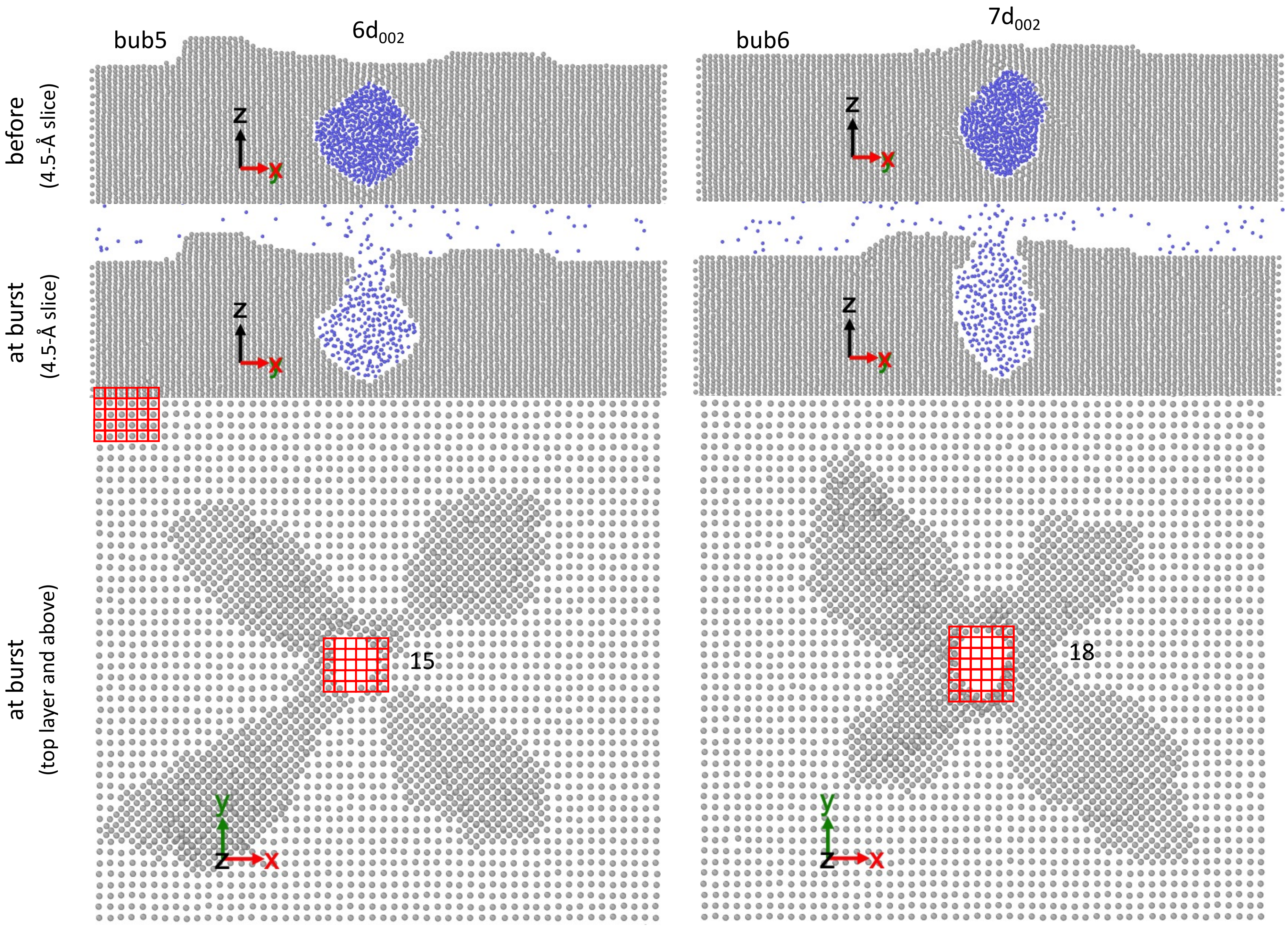

Supplementary Figure 6. Continued.

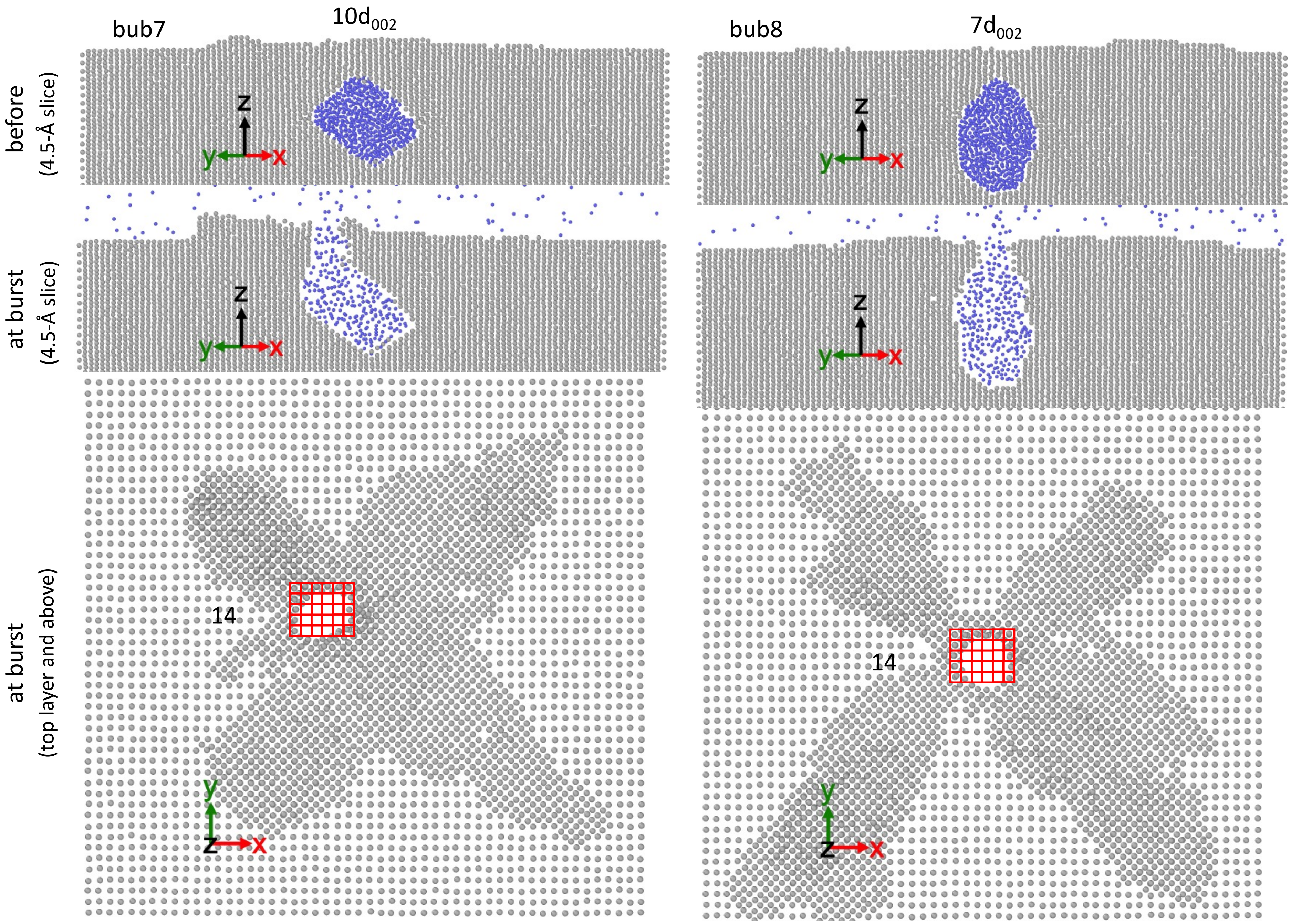

Supplementary Figure 6. Continued.

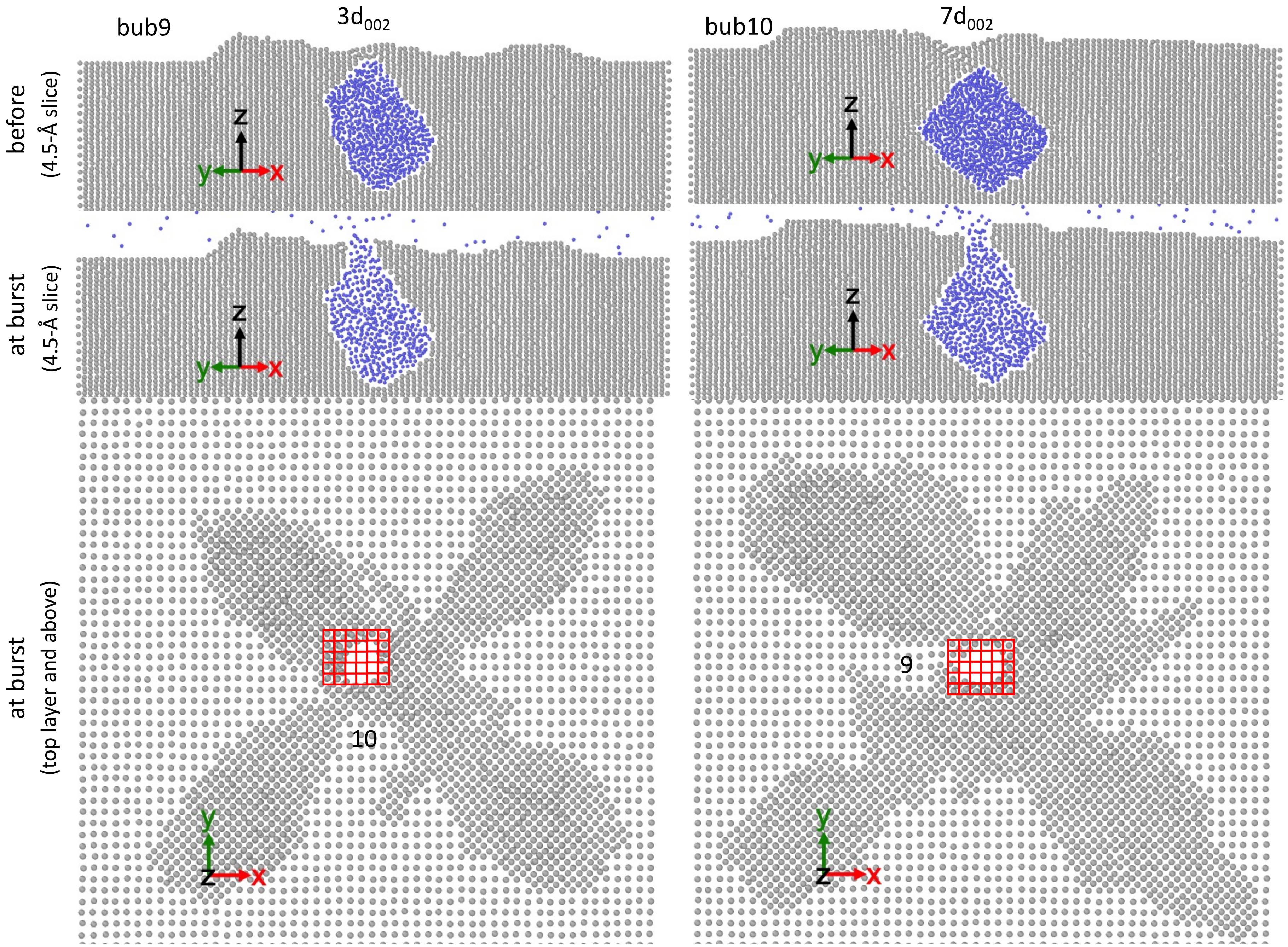

Supplementary Figure 6. Continued.

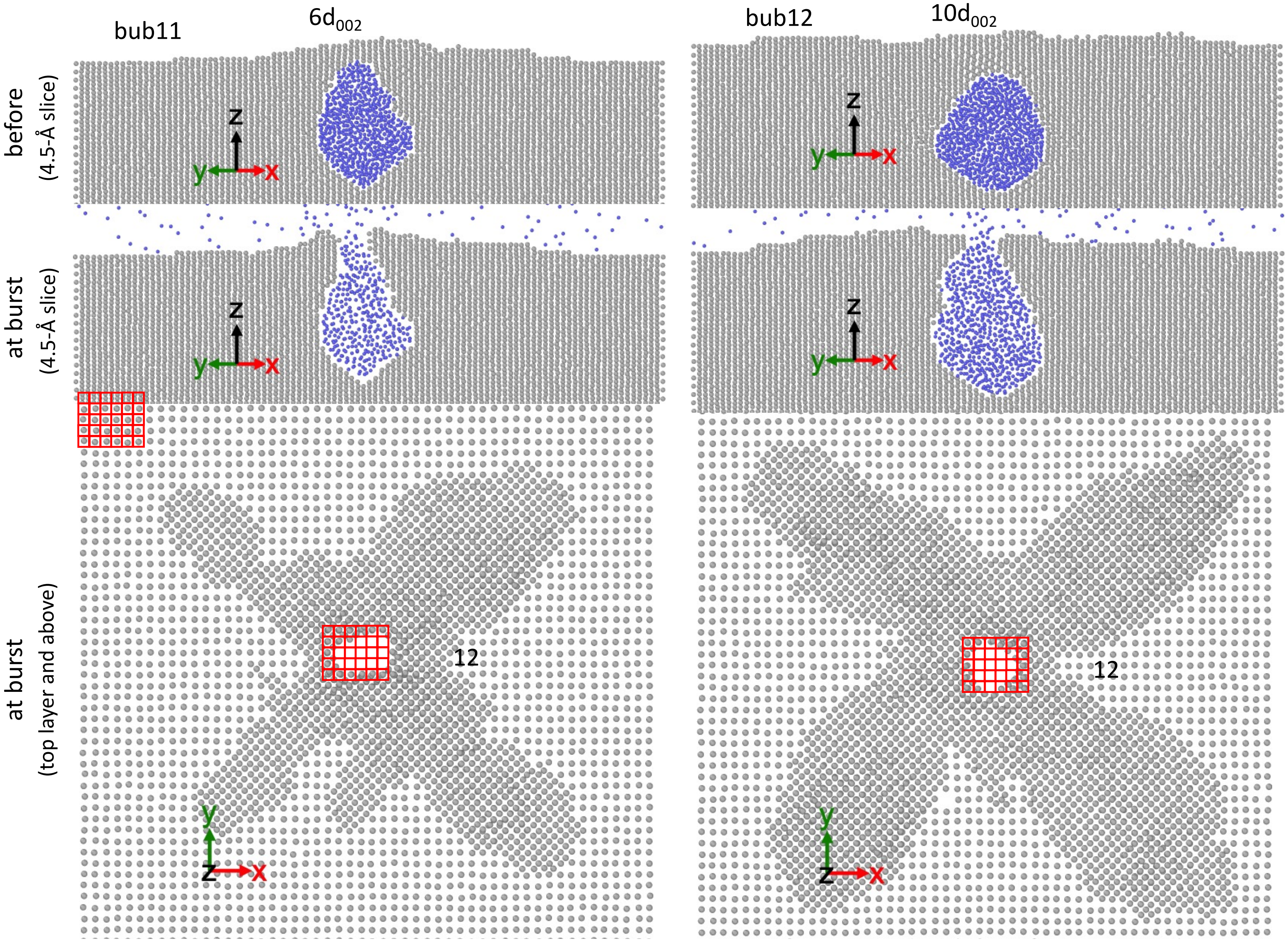

Supplementary Figure 6. Continued.

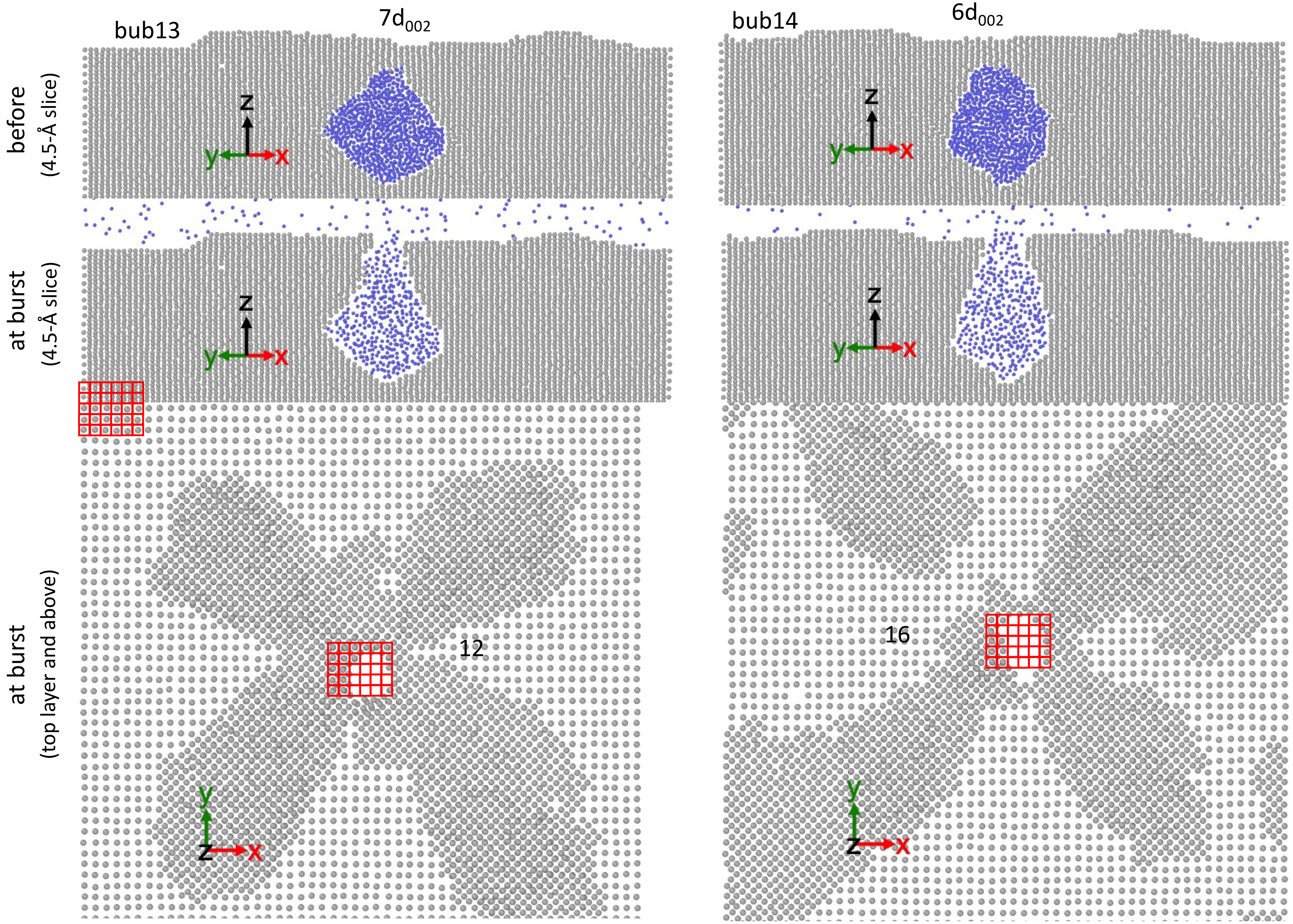

Supplementary Figure 6. Continued.

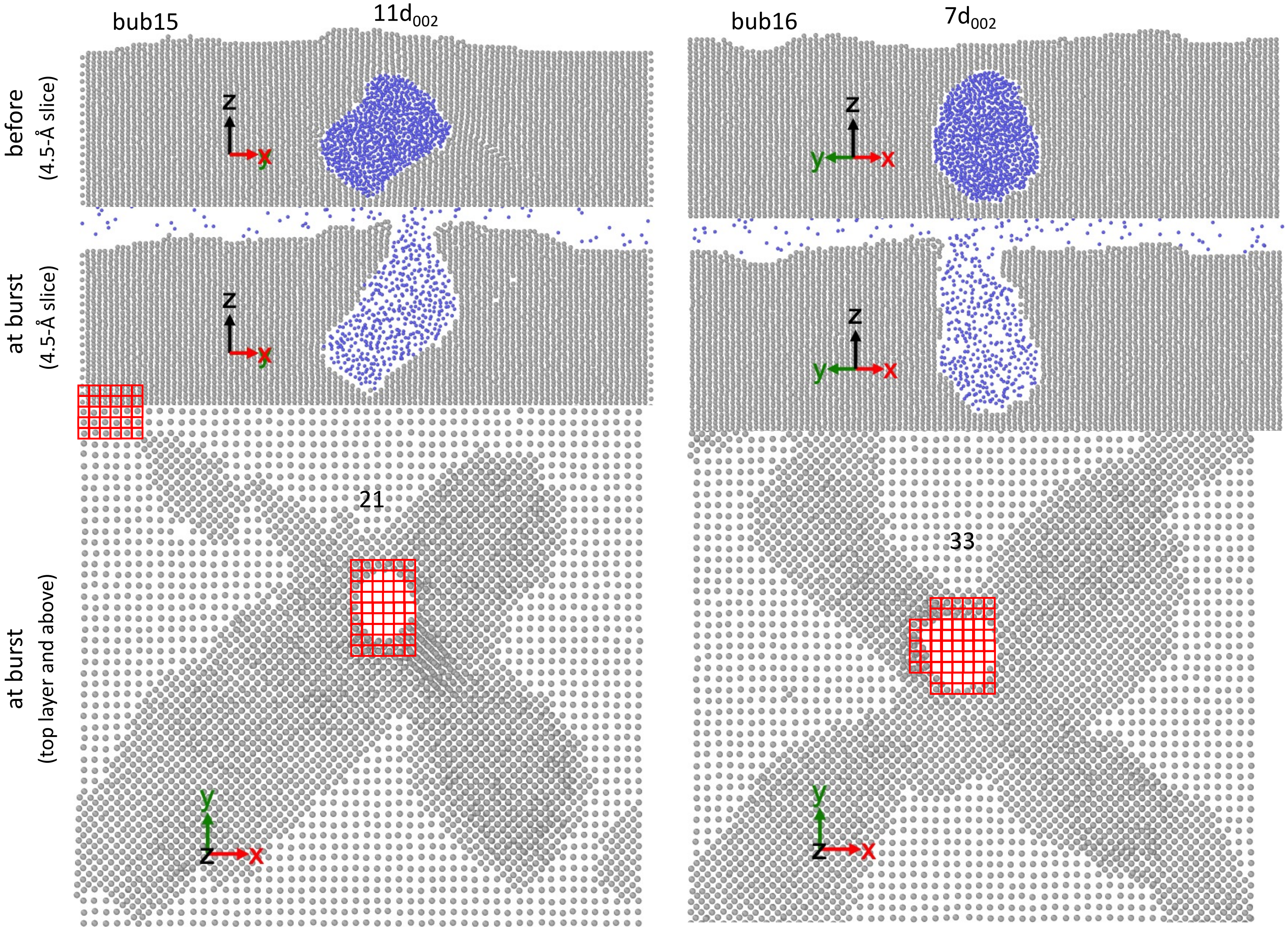

Supplementary Figure 6. Continued.

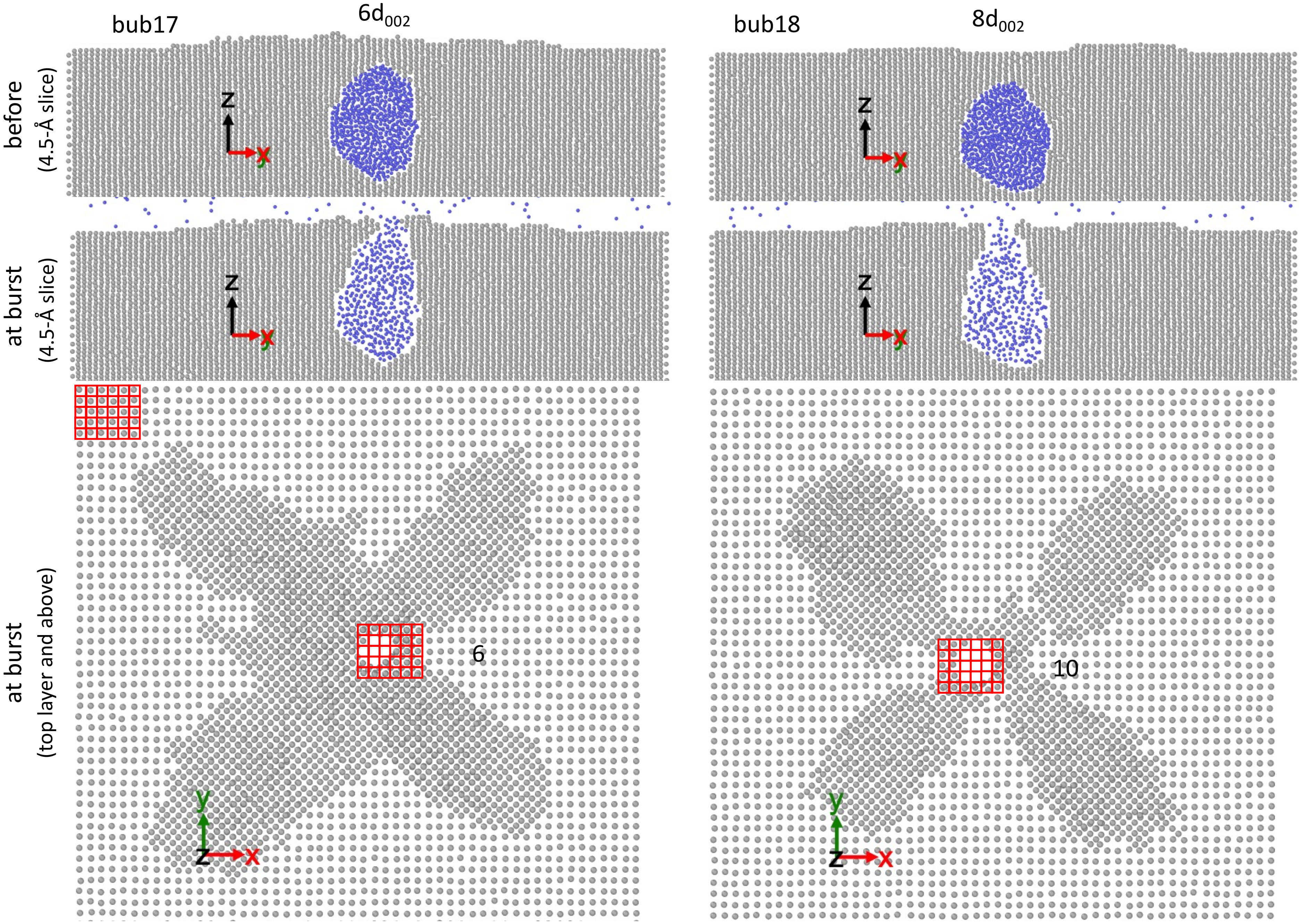

Supplementary Figure 6. Continued.

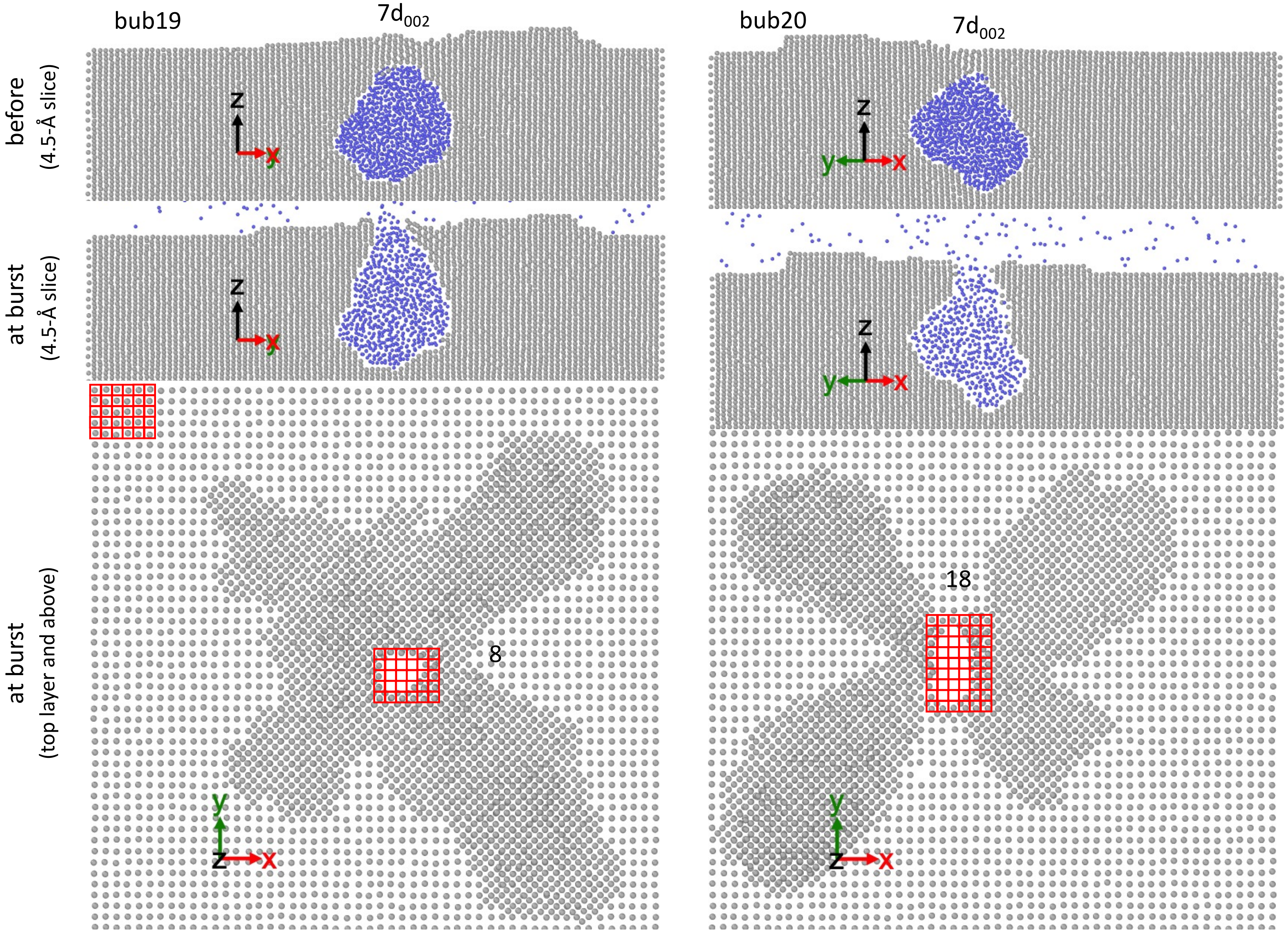

Supplementary Figure 6. Continued.

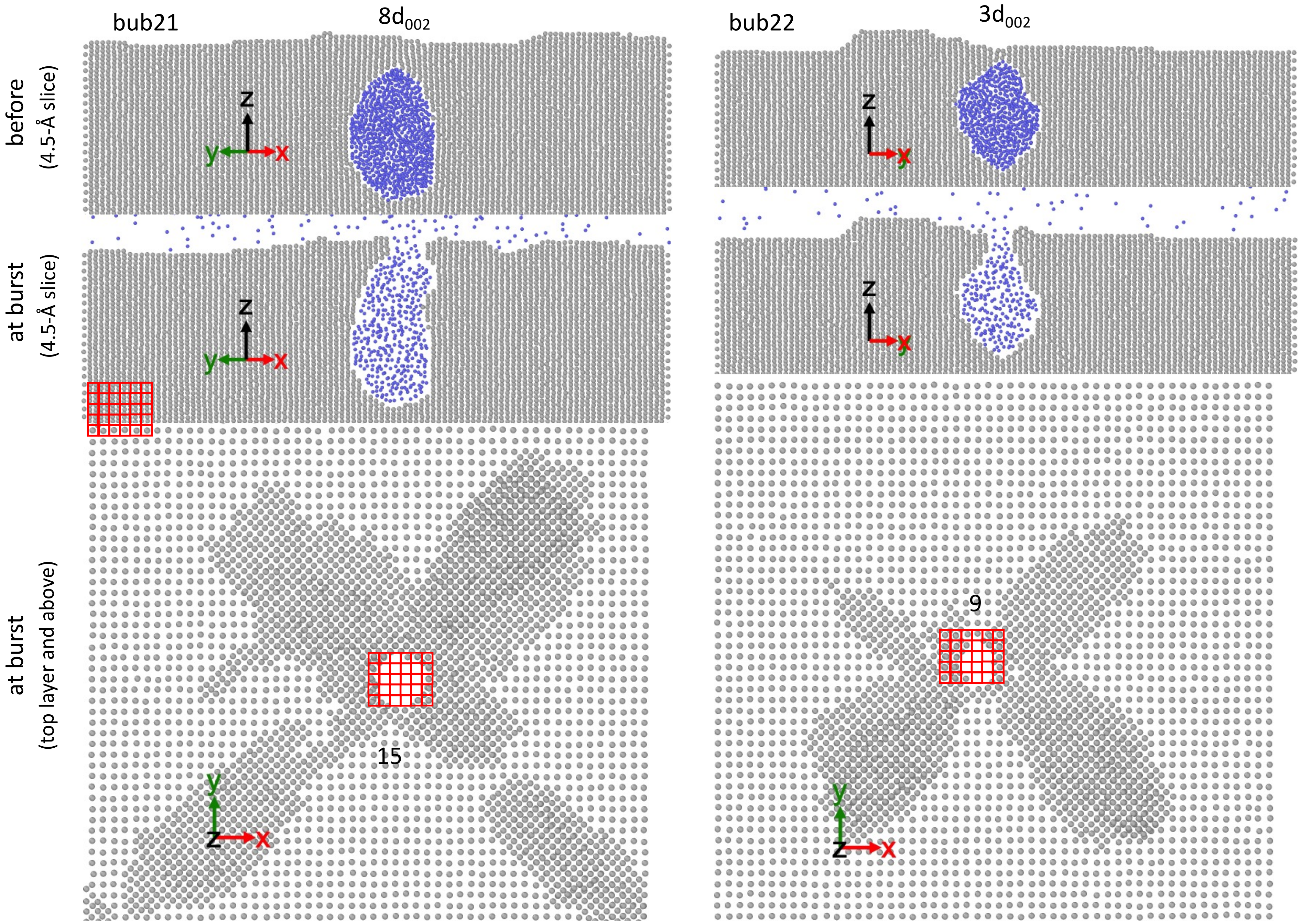

Supplementary Figure 6. Continued.

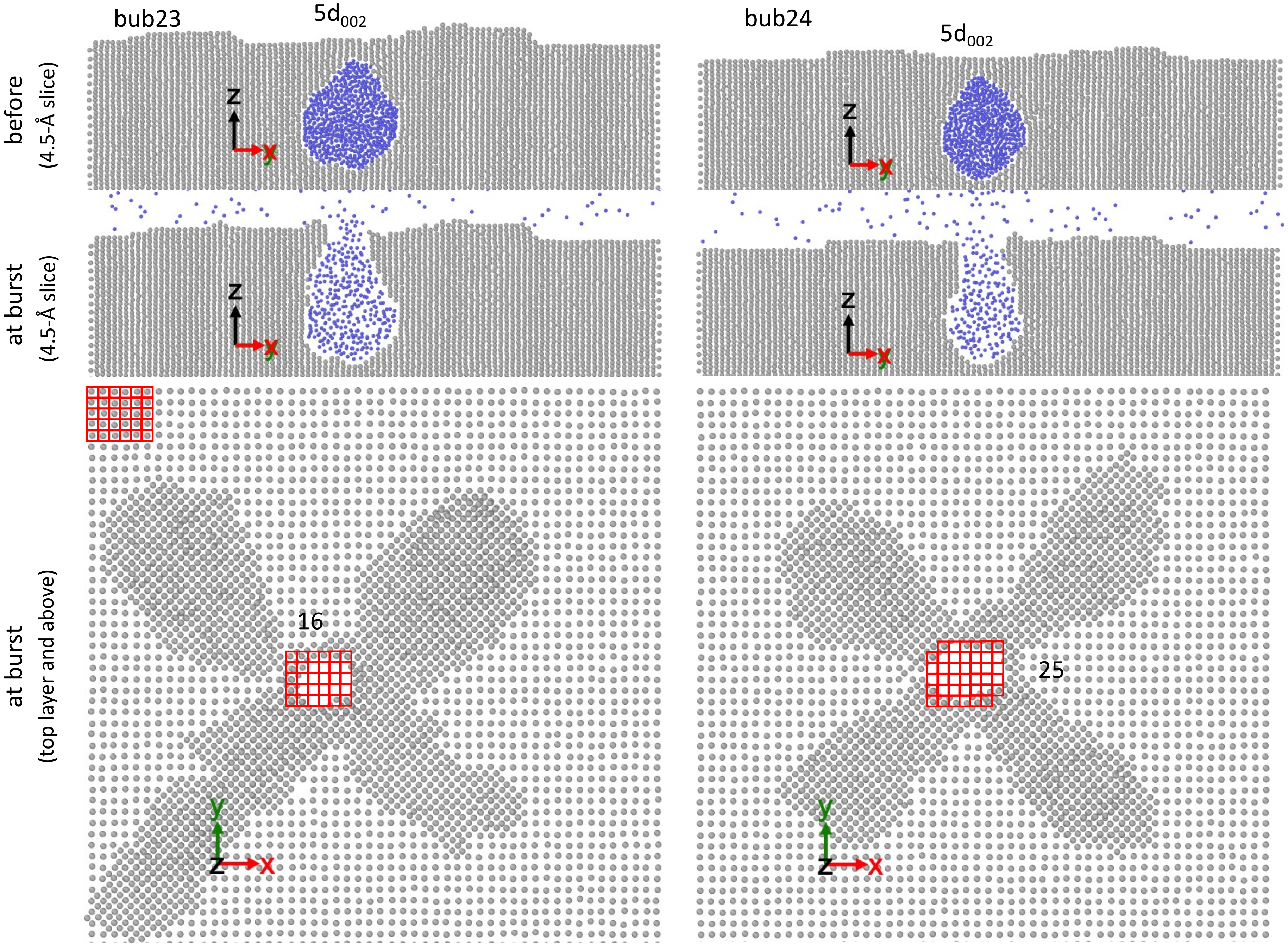

Supplementary Figure 6. Continued.

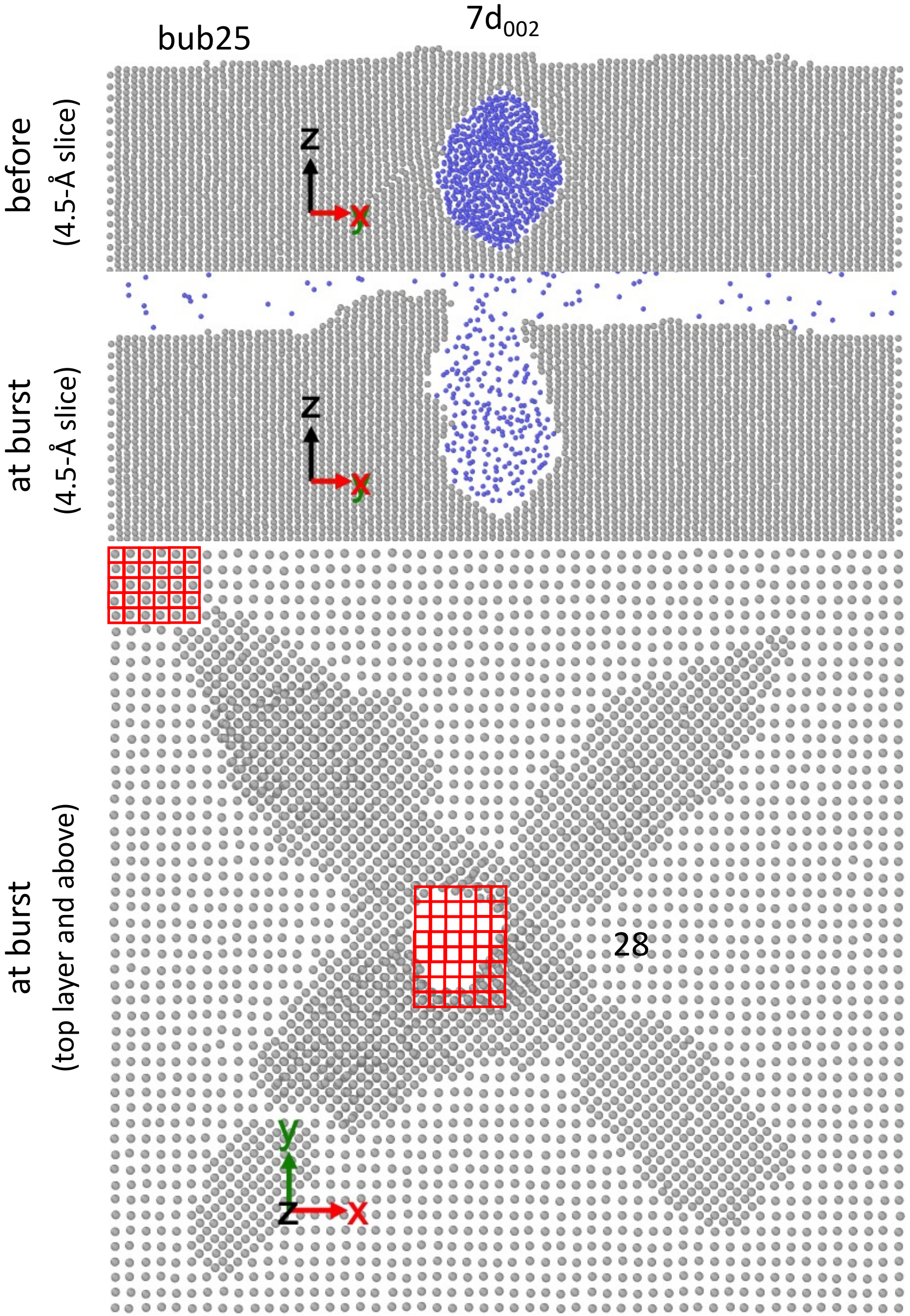

Supplementary Figure 6. Continued.

Supplementary Table 6. Data of thickness of W ligament above the bubble just before bursting (t_{lig}), area of burst hole (A_h), whether or not the bubble is resealed (1: yes, 0: no) after bursting, form bubbles initially nucleated at depth of 25a/2.

bub	t_{lig} (d_{002})	A_h (a^2)	reseal?
1	4	5	0
2	7	15	0
3	12	10	0
4	5	12	0
5	6	15	0
6	7	18	0
7	10	14	0
8	7	14	0
9	3	10	0
10	7	9	0
11	6	12	0
12	10	12	0
13	7	12	0
14	6	16	0
15	11	21	0
16	7	33	0
17	6	6	0
18	8	10	0
19	7	8	0
20	7	18	0
21	8	15	0
22	3	9	0
23	5	16	0
24	5	25	0
25	7	28	0
average	6.8	14.5	0.0

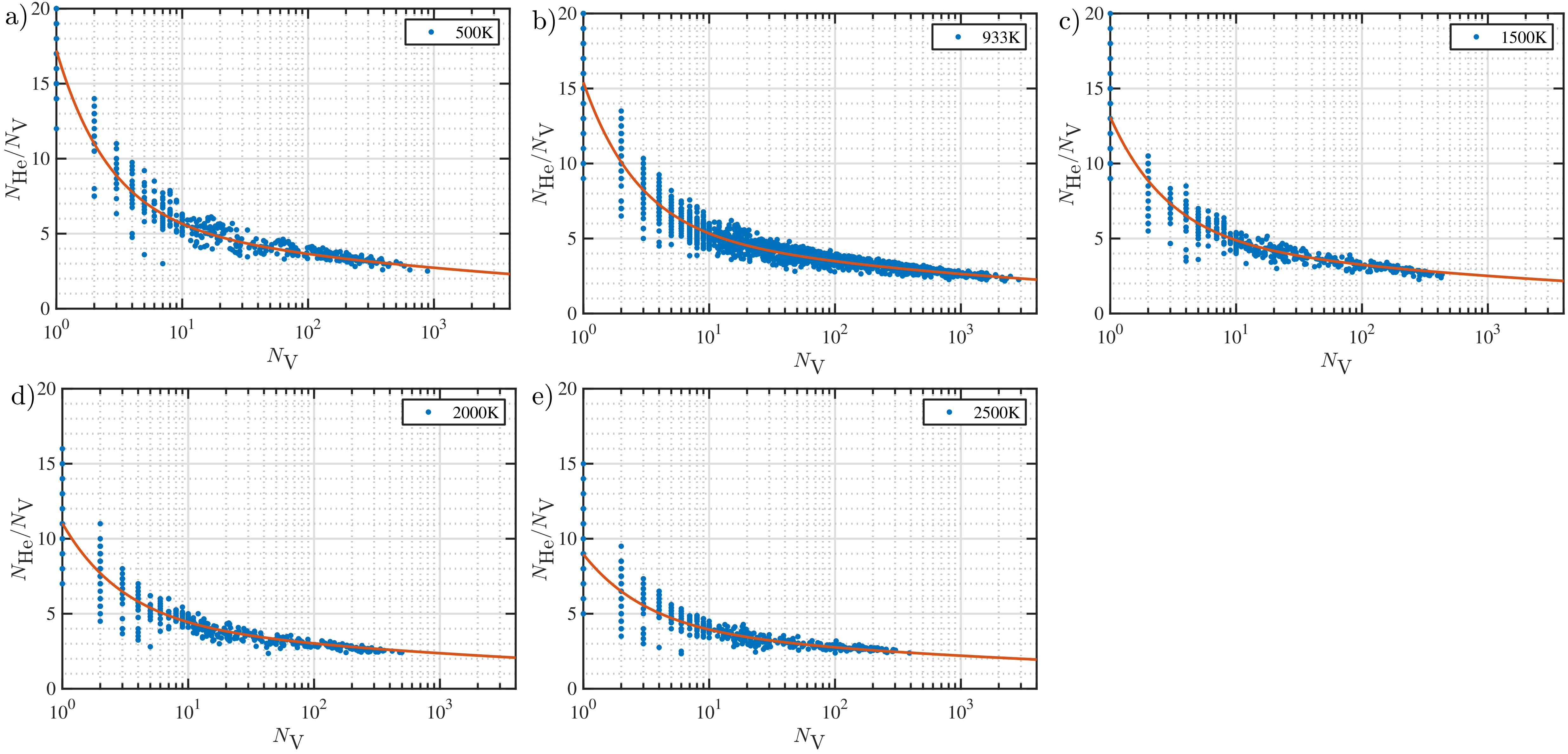

Supplementary Figure 7. Scatter plots of N_{He}/N_V versus N_V at loop punching events at a) 500, b) 993, c) 1500, d) 2000, and e) 2500 K, along with the model (Eq. 2 in the manuscript) predictions represented by the solid lines.

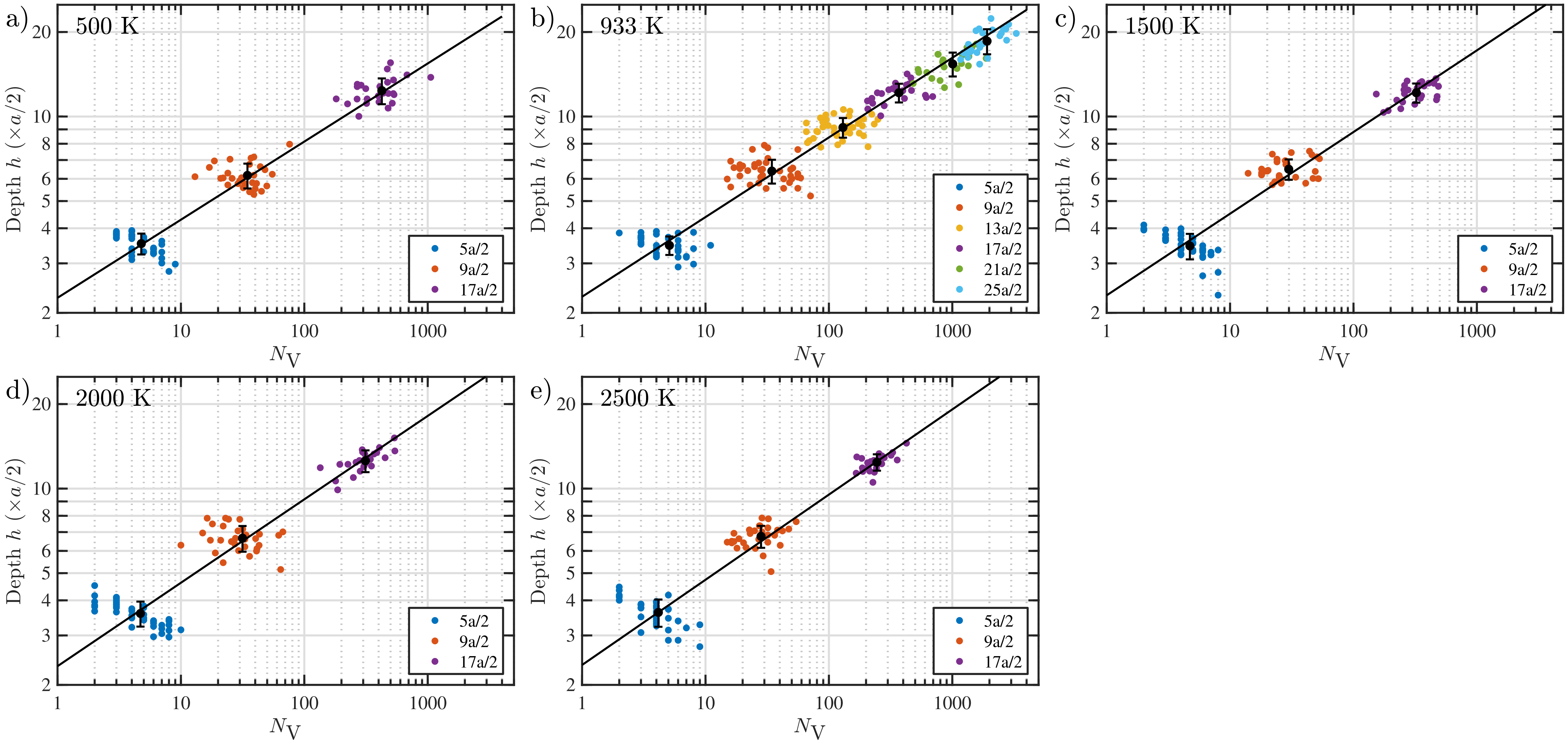

Supplementary Figure 8. Scatter plots of h versus N_V at bubble bursting events at a) 500, b) 993, c) 1500, d) 2000, and e) 2500 K, along with the model (Eq. 4 in the manuscript) predictions represented by the solid lines. Error bars denote standard deviations within each group of bubbles nucleated at the same initial depth. The full data set is used for fitting.

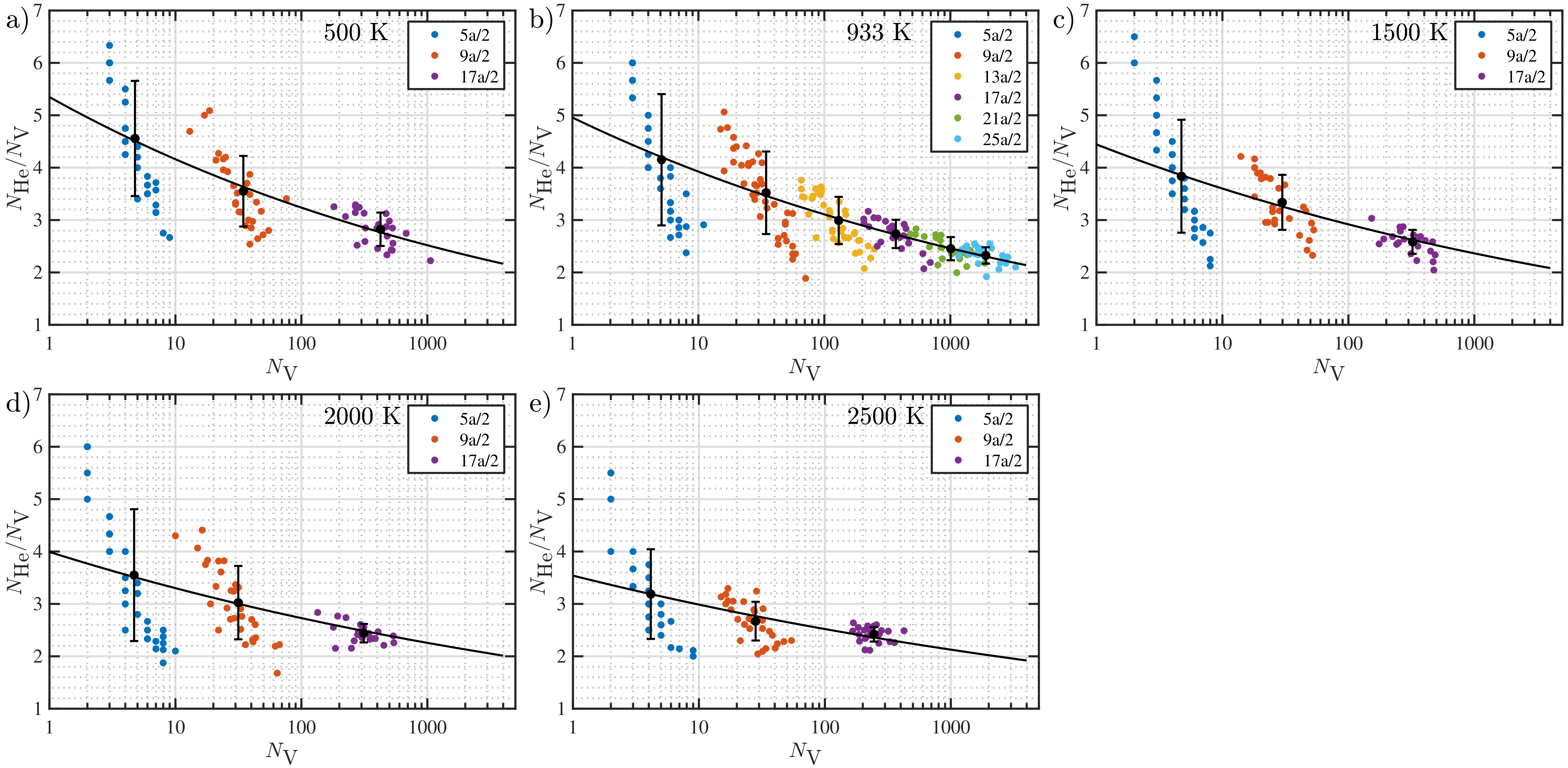

Supplementary Figure 9. Scatter plots of N_{He}/N_V versus N_V at bubble bursting events at a) 500, b) 993, c) 1500, d) 2000, and e) 2500 K, along with the model (Eq. 5 in the manuscript) predictions represented by the solid lines. Error bars denote standard deviations within each group of bubbles nucleated at the same initial depth. The full data set is used for fitting.
